# Low-dose-rate induces more severe cognitive impairment than high-dose-rate in rats exposed to chronic low-dose γ-radiation

**DOI:** 10.3389/fpubh.2024.1387330

**Published:** 2024-05-22

**Authors:** Tianbao Ma, Kexian Li, Wenjuan Sang, Xingyu Liu, Qun Luo, Ye Peng, Mingxing Wang, Xiu Luo, Jingjing Fang, Haijun Wang, Tao Wang, Changjing Zuo

**Affiliations:** ^1^School of Medicine, Shanghai University, Shanghai, China; ^2^Department of Nuclear Medicine, The First Affiliated Hospital of Naval Medical University, Shanghai, China; ^3^Naval Medical Center, Naval Medical University, Shanghai, China

**Keywords:** low-dose γ-irradiation, dose-rate, cognitive impairment, hippocampal inflammation, PI3K–Akt signaling pathway

## Abstract

**Background:**

Owing to the long penetration depth of gamma (γ)-rays, individuals working in ionizing radiation environments are chronically exposed to low-dose γ-radiation, resulting in cognitive changes. Dose rate significantly affects radiation-induced biological effects; however, its role in chronic low-dose γ-irradiation-induced cognitive impairment remains unclear. We aimed to investigate whether chronic low-dose γ-irradiation at low-dose-rate (LDR) could induce cognitive impairment and to compare the cognitive alteration caused by chronic low-dose γ-irradiation at LDR and high-dose-rate (HDR).

**Methods:**

The rats were exposed to γ-irradiation at a LDR of 6 mGy/h and a HDR of 20 mGy/h for 30 days (5 h/day). Functional imaging was performed to assess the brain inflammation and blood–brain barrier (BBB) destruction of rats. Histological and immunofluorescence analyses were used to reveal the neuron damage and the activation of microglia and astrocytes in the hippocampus. RNA sequencing was conducted to investigate changes in gene expression in hippocampus.

**Results:**

The rats in the LDR group exhibited more persistent cognitive impairment than those in the HDR group. Furthermore, irradiated rats showed brain inflammation and a compromised BBB. Histologically, the number of hippocampal neurons were comparable in the LDR group but were markedly decreased in the HDR. Additionally, activated M1-like microglia and A1-like astrocytes were observed in the hippocampus of rats in the LDR group; however, only M1-like microglia were activated in the HDR group. Mechanistically, the PI3K–Akt signaling pathway contributed to the different cognitive function change between the LDR group and HDR group.

**Conclusion:**

Compared with chronic low-dose γ-irradiation at HDR, LDR induced more severe cognitive impairment which might involve PI3K/Akt signaling pathway.

## 1 Introduction

The global population lives in a radiation-exposed environment. However, the changes in exposure to ionizing radiation (IR), primarily induced by X-, α-, β-, and γ-rays, are limited. Nevertheless, exposure to IR for extended periods is inevitable in some occupations, including radiologists, nuclear facility staff, mining workers, soldiers on nuclear submarines, and air force pilots (1, 2). The penetration depth of α- and β-rays is relatively short. Furthermore, lead product-based physical isolation is an effective protective method. In contrast, γ-rays have high penetrability, making it difficult to be fully protected via lead shielding; this generates a virtual chronic low-dose γ-radiation-exposed environment (3). Notably, epidemiological study has revealed that individuals exposed to chronic low-dose radiation are at a higher risk of developing Parkinson’s disease (4).

Low-dose IR (LDIR) is defined as IR at a dose of ≤100 mGy (5). Although the brain is less sensitive to radiation, many studies suggest that low-dose γ-radiation exposure can lead to cognitive changes in animals by affecting hippocampal function (6). In general, radiation exposure can induce hippocampal-dependent impairments in spatial, learning and working, and short-term memory (7, 8). Regarding biological mechanisms, LDIR induces DNA and mitochondrial damage, oxidative stress, and vascular disorders, resulting in hippocampal dysfunction (9–11). Therefore, the pathophysiological state of the hippocampus after radiation exposure may help determine the occurrence and development of cognitive changes.

In previous studies, cognitive impairment models were established using acute low-dose γ-radiation at a high-dose-rate (HDR) (12–14). However, the situation is different when considering radiation at a low-dose-rate (LDR). LDR γ-radiation is defined as radiation exposure at a rate of ≤6 mSv/h (5). The causal relationship between LDR γ-radiation and cognitive impairment remains unclear. However, based on the hormesis theory, LDR radiation can induce some positive biological effects, including immune enhancement and damage repair (15). Nevertheless, whether LDR γ-irradiation imairs, improves, or does not affect cognitive function remains unclear.

In the present study, we elucidated the effects of chronic low-dose γ-radiation at different dose rates on cognitive function and determined the potential mechanisms. Our study provides novel insights into the effects of chronic low-dose γ-radiation on cognitive function and emphasizes the importance of dose rate, which may be useful in establishing new standards for radiation protection.

## 2 Materials and methods

### 2.1 Materials and reagents

The ^60^Co radiation source was provided by the Naval Medical Center, Naval Medical University (Shanghai, China). Shanghai Atom Kexing Pharmaceuticals Co, Ltd. (Shanghai, China) provided ^18^F-fludeoxyglucose (FDG) and ^99m^Tc-NaTcO_4_. The First Affiliated Hospital of Naval Medical University (Shanghai, China) provided sodium pentobarbital and physiological saline. Formaldehyde, 4% paraformaldehyde, anhydrous ethanol, and acetic acid were purchased from Aladdin Co, Ltd. (Shanghai, China). Toluidine blue, neutral gum, xylene, fluoro-Jade B (FJB), phosphate-buffered saline (PBS), bovine albumin (BSA), and Trizol were obtained from Servicebio Co, Ltd. (Shanghai, China). Sodium dodecyl sulfate (SDS) polyacrylamide gel, protein extraction reagents, bicinchoninic acid (BCA), enhanced chemiluminescence luminescent (ECL) reagent and polyvinylidene fluoride (PVDF) membrane were purchased from Beyotime Biotechnology Co, Ltd. (Shanghai, China). Primary antibodies against phosphorylated phosphatidylinositol 3-kinase (p-PI3K) and complement 3 (C3) were provided by BIOSS Co, Ltd. (Beijing, China). The primary antibodies against Glial fibrillary acidic protein (GFAP), ionized calcium-binding adapter molecule 1 (Iba-1), cluster of differentiation 86 (CD86), phosphorylated protein kinase B (p-Akt), PI3K, Akt and secondary antibodies were purchased from Servicebio Co, Ltd. (Shanghai, China).

### 2.2 Animals and irradiation

**SCHEME 1:**
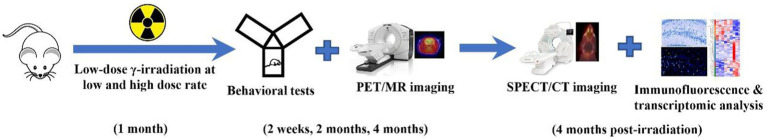
The experimental design.

Thirty-six female Sprague Dawley (SD) rats aged 6–8 weeks were housed in a room with a temperature of 22°C–25°C and a 12:12 light/dark cycle. They were given *ad libitum* access to food and water. The rats were randomly divided into three groups, with 12 rats in each group: control, LDR, and HDR groups. After allowing the rats to adapt to the environment for 1 week, they were exposed to whole-body irradiation with ^60^Co γ-rays. The rats in the LDR and HDR groups were continuously irradiated for 30 days (5 h/day) at dose rates of 6 and 20 mGy/h, with a total dose of 0.9 Gy and 3 Gy, respectively. The rats in the control group were raised in the same environment but were not irradiated. Scheme 1 illustrates the experimental schedule.

### 2.3 Open field test (OFT)

All behavioral tests were conducted in a quiet environment from 9:00 a.m. to 5:00 p.m. at 2 weeks, 2 months, and 4 months post-irradiation.

The open field test was conducted to evaluate anxiety and depression levels. Rats were individually placed into the center of a black plastic square box measuring 80 cm × 80 cm × 40 cm and allowed to freely move for 5 min. The main index of interest was the time that the animal spent in the central areas. Owing to the preference for darkness and light aversion, rats with abnormal emotions tend to spend less time in the center.

### 2.4 Y-maze test

The Y-maze test is used to assess the working and learning memory of rats. The equipment used in this study comprised three black plastic arms (50 cm × 10 cm × 30 cm) placed at 120° to one another. There were two test phases. In the first phase, one of the three arms was closed, and the animals were allowed to explore the other two arms for 5 min. Phase two began 2 h after phase one. In this phase, the closed arm, called a novel arm, was opened. The animals were allowed to freely explore all three arms for 5 min. The experimental results were evaluated by measuring the percentage of time spent by the animals exploring the novel arm in their total exploration time during phase two.

### 2.5 Spontaneous alternation behavior (SAB)

SAB was used to evaluate the spatial reference memory of the animals. This test is based on the innate curiosity of the rats. The same Y-maze was used in both the SAB and Y-maze tests. The animals were placed at the entrance of one of the three arms and allowed to freely explore the maze for 10 min. When the rats continuously explored three different arms, they were considered to have finished one alternation. The maximum number of alternations was calculated by subtracting 2 from the number of arms explored by the rat. The alternation percentage was defined as the proportion of the total number of alternations to the maximum number of alternations.

### 2.6 Novel object recognition (NOR)

NOR is an efficient approach for evaluating the episodic memory of rats. Pilot experiments were conducted before NOR and persisted for 2 days. The animal was daily placed into the test box (same as the OFT box) and allowed to explore for 10 min. The experiment comprised two stages: familiarization and testing. Each stage lasted 10 min, with a 1 h interval between both stages. During the familiarization phase, animals were allowed to freely explore the box, with two identical objects placed in the center. In the testing stage, one of the two objects was replaced with a new object of similar size. The animal was allowed to explore the box for the same duration. Discrimination index (DI) was used to evaluate the presence of memory defects, as calculated using the following formula: (TN − TF) / (TN + TF), where TN denotes the duration of exploration of familiar objects, whereas TF denotes the duration of exploration of new objects. The range of DI is between −1 and 1. When the index approaches 1, animals require more time to explore new objects. In contrast, when the index approaches −1, animals take more time to explore familiar objects.

### 2.7 ^18^F-FDG positron emission computed tomography/magnetic resonance imaging (PET/MR)

PET/MR imaging (Biograph mMR, Siemens, Germany) was completed within 1 week after each behavioral experiment. ^18^F-FDG was used to evaluate glucose metabolism in rats. To normalize baseline blood glucose levels, the animals were subjected to fasting for 8 h before imaging. Rats were administered 150 μL of ^18^F-FDG at a dose of 0.6 μCi/g, followed by a 30 min uptake period; during this period, the animals were allowed to freely move. Subsequently, the rats were intraperitoneally anesthetized. Then, they were placed in the prone position on a scanner bed and scanned for 30 min as per the standard imaging protocol. T2-weighted images were synchronized with PET images and imaging was continued for 60 min, providing the detailed anatomical structure and pathological changes. The maximum standardized uptake value (SUVmax) was used to quantitatively assess glucose metabolism in the brain.

### 2.8 ^99m^Tc-NaTcO_4_ single-photon emission computed tomography/computed tomography imaging (SPECT/CT)

To determine the integrity of the blood–brain barrier (BBB), ^99m^Tc-NaTcO_4_ SPECT/CT imaging (Symbia T16, Siemens, Germany) was performed. The rats were anesthetized and administered 200 μL of ^99m^Tc-NaTcO_4_ at a dose of 0.6 μCi/g. SPECT/CT imaging was performed after 20 min using a clinical SPECT/CT scanner. The regions of interest were drawn over the brain to obtain the average radioactivity count.

### 2.9 Nissl staining

After imaging, animals were anesthetized using sodium pentobarbital (100 mg/kg) and perfused with 100 mL of cold physiological saline solution and 300 mL of 4% formaldehyde at 4°C via the left ventricle. Subsequently, the animals were decapitated, and their brains were extracted and placed in 4% paraformaldehyde at 4°C for 48 h. The brain samples were embedded in paraffin wax and sectioned to a thickness of 5 μm. The sections were dewaxed and dehydrated using gradient anhydrous ethanol and used for staining.

Nissl staining was performed to detect neuronal damage in the hippocampus. The brain sections were stained with toluidine blue at room temperature for 45 min. Excess dye was rinsed off with distilled water. The sections were then differentiated with 75% anhydrous ethanol for 30 min, followed by dehydration with gradient anhydrous ethanol and xylene. Finally, the sections were sealed with neutral gum. The section was observed using an upright fluorescence microscope (NIKON ECLIPSE C1, Nikon, Japan).

### 2.10 FJB staining

FJB staining was conducted to evaluate neuronal degeneration and the presence of activated glial cells. Brain sections were immersed in a solution comprising FJB mixed at a ratio of 1:50–1:100 (0.1% glacial acetic acid as the solvent) and incubated for 20 min at room temperature. Then, the sections were rinsed with running water and allowed to dry overnight. The sections were stained with 4′,6-diamidino-2-phenylindole (DAPI), followed by rinsing with PBS, dehydration with xylene for 2 min, and sealing with neutral gum.

### 2.11 Immunofluorescence analysis

Brain sections were treated with boiling citric acid for 30 min for antigen removal and then cooled at room temperature. The sections were then immersed in a PBS solution and shaken to rinse. The sections were sealed with 5% BSA for 30 min. Next, the section was incubated with primary antibodies overnight at 4°C, secondary antibodies in the dark at room temperature for 50 min, and DAPI in the dark for 10 min. Between each incubation, the sections were rinsed with PBS solution for 5 min; this was repeated three times. Finally, the sections were incubated with a spontaneous fluorescence quenching agent for 5 min, washed with distilled water for 20 min, and sealed with an anti-fluorescence quenching sealing agent.

### 2.12 RNA-seq analysis

RNA-seq analysis was conducted in cooperation with Shanghai Majorbio Bio-pharm Biotechnology Co, Ltd. (China). After functional imaging, animals were decapitated, followed by harvesting the hippocampus from the brain. Total RNA was extracted using QIAzollysisReagent (Qiagen, Germany), followed by purification using an RNA purification kit (Majorbio, China). The Agilent 2,100 bioanalyzer (Agilent, United States) was used for quantification. Superscript double-stranded cDNA synthesis kit (Invitrogen, United States) was used to synthesize the cDNA. Illumina^®^ Stranded mRNA Prep, Ligation (Illumina, United States) was used for library construction. Quantification was performed using Qubit 4.0. The RNA-seq library was sequenced using NovaSeq 6,000 sequencer (Illumina). Fastp software was used to trim the sequence reads. HISAT2 software was used to align the reads to the reference genome. DEGseq software was used to perform differential expression analysis. The threshold was set as follows: fold change >1.5 and *p* < 0.05. SciPy was used to perform functional enrichment and the Kyoto Encyclopedia of Genes and Genomes (KEGG) was referenced.

### 2.13 Western blotting

After mixing with pre-cooled protein extraction reagents, the hippocampal tissue was homogenized and centrifuged to obtain the protein solution, whose concentration was measured using BCA method. The target protein was separated using sodium dodecyl sulfate polyacrylamide gel electrophoresis (SDS-PAGE) and then transferred onto a PVDF membrane. Subsequently, the PVDF membrane was blocked in fat-free milk for 2 h, followed by incubating with the primary antibody at 4°C overnight. Thereafter, the membrane was washed with TBST for three times, and incubated with the secondary antibody at 37°C for 2 h. After washing with TBST twice, the membrane was sink in the ECL reagent, and then exposed to obtain the optimal chemiluminescence signal in the dark. The relative expression of targeted protein was analyzed using ImageJ software.

### 2.14 Statistical analysis

Data were expressed as mean ± standard error of the mean (SEM) or median (min, max) for at least three independent experiments. SPSS Statistics 26 was used to perform statistical analyses. GraphPad 9 was used to create statistical charts. For comparing the differences, one-way analysis of variance was used for normal distribution with two or more groups, whereas the Kruskal–Wallis test was used for non-normal distribution and two or more groups. A *p*-value of <0.05 was used to indicate significant difference.

## 3 Results

### 3.1 Chronic low-dose γ-radiation at LDR and HDR resulted in different behavioral performances among rats

**Figure 1:**
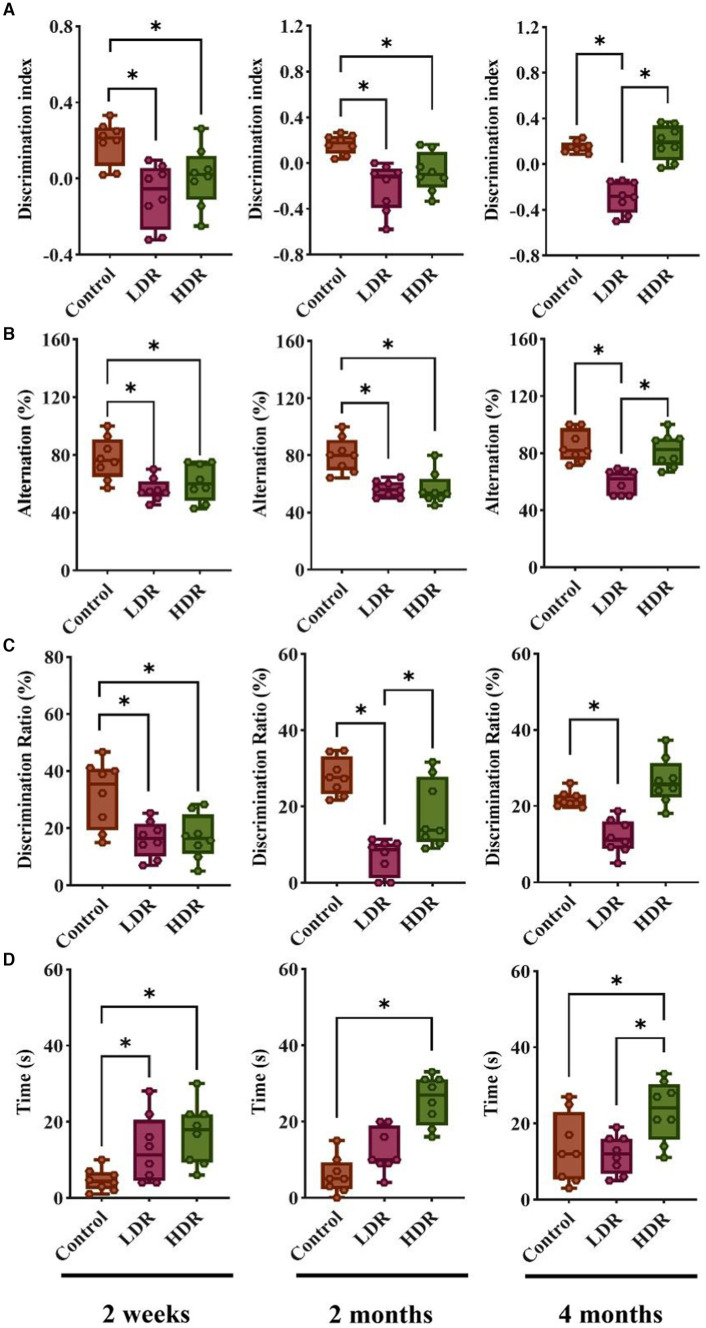
Chronic low-dose γ-radiation exposure at low-dose-rate (LDR) and high-dose-rate (HDR) caused different behavioral performances in rats. **(A)** Discrimination index of rats in novel object recognition. **(B)** Alternation percentage of rats in spontaneous alternation behavior. **(C)** Discrimination ratio of rats in Y-maze. **(D)** The time of rats spent in center arear in open field test. Data were presented as median (min, max). Kruskal-Wallis test was used for statistical analysis (*n* = 8). *p < 0.05.

In the NOR and SAB test, at 2 weeks and 2 months after irradiation, the discrimination index and alternation percentage of the rats in the LDR and HDR groups were remarkably decreased compared with those in the control group; this indicates that chronic low-dose γ-radiation exposure impairs the spatial reference memory and investigating episodic memory of rats. Interestingly, the decrease in the two indexes observed between the HDR and control groups was no longer observed at 4 months after irradiation (Figures 1A,B).

The Y-maze test also revealed significant decreases in the discrimination ratio of the novel arm of the LDR and HDR groups compared with the control group at 2 weeks after irradiation; this suggests that chronic low-dose γ-irradiation impairs the working and learning memory of rats. However, the decrease observed between the HDR and control groups disappeared after 2 months. Interestingly, the significant decrease between the LDR and HDR groups and between the LDR and control groups remained until the end of the 4-month experiment (Figure 1C).

During all three periods, the time spent in the central area significantly increased in the HDR and control groups in the open field test; this suggests that no instances of anxiety or depression were reported after irradiation. However, the time spent in the central area notably increased for the LDR group; nevertheless, it was restored at 2 and 4 months after irradiation (Figure 1D).

Collectively, the behavioral test revealed distinct cognitive changes between both groups of irradiated animals. The rats in the LDR group demonstrated persistent cognitive impairment until 4 months; however, the cognitive dysfunction of the rats in the HDR group had already recovered at 4 months. Furthermore, none of the irradiated animals displayed signs of anxiety or depression.

### 3.2 Chronic low-dose γ-irradiation at LDR and HDR induced potential brain inflammation and BBB impairment in rats

**Figure 2:**
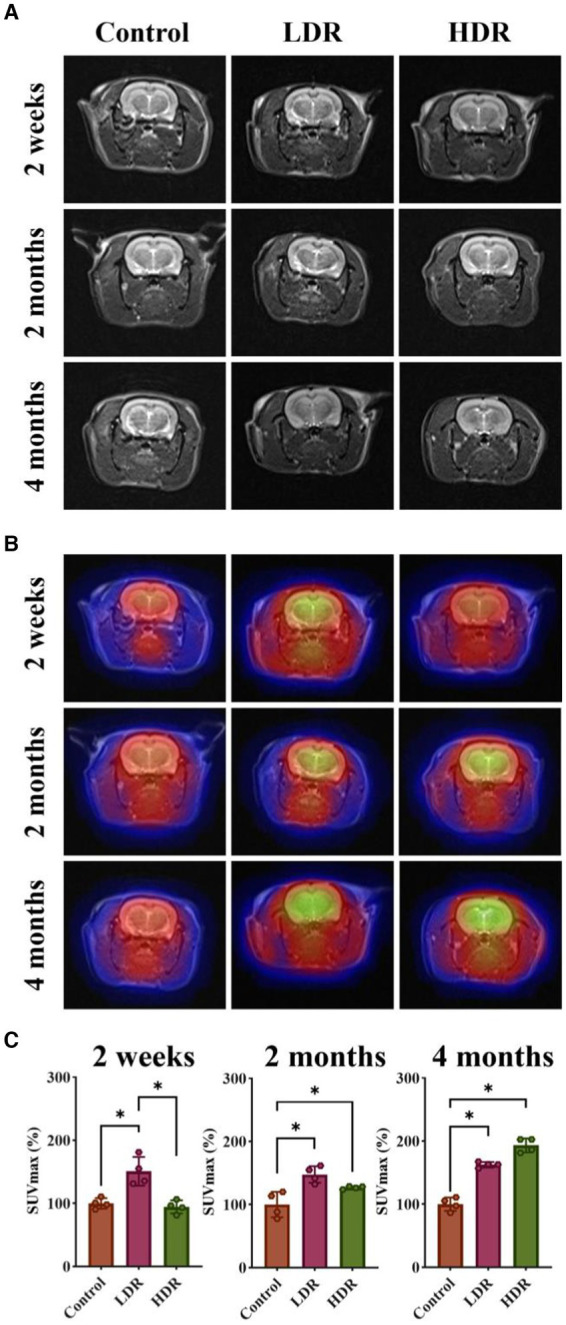
^18^F-FDG PET/MR imaging showed brain inflammation of rats exposed to low-dose γ-radiation. **(A)** T2 weighted images of rat brains. **(B)** ^18^F-FDG PET/MR fusion images of rat brains. **(C)** Relative SUVmax of rat brains. Data were given as mean ± SEM. One-way ANOVA test followed by LSD was used for statistical analysis (*n* = 4). *p < 0.05.

FDG can be transported into the cells via the same pathway as glucose. However, its metabolism is challenging; therefore, it remains in the cells. In general, inflammatory tissues have a higher glucose metabolism, resulting in increased FDG uptake. In the present study, we observed that the ^18^F-FDG uptake of the brain was significantly higher in the two irradiated groups than in the control group, except for the HDR group at 2 weeks after irradiation. Interestingly, T2-weighted images did not reveal any noticeable structural changes in the brain (Figures 2A,B). However, SUVmax measurement revealed increased radioactive uptake in the two irradiated groups compared with the control group (Figure 2C). Collectively, these findings suggest the presence of brain inflammation in both irradiated groups.

**Figure 3:**
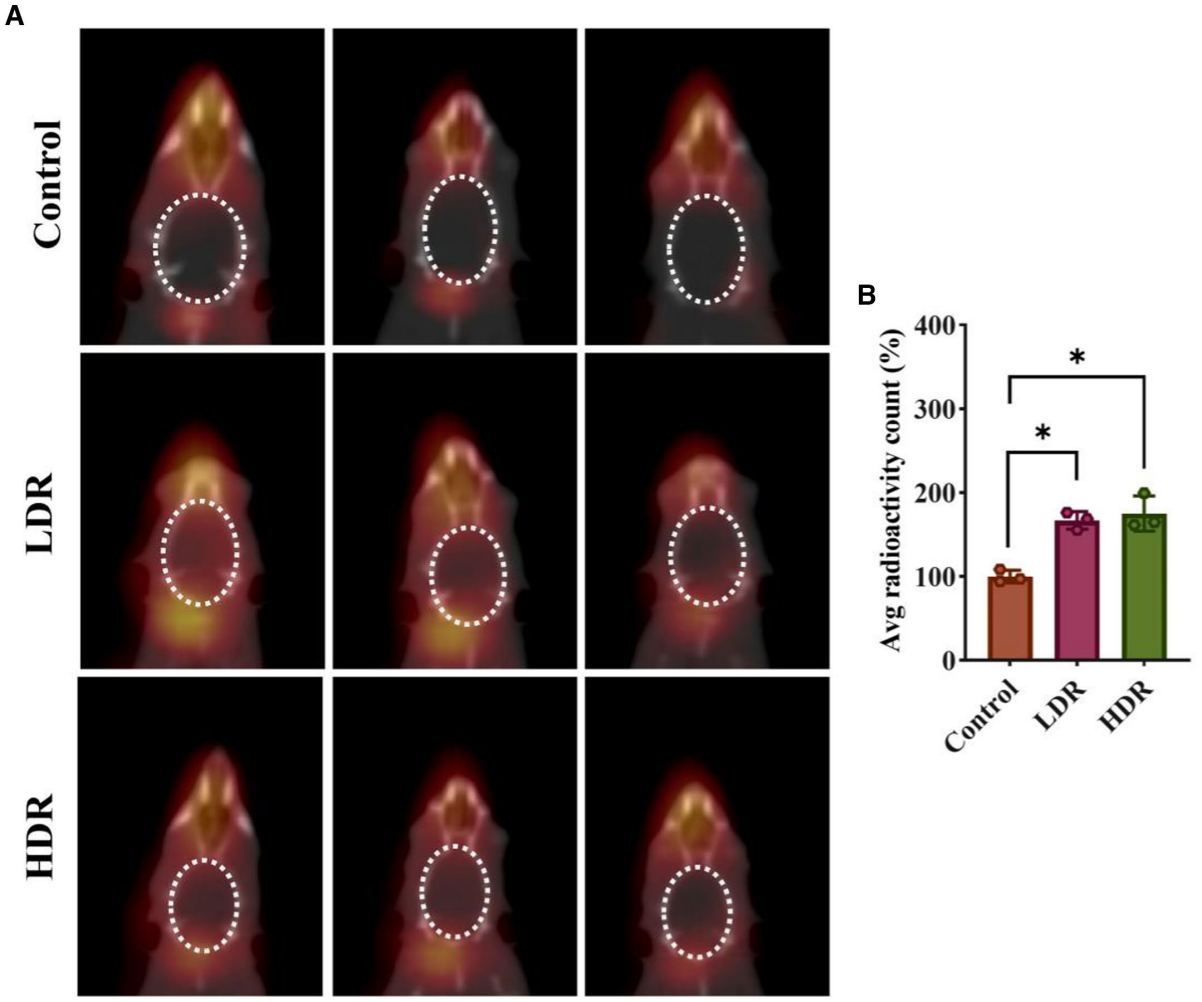
^99m^Tc-NaTcO_4_ SPECT/CT imaging showed blood–brain barrier (BBB) impairment of irradiated rat brains. **(A)** ^99m^Tc-NaTcO_4_ SPECT/CT fusion images of rat brains. The white dashed area represents the rat brain. **(B)** Relative average radioactivity count of rat brain parenchyma in ^99m^Tc-NaTcO_4_ SPECT imaging. Data were given as mean ± SEM. One-way ANOVA test followed by LSD was used for statistical analysis (*n* = 3). *p < 0.05.

Tc^7+^ cannot enter the normal brain. An increase in Tc^7+^ content in the brain indicates that BBB integrity has been damaged. We observed that ^99m^Tc-NaTcO_4_ accumulation in the brain was remarkedly increased in the two irradiated groups compared with the control group (Figure 3). Overall, our results suggest the occurrence of brain inflammation and BBB impairment in the irradiated rats.

### 3.3 Synaptic plasticity alterations and neuronal damage differences in the hippocampus

**Figure 4:**
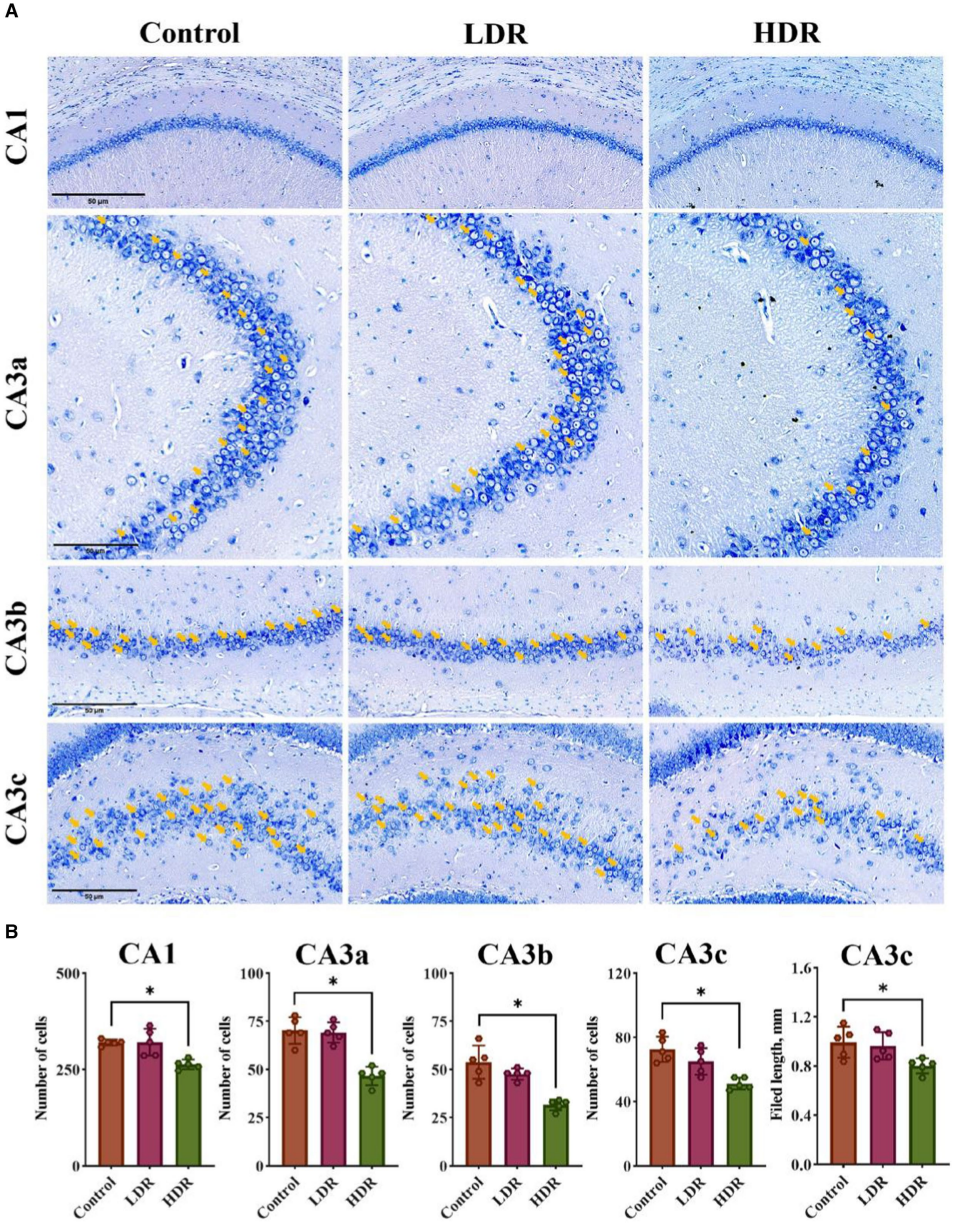
Nissl-staining revealed the difference of neuronal damage in hippocampus between the LDR and HDR groups. **(A)** The representative images of Nissl-stained section in CA1, CA3a, CA3b and CA3c filed of rat hippocampus. Arrowheads indicate living neurons. **(B)** The number of the living neurons in CA1, CA3a, CA3b and CA3c filed and the length in CA3c of rat hippocampus. Data were given as mean ± SEM. One-way ANOVA test followed by LSD was used for statistical analysis (*n* = 5). *p < 0.05. Scale, 50 μm.

Nissl staining was performed to quantitatively analyze the number of living neurons in the cell layer of hippocampal pyramidal neurons, including the CA1, CA3a, CA3b, and CA3c regions. Compared with the control group, the number of neurons was stable in the LDR group; however, a substantial decrease in all four regions was observed in the HDR group (Figures 4A,B). These findings indicate that chronic low-dose γ-radiation at HDR induces neuronal damage in the rat hippocampus.

**Figure 5:**
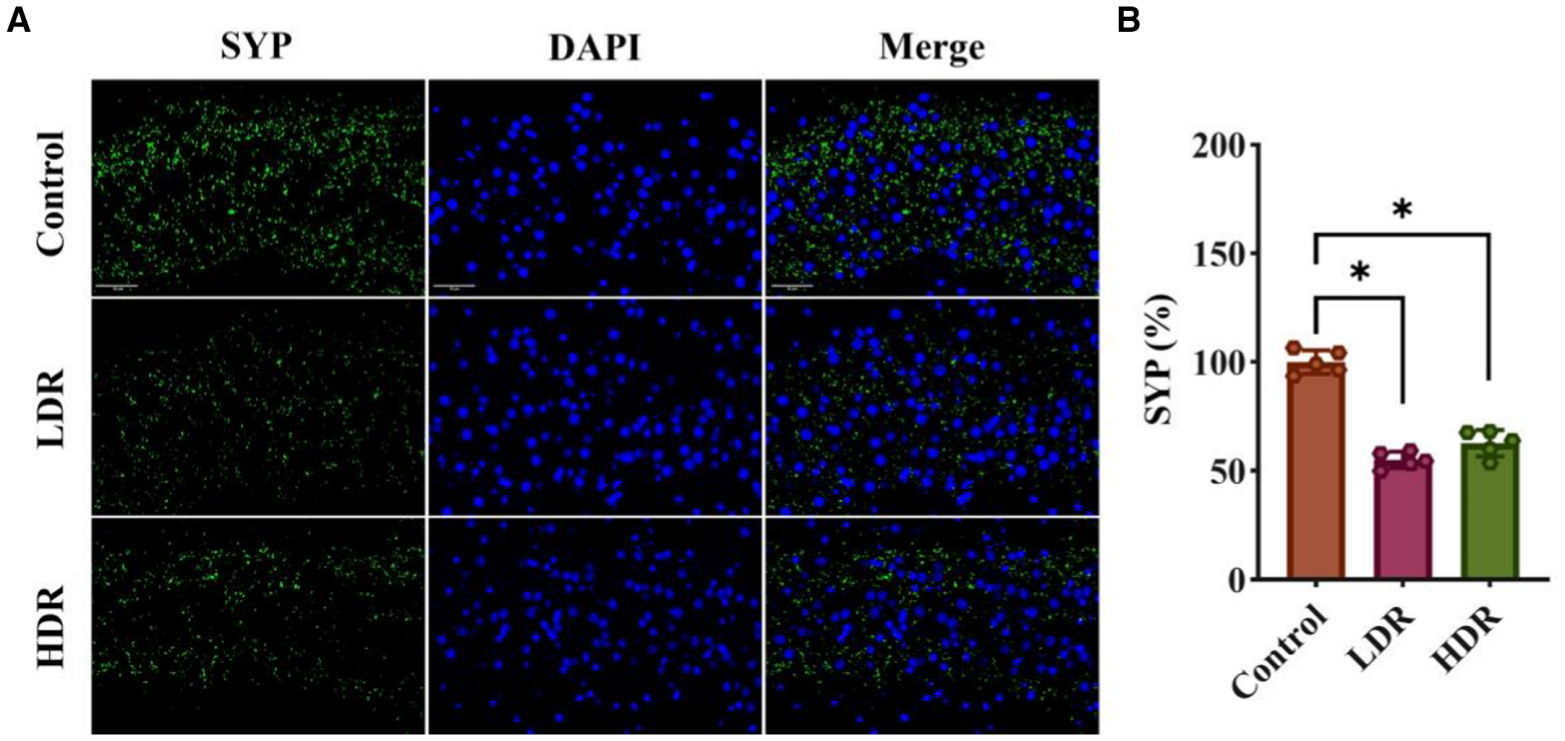
SYP immunofluorescence assay revealed the synaptic plasticity changes in the hippocampus of irradiated rats. **(A)** The representative images of SYP immunofluorescence section. **(B)** Relative fluorescent intensities of SYP^+^ cells in hippocampus. Data were given as mean ± SEM. One-way ANOVA test followed by LSD was used for statistical analysis (*n* = 5). *p < 0.05. Scale, 50 μm.

Synaptophysin (SYP) plays an important role in maintaining synaptic function and is considered an indicator of changes in synaptic plasticity. Figure 5 shows that the fluorescence intensity of SYP in the hippocampus of irradiated rats significantly decreased compared with that of control rats, suggesting changes in synaptic plasticity after chronic low-dose γ-irradiation.

### 3.4 Distinct inflammatory environment in the hippocampus

**Figure 6:**
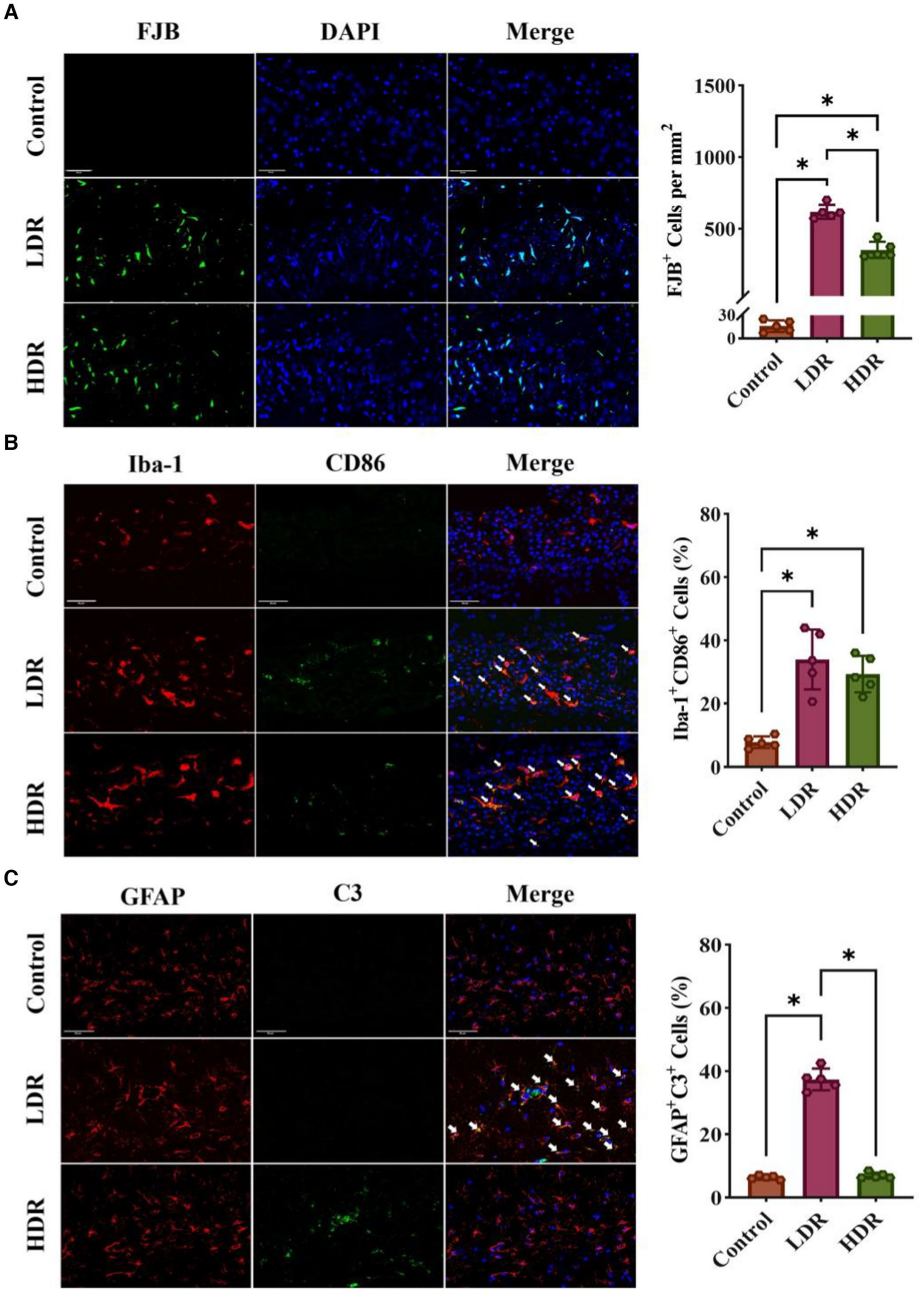
Immunofluorescence assay revealed different inflammatory response in hippocampus of rats between the LDR and HDR groups. **(A)** The representative images of FJB-stained section and cell density of FJB^+^ cells in hippocampus. **(B)** The expression of CD86 in astrocytes in hippocampus. Arrowheads indicate Iba-1^+^ CD86^+^ cells. **(C)** The expression of C3 in astrocytes in hippocampus. Arrowheads indicate C3^+^ GFAP^+^ cells. Data were given as mean ± SEM. One-way ANOVA test followed by LSD was used for statistical analysis (*n* = 5). *p < 0.05. Scale, 50 μm.

FJB is a fluorescent dye that can label desaturated neurons, activated microglia, and astrocytes. FJB staining revealed that the number of activated glial cells significantly increased in the hippocampus of both irradiated groups; however, the number of desaturated neurons did not significantly increase. Moreover, the number of activated glial cells was higher in the LDR group than in the HDR group (Figure 6A).

To quantify the number of activated inflammatory M1-type microglia in the hippocampus, we performed dual fluorescence staining. Microglia and inflammatory M1-type microglia were identified using Iba-1 and CD86 as markers, respectively. The number of inflammatory M1-type microglia (double-positive Iba-1^+^ CD86^+^) was significantly increased in the irradiated groups (Figure 6B).

Using dual fluorescence staining, we measured the number of activated neurotoxic A1-type astrocytes in the hippocampus. GFAP and C3 proteins were used as the markers for astrocytes and neurotoxic A1-type astrocytes. Compared with the control group, the number of neurotoxic A1-type astrocytes (double-positive GFAP^+^ C3^+^) significantly increased in the LDR group. However, no changes were observed in the HDR group compared with the control group (Figure 6C).

Overall, immunofluorescence analysis revealed different inflammatory environments in the hippocampus between the LDR and HDR groups. The number of activated glial cells was significantly higher in the LDR group than in the HDR group. Furthermore, both M1-type microglia and A1-type astrocytes were activated in the LDR group; however, only M1-type microglia were activated in the HDR group.

### 3.5 Chronic low-dose γ-irradiation involves the PI3K–Akt signaling pathway

**Figure 7:**
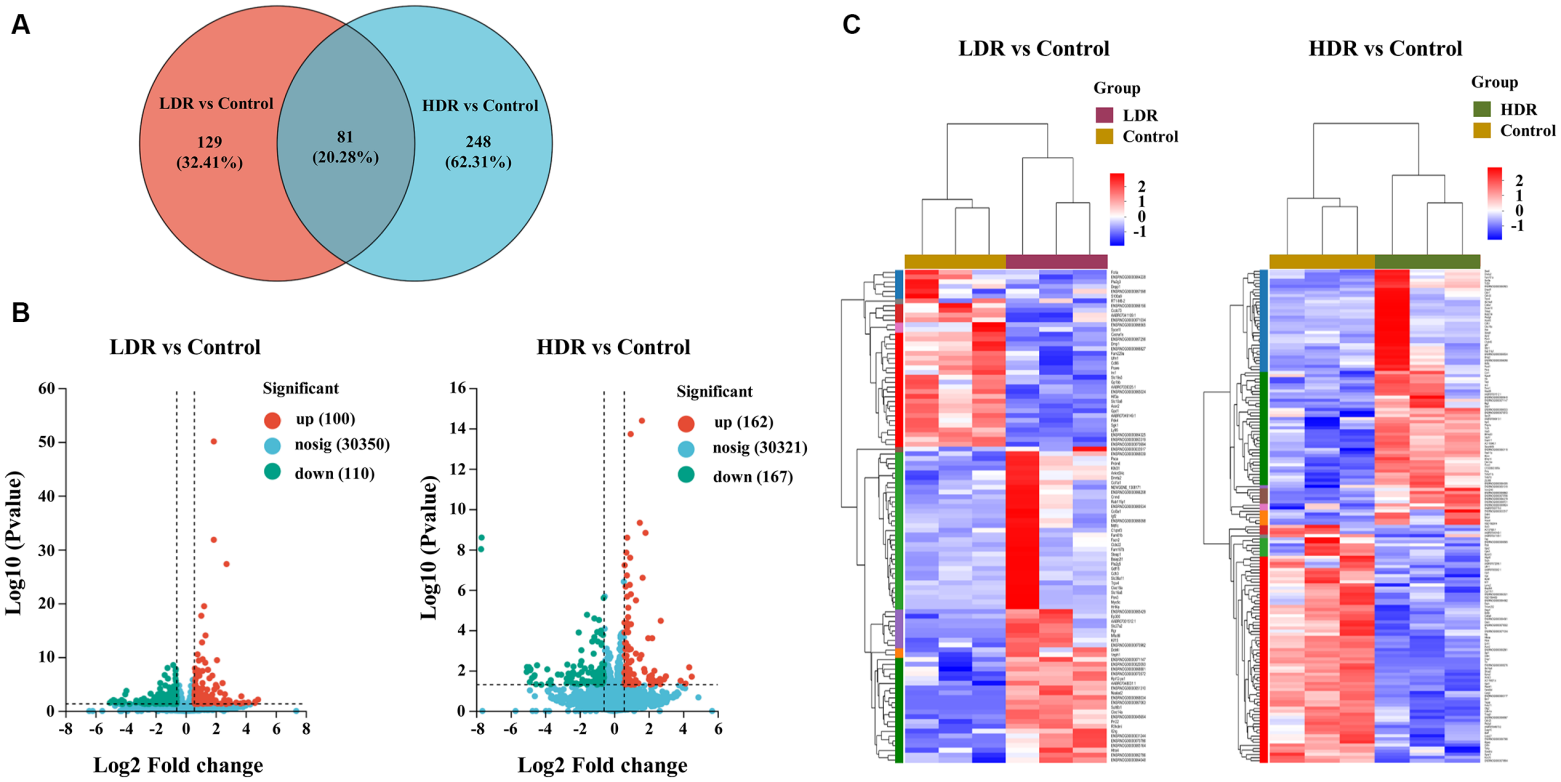
RNA sequencing demonstrates the DEGs in rat hippocampus between LDR and HDR groups. **(A)** The Venn diagram of DEGs between control and irradiated rat hippocampus. **(B)** Volcano analysis and **(C)** hierarchical clustering heatmap of DEGs in irradiated rat hippocampus, compared to controls.

**Figure 8:**
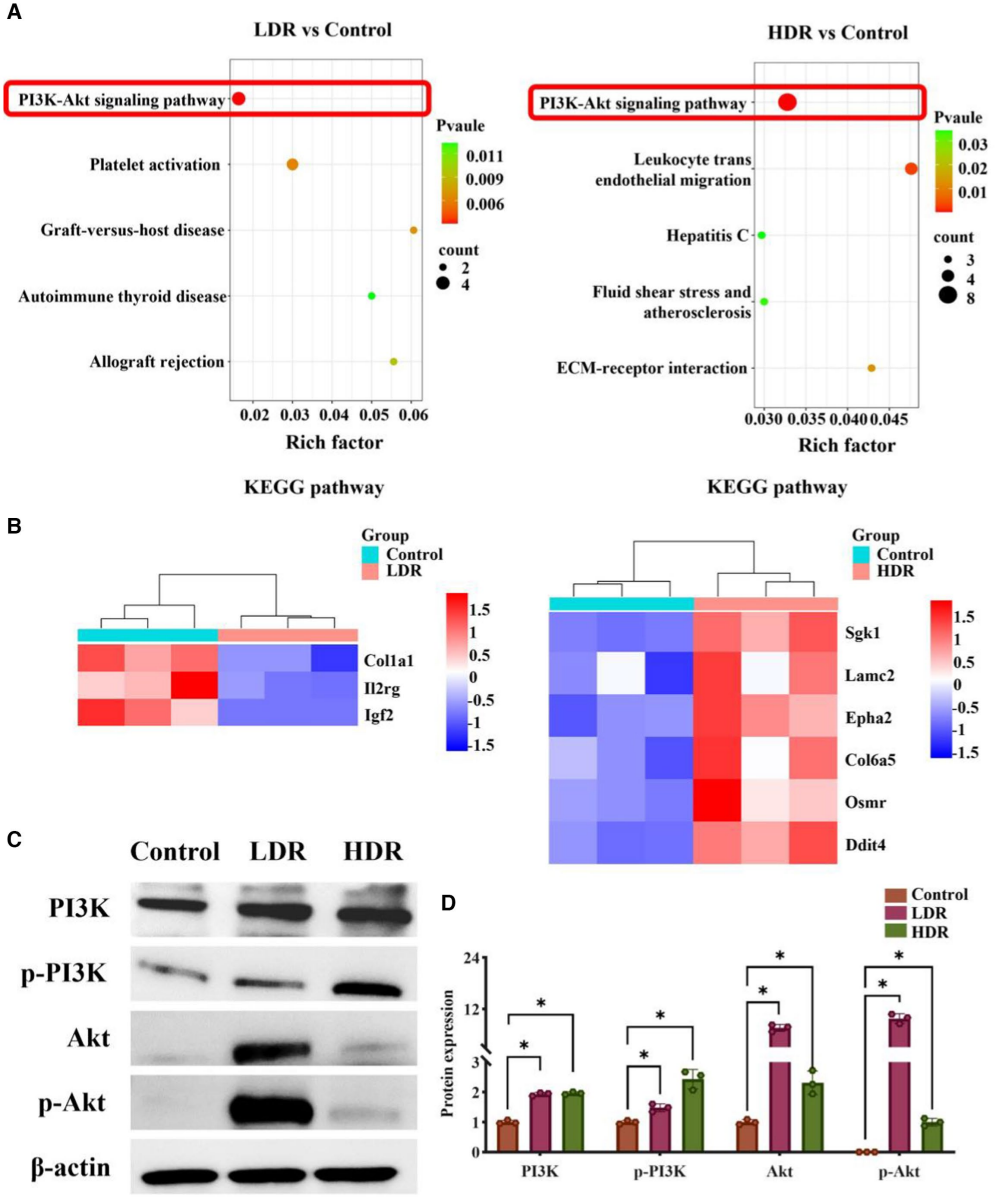
The regulation of PI3K/Akt signaling pathway in rat hippocampus in LDR and HDR groups. **(A)** KEGG enrichment analysis of differentially expressed genes (DEGs) in irradiated rat hippocampus, compared to controls. **(B)** Clustering heatmap of DEGs in PI3K/Akt signaling pathway. **(C)** The representative images of western blotting. **(D)** The relative quantification of protein expression. Data were given as mean ± SEM. One-way ANOVA test followed by LSD was used for statistical analysis (*n* = 3). *p < 0.05.

RNA-seq analysis was conducted to reveal the potential mechanism underlying the different inflammatory environments and neuronal damage in the hippocampus of the two irradiated groups. The Venn diagram revealed that 62.31% and 32.41% of differentially expressed genes (DEGs) were specifically expressed in the hippocampus of the rats in the HDR and LDR groups, respectively, and 20.28% of genes were co-expressed (Figure 7A). The volcano plot revealed 210 DEGs between the control and LDR groups and 329 DEGs between the control and HDR groups (Figure 7B). The hierarchical clustering heatmap of the transcriptomes revealed distinct gene expression clustering, suggesting significant changes in gene expression (Figure 7C). KEGG pathway function enrichment analysis revealed the highest enhancement of the DEGs related to the PI3K–Akt signaling pathway in both the LDR and HDR groups (Figure 8A). Notably, the clustering heatmap of the DEGs in the PI3K–Akt signaling pathway revealed that the DEGs in the LDR group were predominantly downregulated, whereas those in the HDR group were predominantly upregulated (Figure 8B). The activation statue of PI3K–Akt signaling pathway was further assessed by detecting the expression of PI3K and phosphorylated PI3K (p-PI3K), Akt and phosphorylated Akt (p-Akt). Western blotting results displayed that chronic LDR and HDR exposure not only upregulated the total PI3K and Akt, but also increased their respective phosphorylation in hippocampus (Figures 8C,D). Overall, these findings suggest that chronic low-dose γ-irradiation at different dose rates generate different cognitive changes possibly by regulating the PI3K–Akt signaling pathway in the hippocampus.

## 4 Discussion

Although it is universally acknowledged that low-dose γ-radiation is harmful to cognitive function, additional information on radiation-related issues remains unclear (16). Radiation dose, irradiation method, radiation type, and dose rate are the factors affecting radiation-induced biological effects (17). For example, recently, a study revealed that acute γ-irradiation at 10 Gy/s can cause more serious cognitive impairment than that at 100 Gy/s, emphasizing the importance of dose rate (18). Therefore, the correlation between dose rate and the extent of cognitive impairment may not be as simple as previously assumed. In the present study, we observed that chronic low-dose γ-irradiation at LDR induces cognitive impairment in rats as early as 2 weeks after irradiation; this impairment persisted throughout the research period. In contrast, the cognitive dysfunction induced by chronic low-dose γ-radiation at HDR lasted for 2 months but was recovered at the 4-month time point. Collectively, these results suggest that chronic low-dose γ-radiation at LDR results in more severe cognitive impairment than that at HDR. In addition, our findings suggest that the relationship between dose rate and LDIR-induced cognitive changes may be more complicated. Furthermore, according to our present result, it seemed that, even at low-dose irradiation, the adaptive response varied with dose-rates. In details, cognitive impairment caused by chronic low-dose radiation at HDR would recover over time, while LDR did not, meaning that an adaptive response only occurred in the former. One possible reason for this phenomenon is that LDR-induced insufficient brain damage fails to arouse adaptive response.

Previous studies have revealed that radiation induces cognitive impairment without altering the brain morphology (9, 18, 19). In the present study, no apparent brain tissue lesions were observed however, glucose metabolism significantly increased in the brains of the irradiated rats, indicating the occurrence of inflammation in the brain; this is consistent with the findings of a recent study (20). Low-dose γ-irradiation destroys the synaptic plasticity and BBB integrity; this may correlate to brain inflammation (21). Nevertheless, additional studies on the mechanism underlying synaptic function and BBB injuries are warranted.

Considering that the hippocampus is a radiation-sensitive tissue in the brain and the target of radiation-induced cognitive impairment (22), we further elucidated the structural and functional changes in this region. Past studies have shown that exposure to radiation at LDR can induce brain injury via the activation of microglia or/and astrocytes (14, 23). More importantly, radiation-activated astrocytes and microglia have ability of causing cognitive changes (24–27). We observed that the number of inflammatory M1-like microglia and neurotoxic A1-like astrocytes significantly increased in the hippocampus of the rats in the LDR group; however, only M1-type microglia were activated in the HDR group. These results suggest that radiation at LDR and HDR induces different inflammatory responses in the hippocampus of the irradiated rats. Furthermore, the number of hippocampal neurons significantly decreased in the rats in the HDR group, indicating neuronal damage; in contrast, the rats in the LDR group did not exhibit this phenomenon. Taken together, radiation at LDR may not directly cause neuronal damage; however, it may promote the activation of microglia and astrocytes, further inducing chronic inflammation in the hippocampus. On the other hand, radiation at HDR not only leads to inflammation, which is less severe than that in the LDR group, but also directly damages hippocampal neurons. Combined with cognitive changes, we hypothesize that the chronic inflammation induced by chronic γ-radiation at LDR may induce more severe cognitive impairment than the direct neuronal damage induced by radiation at HDR.

The PI3K–Akt signaling pathway is essential in post-injury repair in various neurodegenerative diseases (28, 29). Previous studies have demonstrated that PI3K–Akt signaling pathway can regulate inflammatory responses, particularly in the context of brain inflammation, by interacting with multiple signaling pathways (30–34). In the present study, KEGG pathway enrichment analysis revealed that the DEGs were mainly enriched in the PI3K–Akt signaling pathway, in which the DEGs was upregulated in the hippocampus of the rats exposed to low-dose γ-radiation at HDR, but downregulated in the rats exposed to radiation at LDR. Furthermore, our western blotting results confirmed that chronic low-dose irradiation at LDR and HDR both could significantly increase the expression of core proteins (such as PI3K, p-PI3K, Akt, p-Akt) of PI3K–Akt signaling pathway in the hippocampus of rats, meaning the activation of the above pathway. It appears to be dose rate-dependent when considering chronic low-dose radiation-induced activation of PI3K–Akt signaling pathway, which also explain the reasons for the different inflammatory environments and neuronal damage in the hippocampus of irradiated rats. Based on the above inference, PI3K–Akt signaling pathway is crucial in regulating and facilitating the recovery of cognitive impairment in HDR-irradiated rats, providing potential new targets for the treatment of radiation-caused cognitive alternation.

In conclusion, our study revealed that compared with chronic low-dose γ irradiation at HDR, that at LDR causes more serious or persistent cognitive impairment, possibly involving the activation of chronic inflammatory responses in the hippocampus. Furthermore, the PI3K–Akt signaling pathway in the hippocampus is differentially regulated by chronic low-dose γ irradiation at different dose rates, providing a potential therapeutic target for cognitive impairment.

## Data availability statement

The original contributions presented in the study are included in the article/supplementary materials, further inquiries can be directed to the corresponding authors.

## Ethics statement

The animal studies were approved by Animal Experiment Ethics Committee of the Navy Medical Center. The studies were conducted in accordance with the local legislation and institutional requirements. Written informed consent was obtained from the owners for the participation of their animals in this study.

## Author contributions

TM: Data curation, Formal analysis, Investigation, Methodology, Writing – original draft. KL: Data curation, Formal analysis, Methodology, Writing – original draft. WS: Methodology, Writing – original draft. XLi: Methodology, Software, Visualization, Writing – original draft. QL: Methodology, Software, Writing – original draft. YP: Methodology, Writing – original draft. MW: Methodology, Writing – original draft. XLu: Methodology, Software, Writing – original draft. JF: Methodology, Visualization, Writing – original draft. HW: Conceptualization, Project administration, Resources, Supervision, Writing – review & editing. TW: Conceptualization, Formal analysis, Supervision, Writing – review & editing. CZ: Conceptualization, Formal analysis, Funding acquisition, Project administration, Resources, Supervision, Writing – review & editing.
