# Supplementary figures and images for: Low-dose-rate induces more severe cognitive impairment than high-dose-rate in rats exposed to chronic low-dose γ-radiation
Source: Front Public Health. 2024 May 22;12:1387330. doi: 10.3389/fpubh.2024.1387330 (PMC11150688; doi:10.3389/fpubh.2024.1387330)

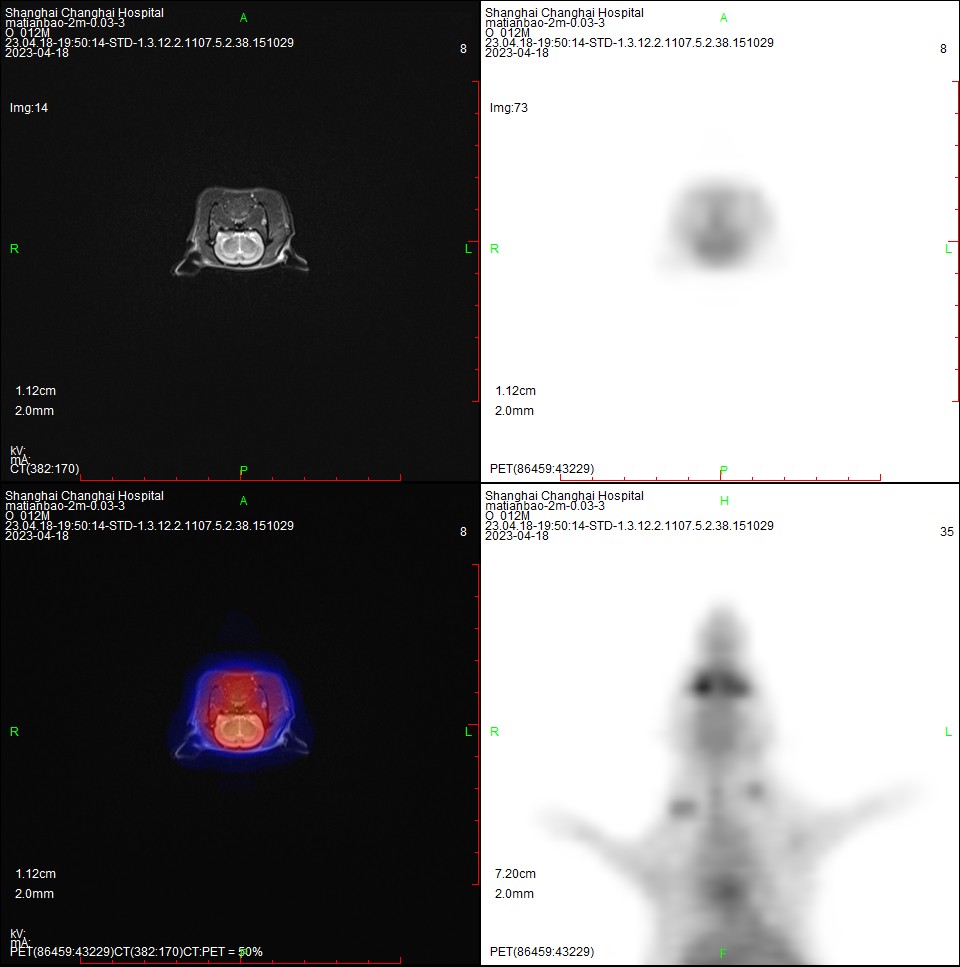

Supplement: Supplementary file 1 [file Data_Sheet_1.zip › Raw data-1/Figure 2-raw data/original images-PETMR/control-2m.jpg]

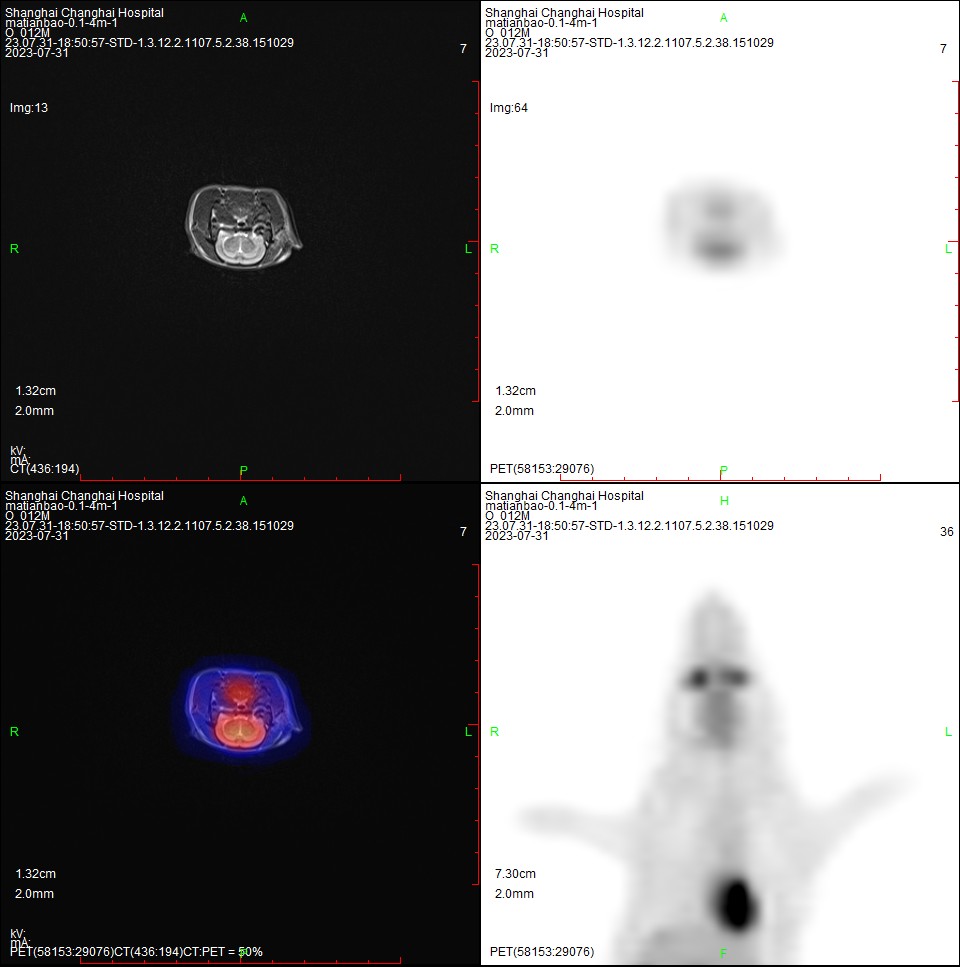

Supplement: Supplementary file 1 [file Data_Sheet_1.zip › Raw data-1/Figure 2-raw data/original images-PETMR/control-2w.jpg]

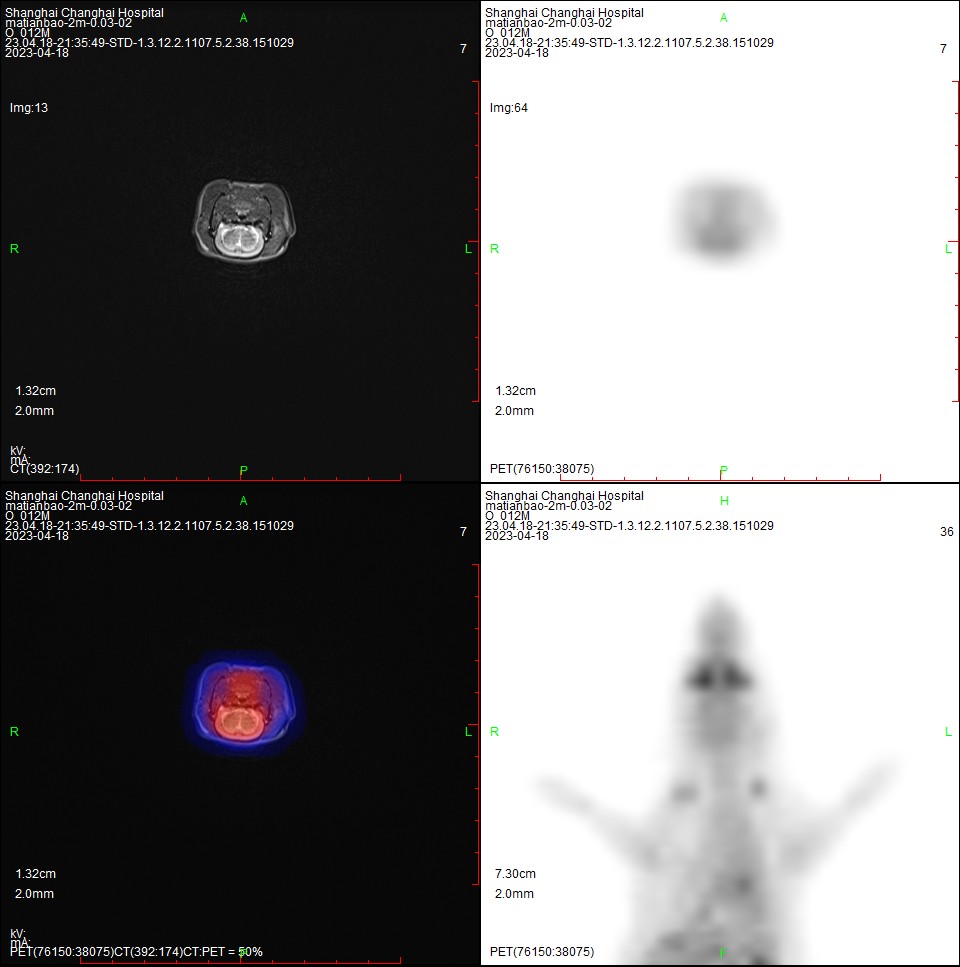

Supplement: Supplementary file 1 [file Data_Sheet_1.zip › Raw data-1/Figure 2-raw data/original images-PETMR/control-4m.jpg]

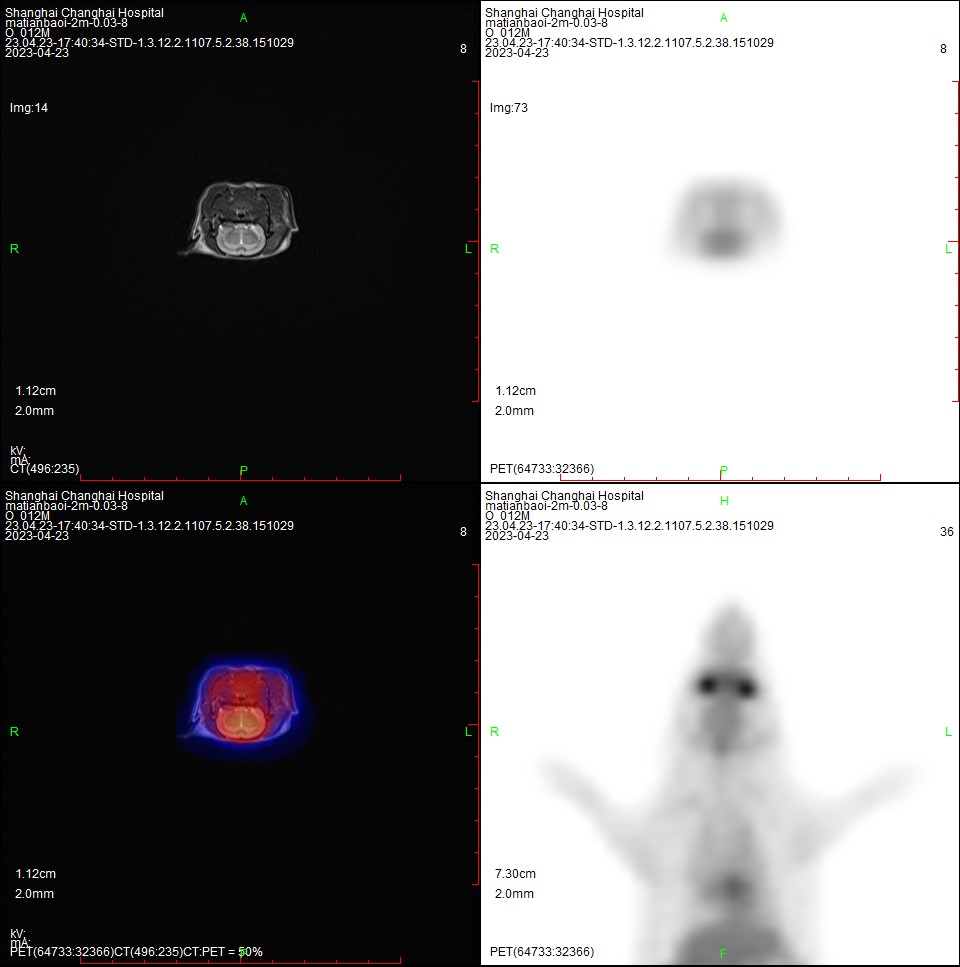

Supplement: Supplementary file 1 [file Data_Sheet_1.zip › Raw data-1/Figure 2-raw data/original images-PETMR/HDR-2m.jpg]

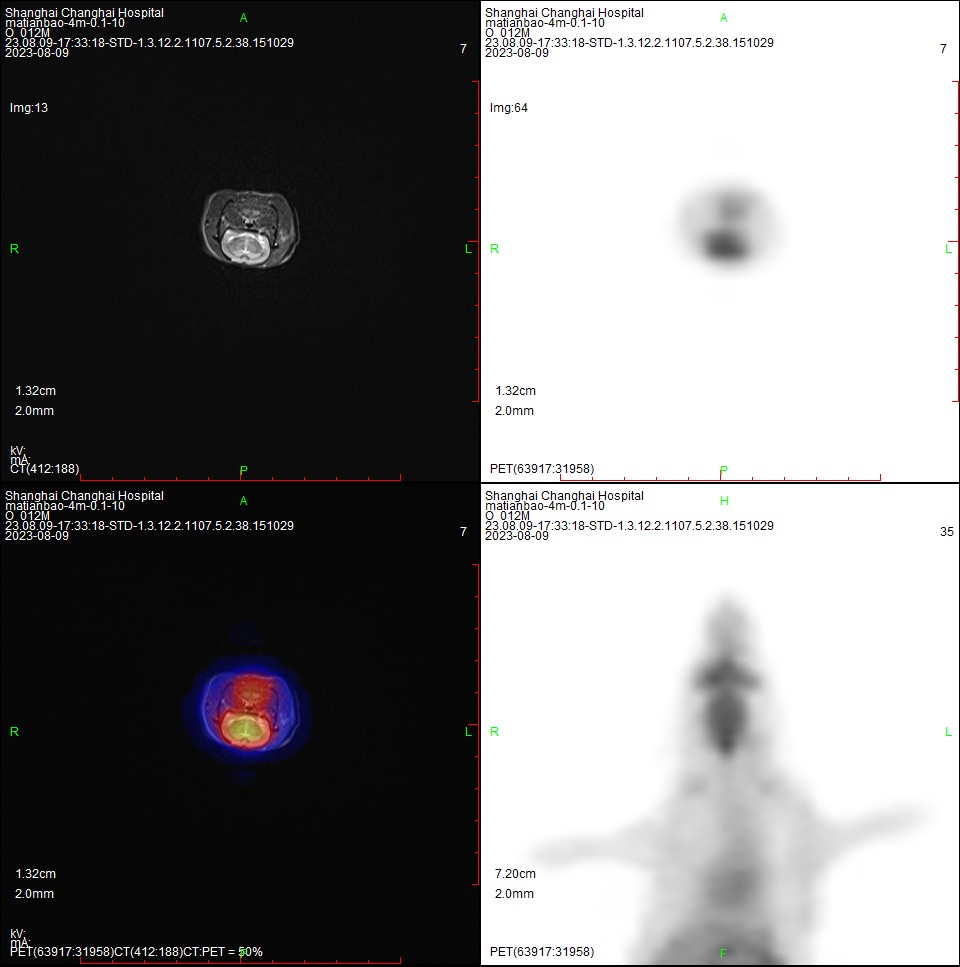

Supplement: Supplementary file 1 [file Data_Sheet_1.zip › Raw data-1/Figure 2-raw data/original images-PETMR/HDR-2w.jpg]

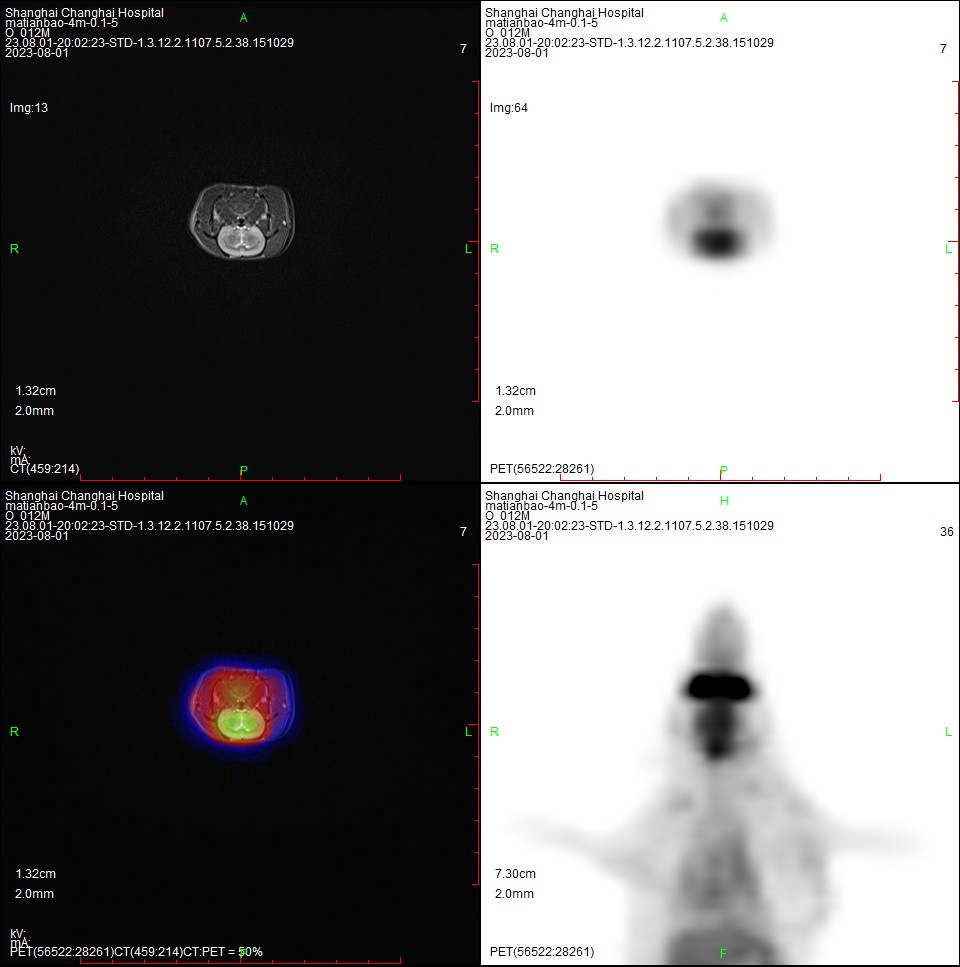

Supplement: Supplementary file 1 [file Data_Sheet_1.zip › Raw data-1/Figure 2-raw data/original images-PETMR/HDR-4m.jpg]

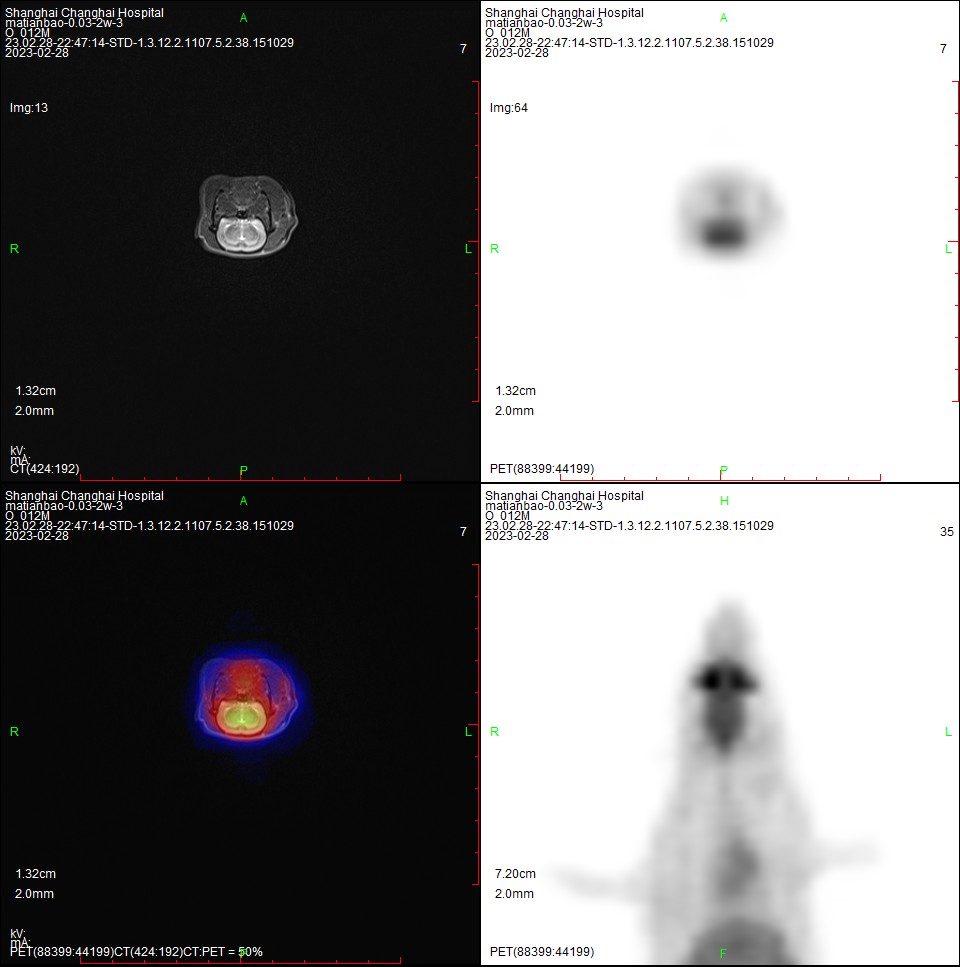

Supplement: Supplementary file 1 [file Data_Sheet_1.zip › Raw data-1/Figure 2-raw data/original images-PETMR/LDR-2m.jpg]

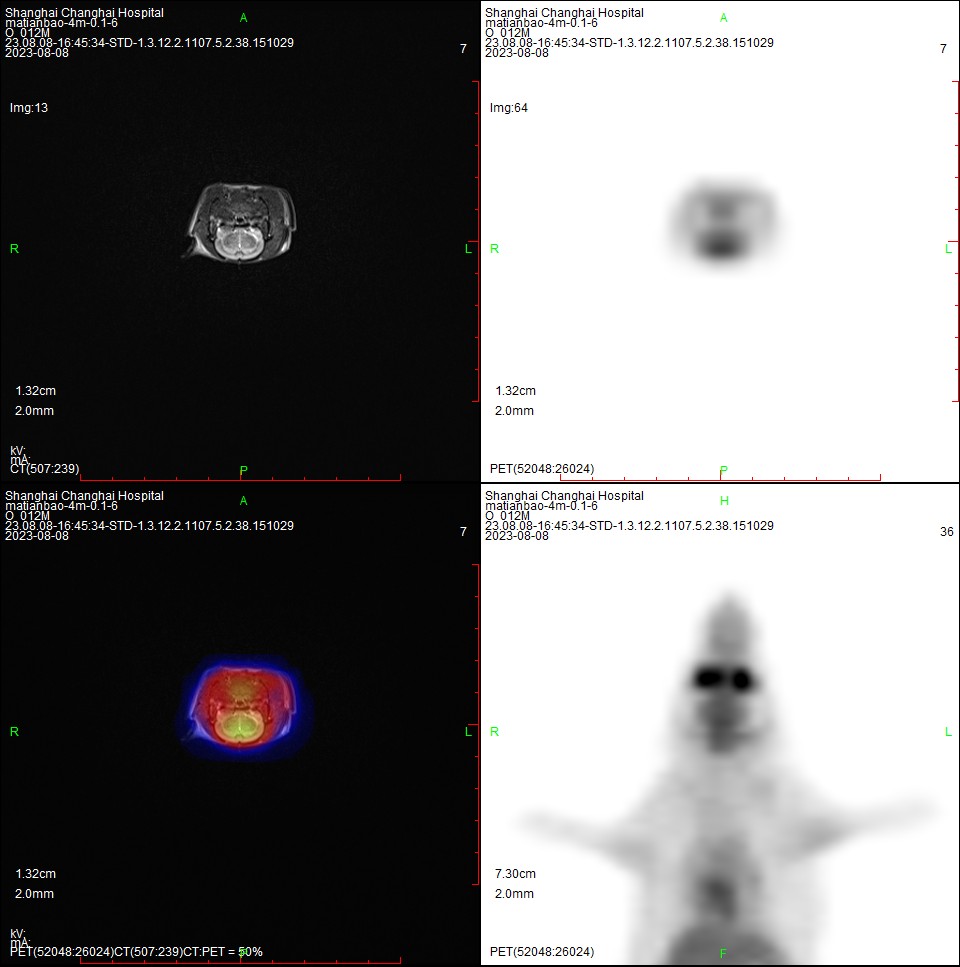

Supplement: Supplementary file 1 [file Data_Sheet_1.zip › Raw data-1/Figure 2-raw data/original images-PETMR/LDR-2w.jpg]

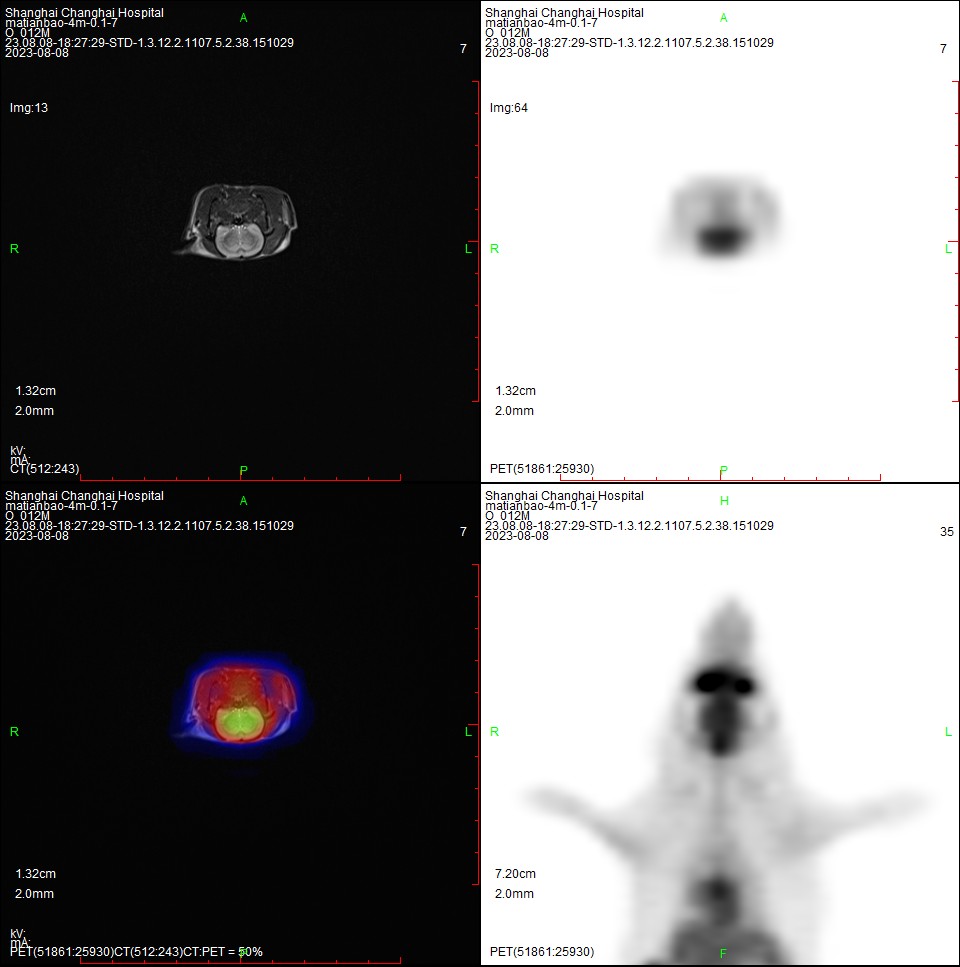

Supplement: Supplementary file 1 [file Data_Sheet_1.zip › Raw data-1/Figure 2-raw data/original images-PETMR/LDR-4m.jpg]

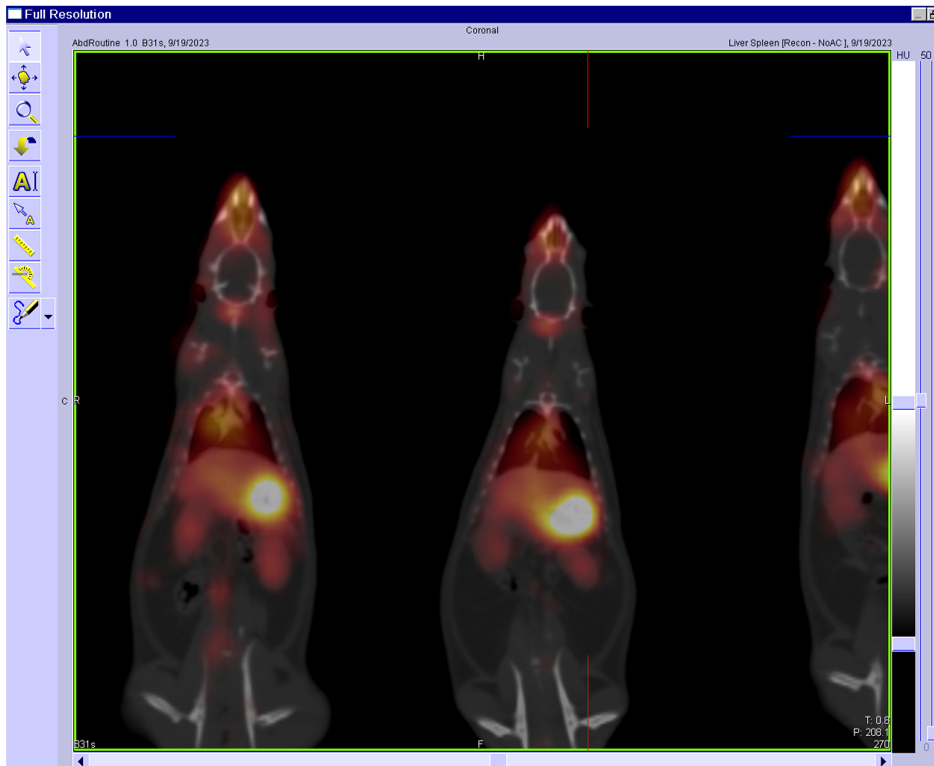

Supplement: Supplementary file 1 [file Data_Sheet_1.zip › Raw data-1/Figure 3-raw data/original images-SPECTCT/control-1 2.png]

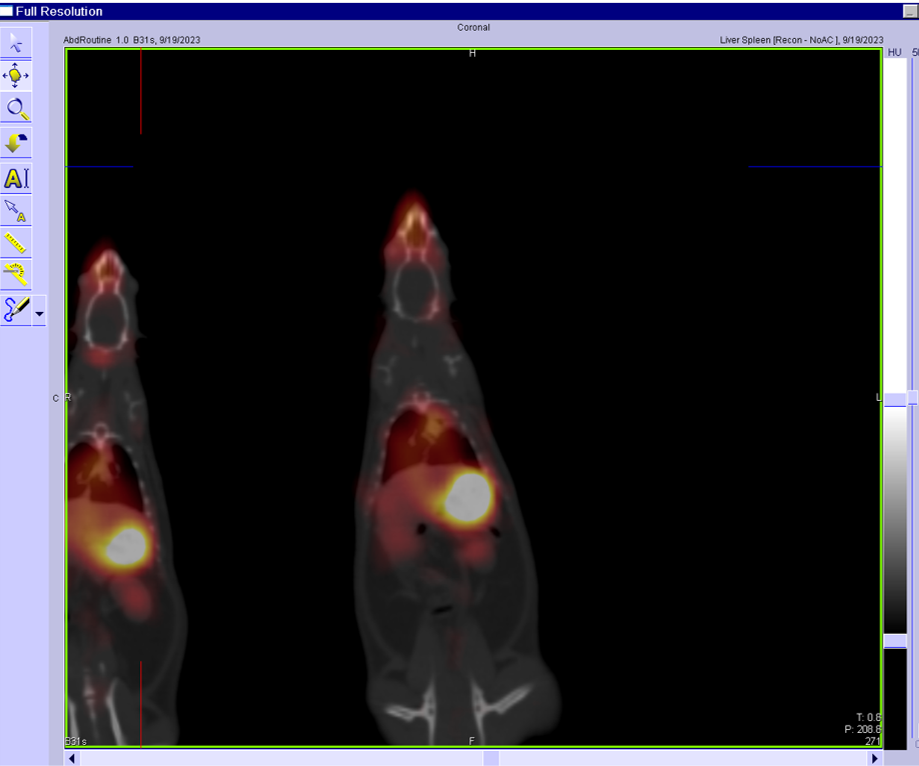

Supplement: Supplementary file 1 [file Data_Sheet_1.zip › Raw data-1/Figure 3-raw data/original images-SPECTCT/control-3.png]

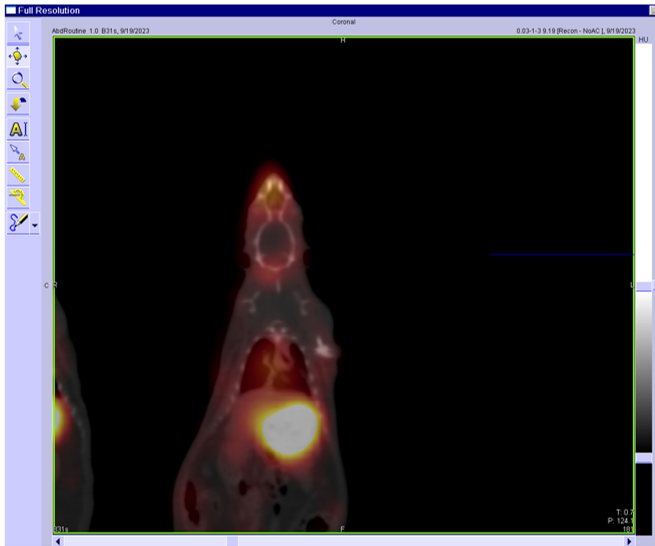

Supplement: Supplementary file 1 [file Data_Sheet_1.zip › Raw data-1/Figure 3-raw data/original images-SPECTCT/HDR-1.png]

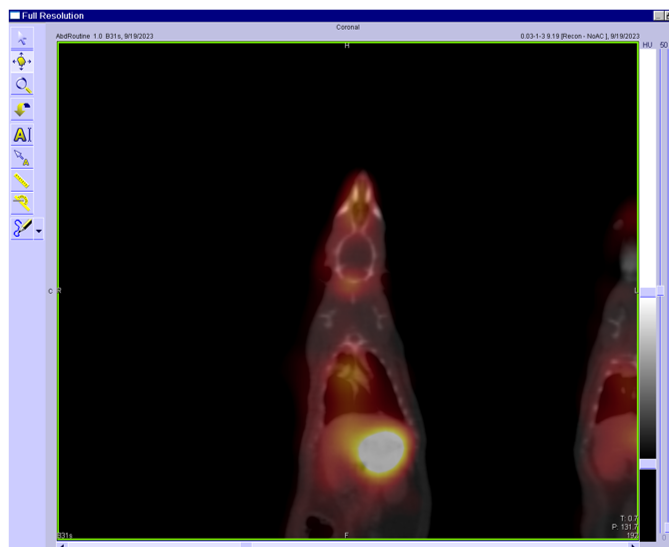

Supplement: Supplementary file 1 [file Data_Sheet_1.zip › Raw data-1/Figure 3-raw data/original images-SPECTCT/HDR-2.png]

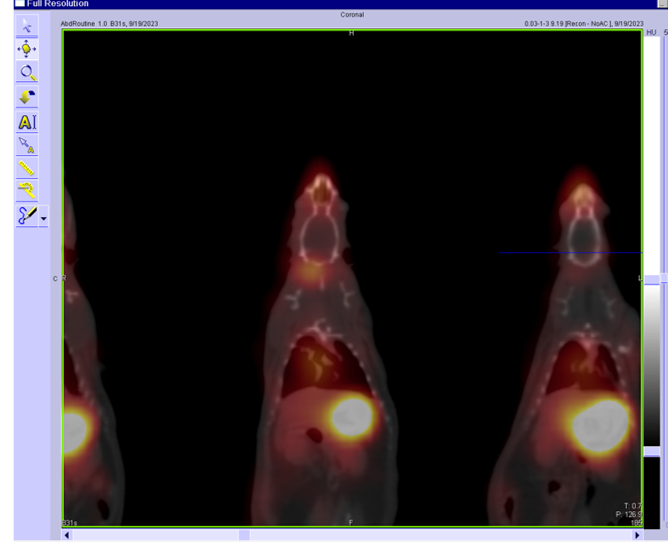

Supplement: Supplementary file 1 [file Data_Sheet_1.zip › Raw data-1/Figure 3-raw data/original images-SPECTCT/HDR-3.png]

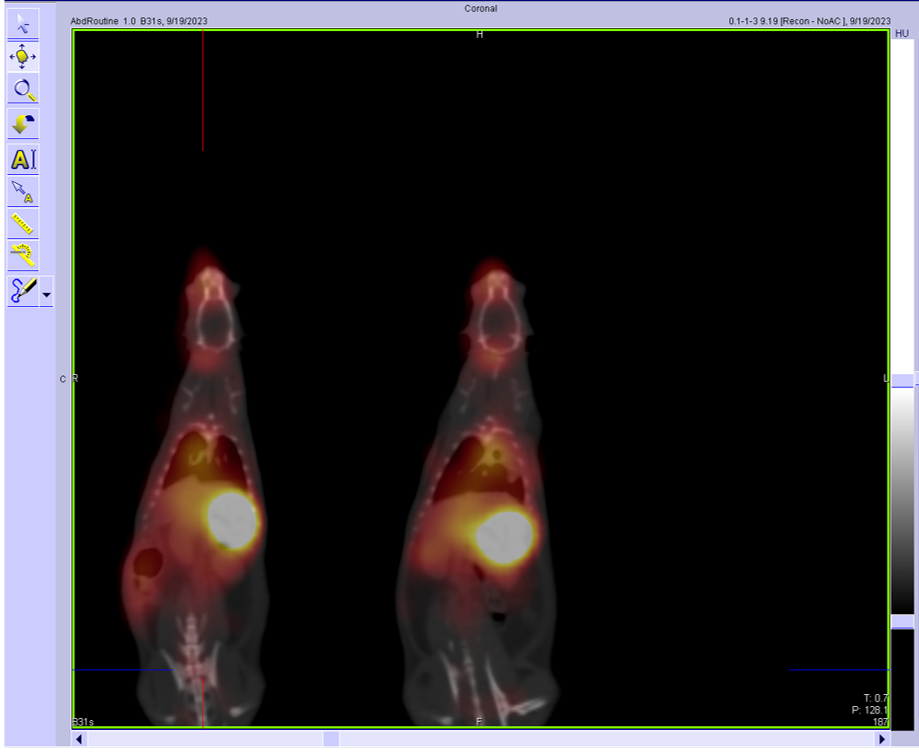

Supplement: Supplementary file 1 [file Data_Sheet_1.zip › Raw data-1/Figure 3-raw data/original images-SPECTCT/LDR-1.png]

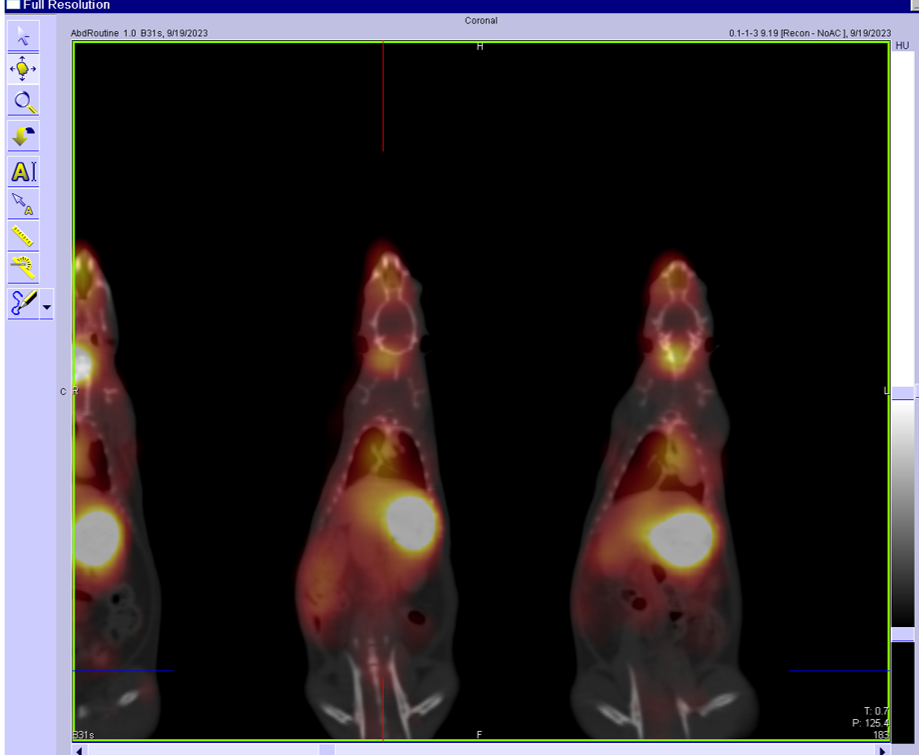

Supplement: Supplementary file 1 [file Data_Sheet_1.zip › Raw data-1/Figure 3-raw data/original images-SPECTCT/LDR-2.png]

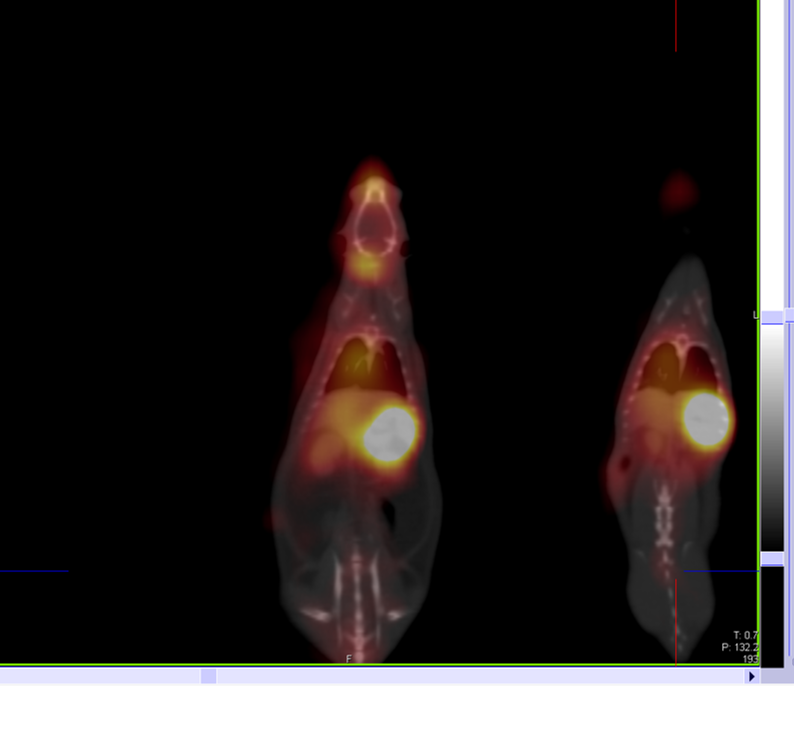

Supplement: Supplementary file 1 [file Data_Sheet_1.zip › Raw data-1/Figure 3-raw data/original images-SPECTCT/LDR-3.png]

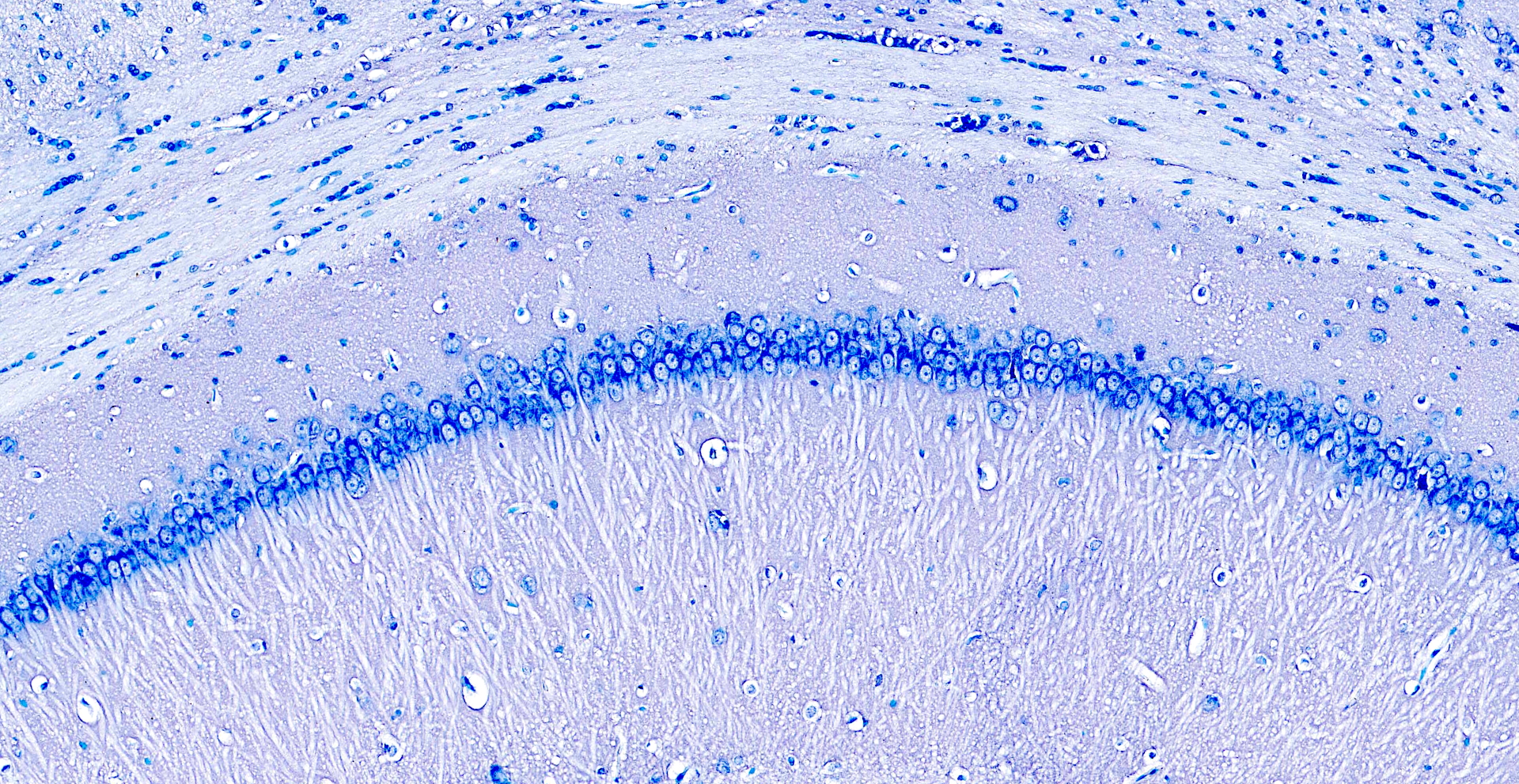

Supplement: Supplementary file 1 [file Data_Sheet_1.zip › Raw data-1/Figure 4-raw data/original images-Nissl/control-Nissl CA1.jpg]

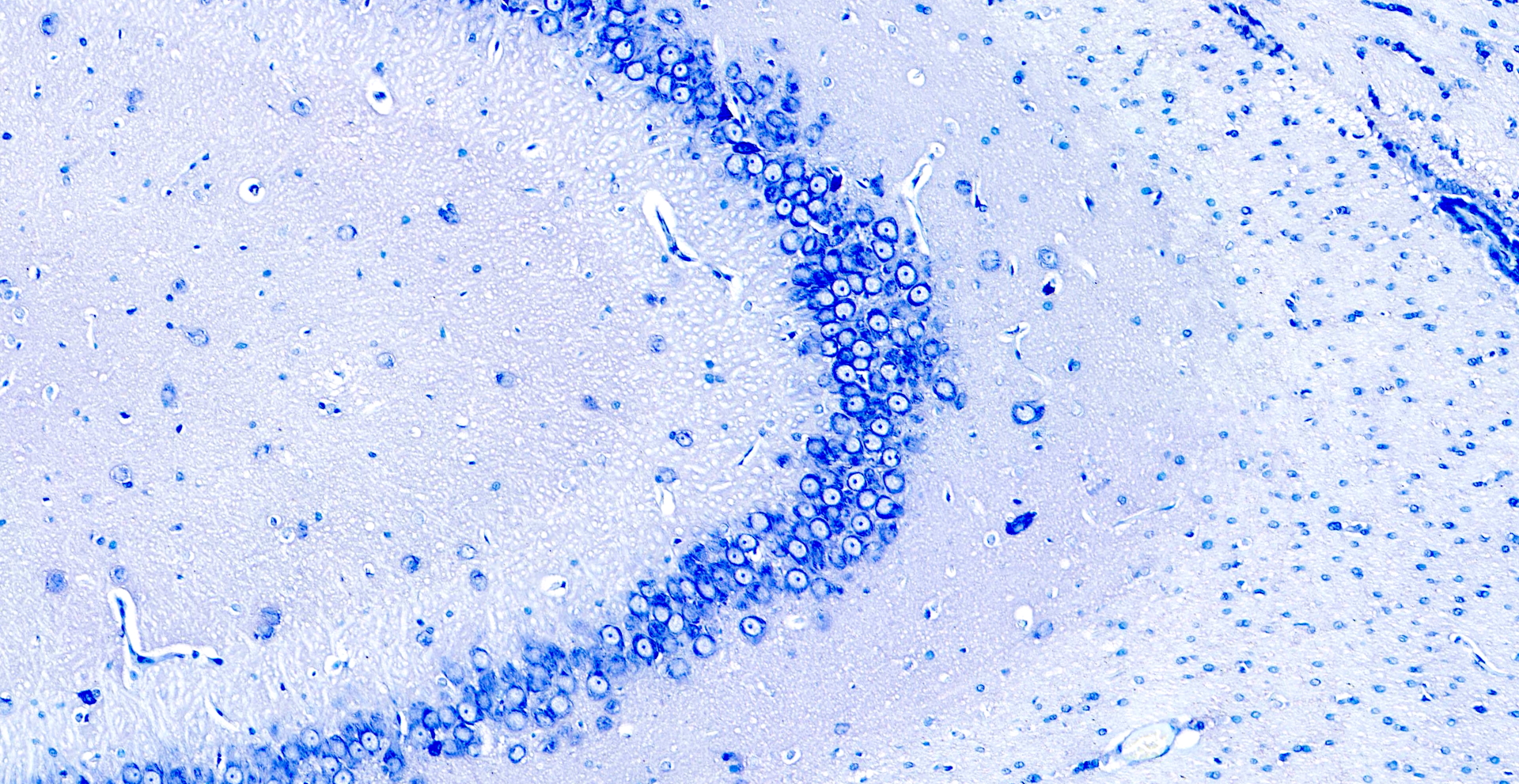

Supplement: Supplementary file 1 [file Data_Sheet_1.zip › Raw data-1/Figure 4-raw data/original images-Nissl/control-Nissl CA3a.jpg]

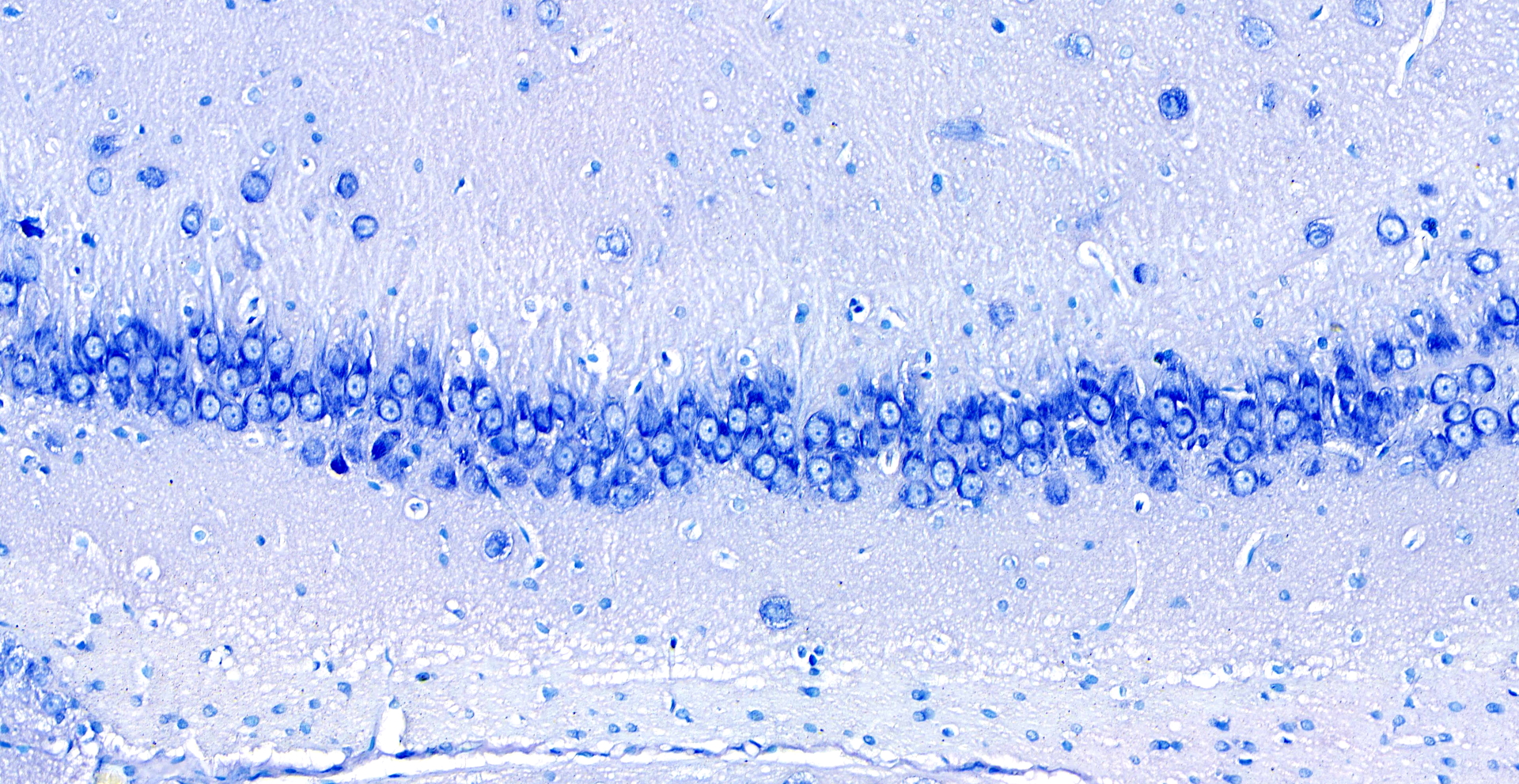

Supplement: Supplementary file 1 [file Data_Sheet_1.zip › Raw data-1/Figure 4-raw data/original images-Nissl/control-Nissl CA3b.jpg]

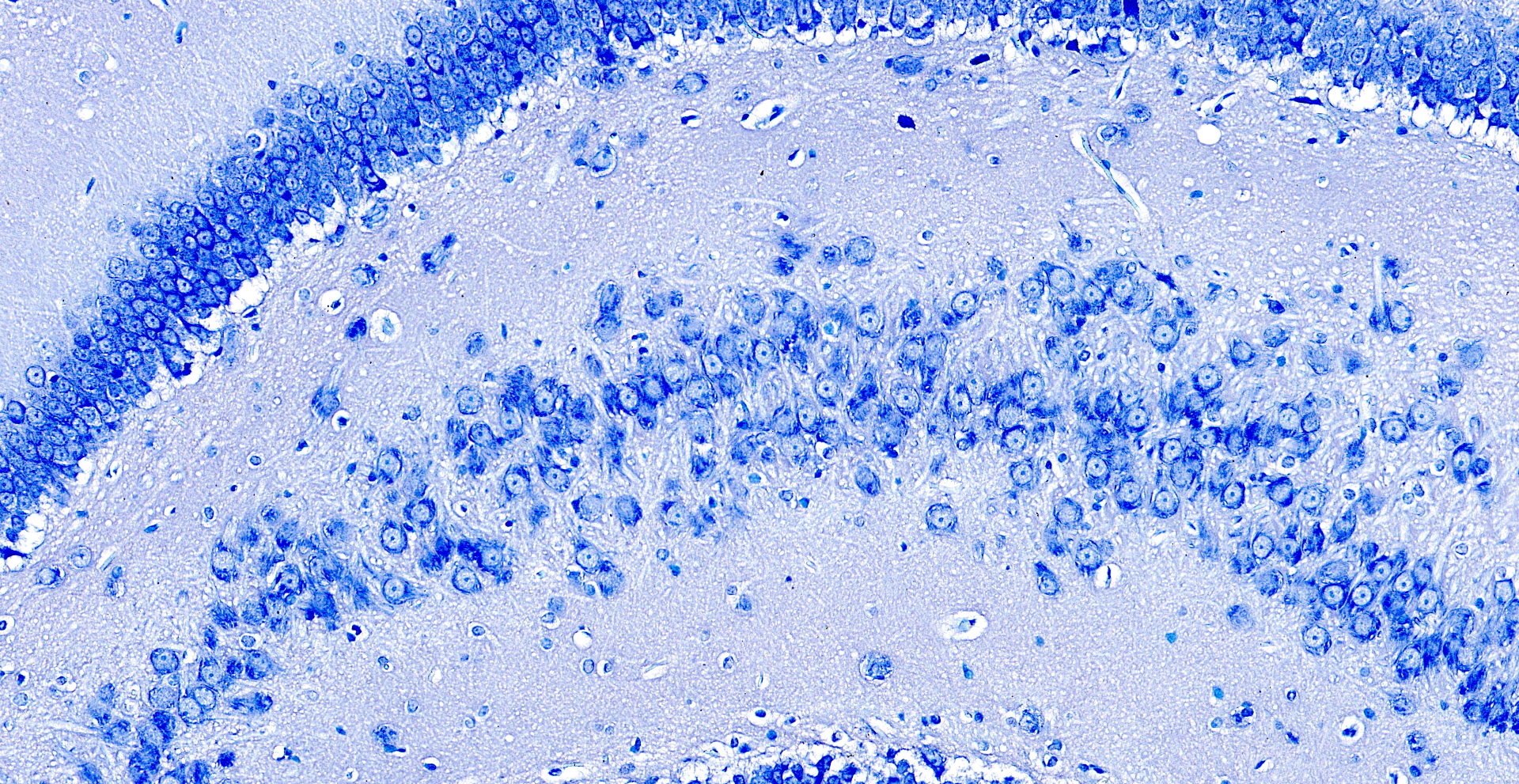

Supplement: Supplementary file 1 [file Data_Sheet_1.zip › Raw data-1/Figure 4-raw data/original images-Nissl/control-Nissl CA3c.jpg]

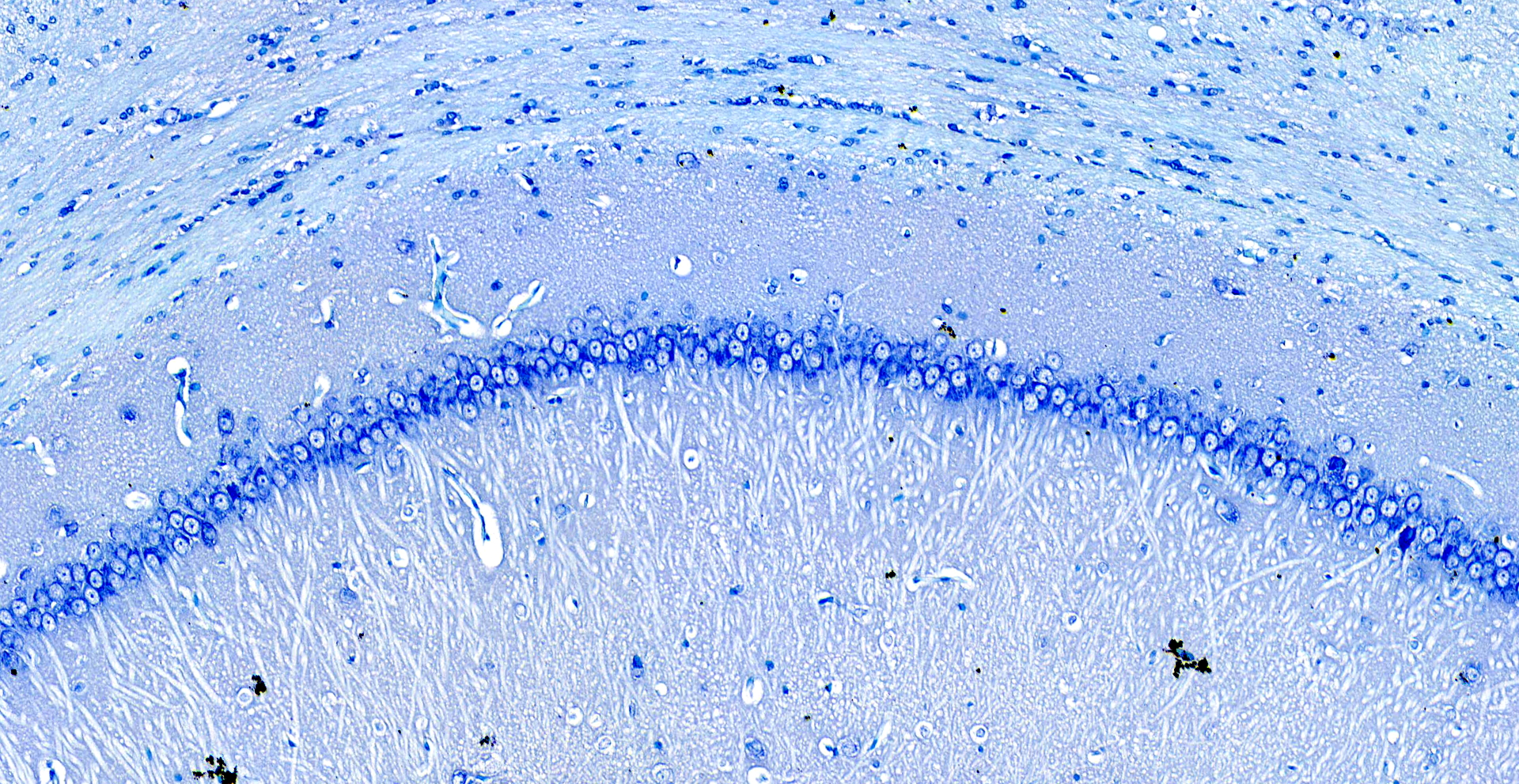

Supplement: Supplementary file 1 [file Data_Sheet_1.zip › Raw data-1/Figure 4-raw data/original images-Nissl/HDR-Nissl CA1.jpg]

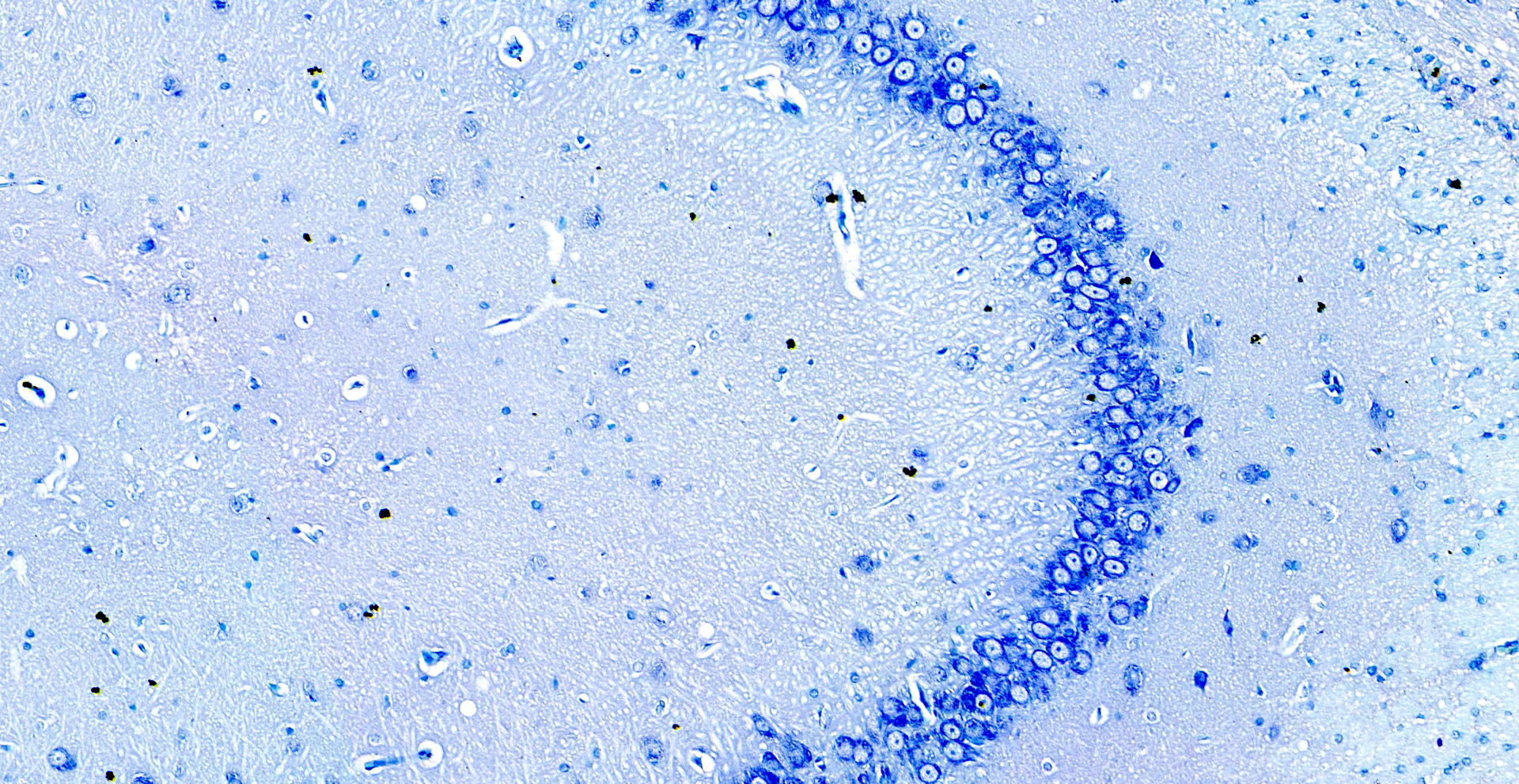

Supplement: Supplementary file 1 [file Data_Sheet_1.zip › Raw data-1/Figure 4-raw data/original images-Nissl/HDR-Nissl CA3a.jpg]

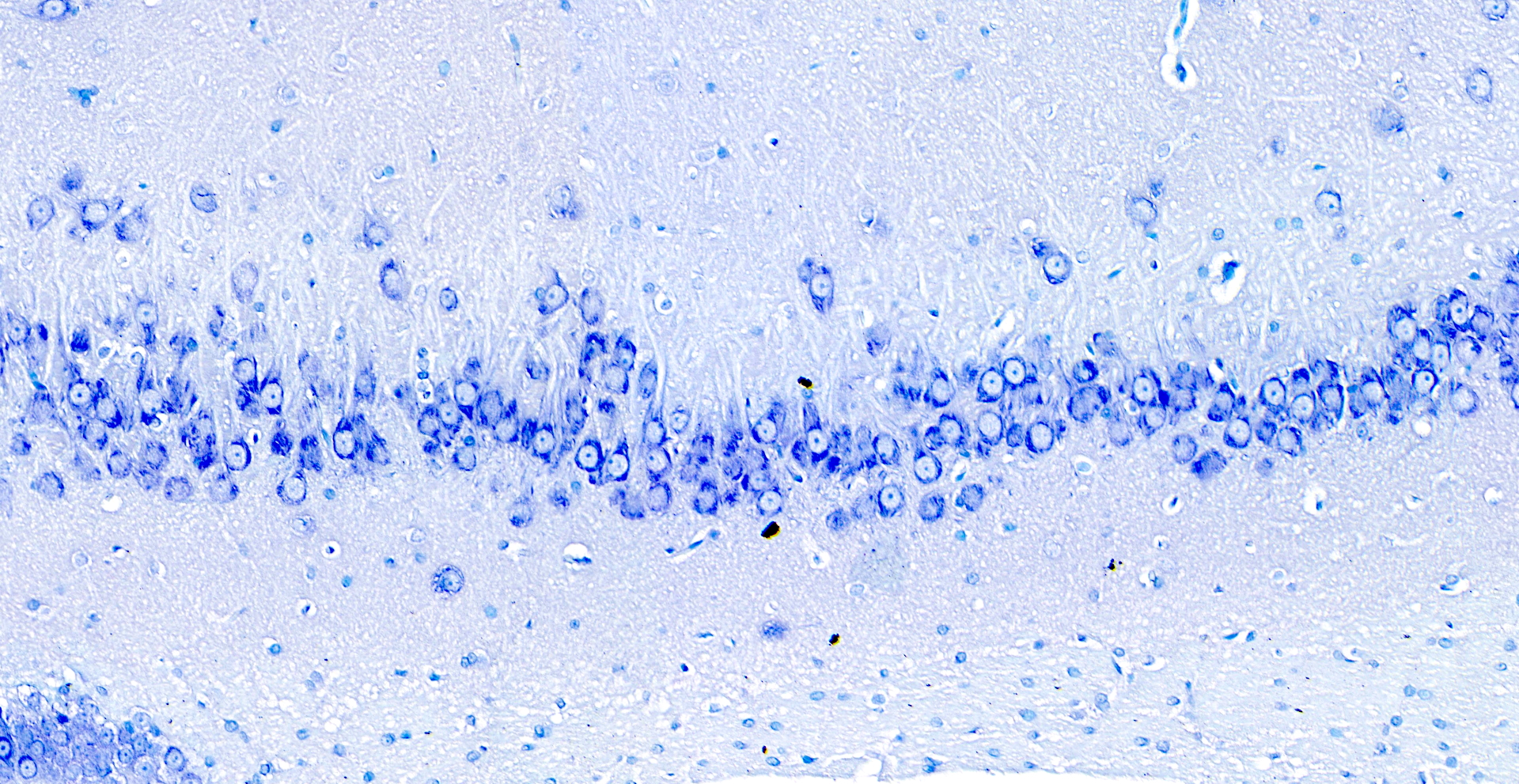

Supplement: Supplementary file 1 [file Data_Sheet_1.zip › Raw data-1/Figure 4-raw data/original images-Nissl/HDR-Nissl CA3b.jpg]

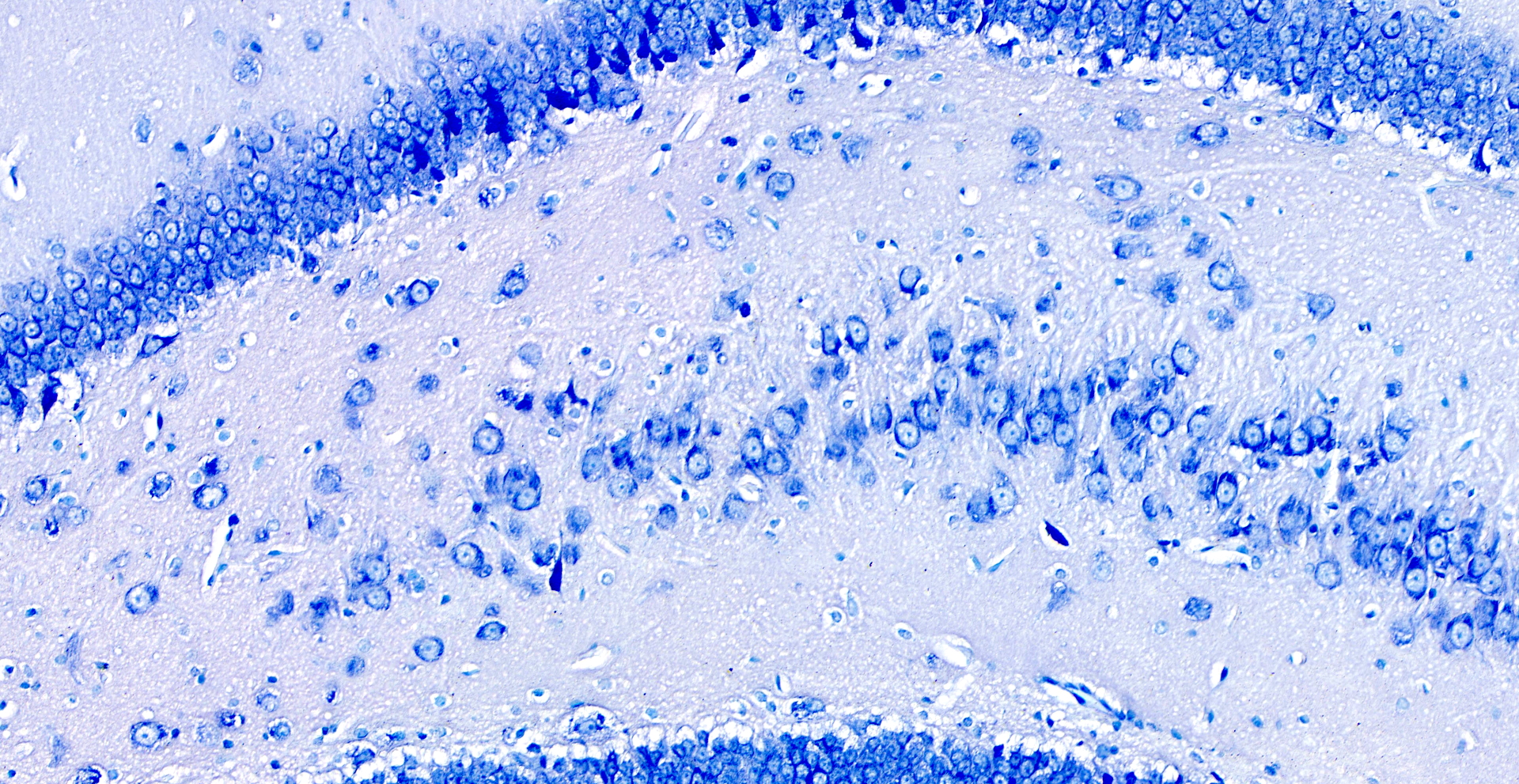

Supplement: Supplementary file 1 [file Data_Sheet_1.zip › Raw data-1/Figure 4-raw data/original images-Nissl/HDR-Nissl CA3c.jpg]

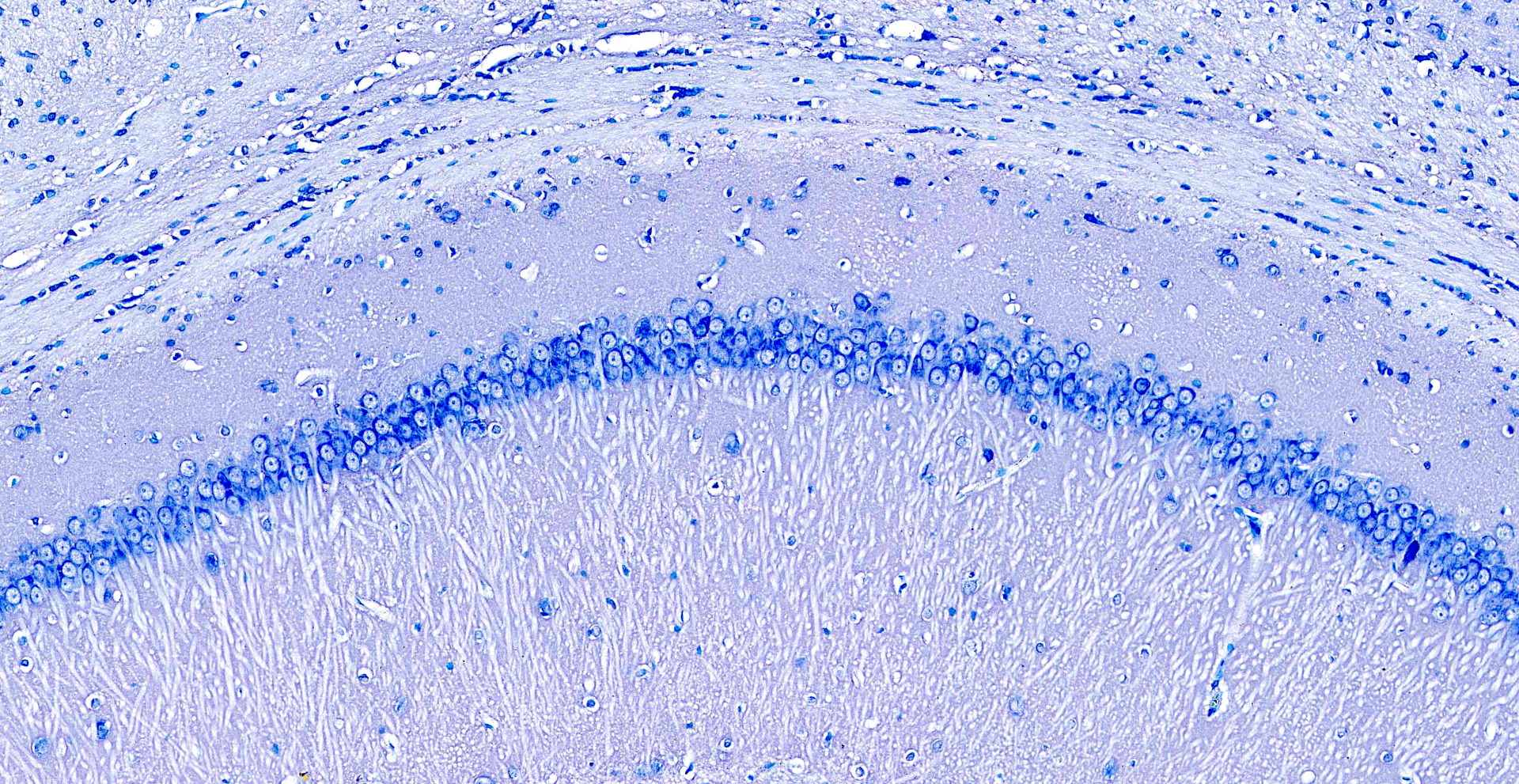

Supplement: Supplementary file 1 [file Data_Sheet_1.zip › Raw data-1/Figure 4-raw data/original images-Nissl/LDR-Nissl CA1.jpg]

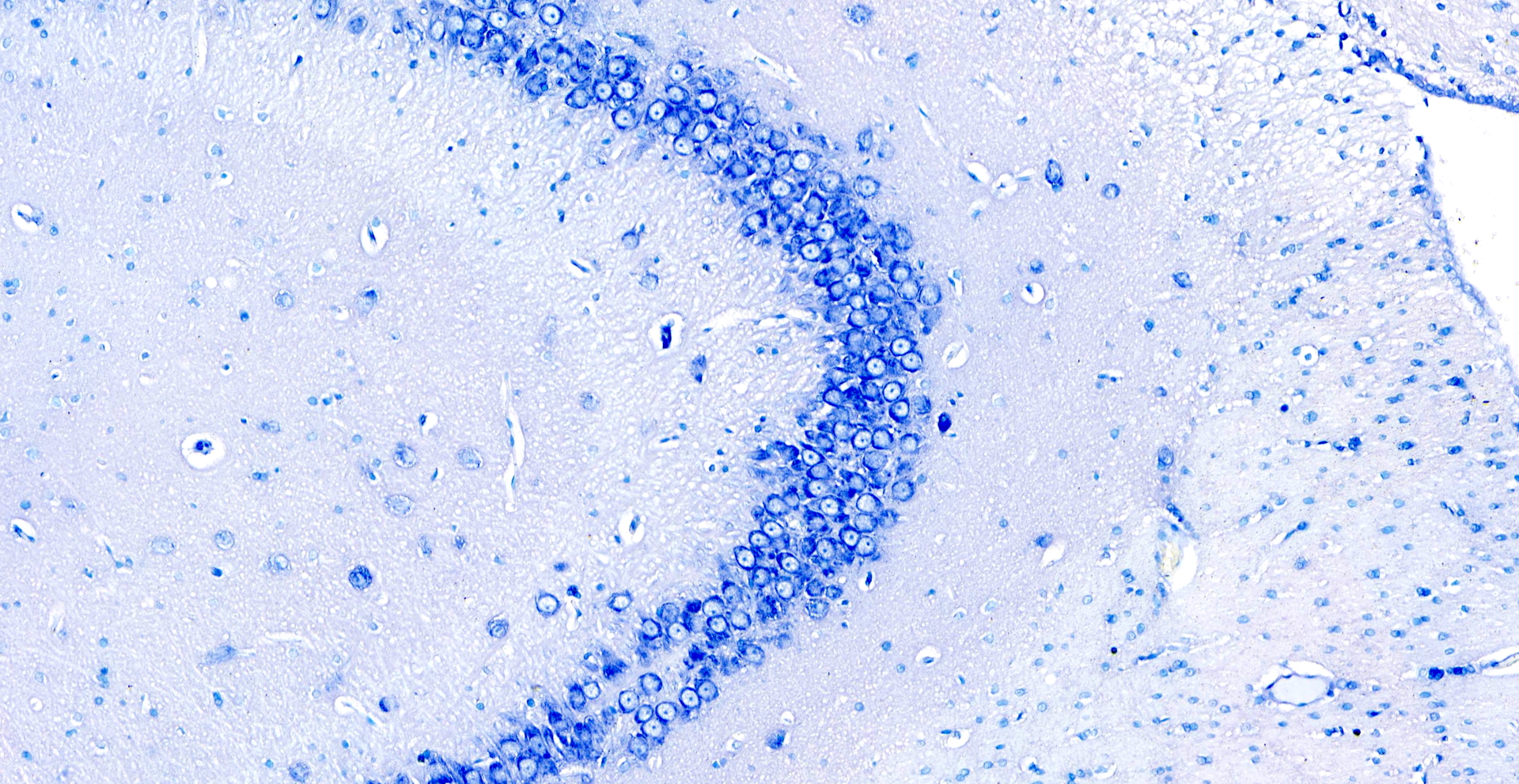

Supplement: Supplementary file 1 [file Data_Sheet_1.zip › Raw data-1/Figure 4-raw data/original images-Nissl/LDR-Nissl CA3a.jpg]

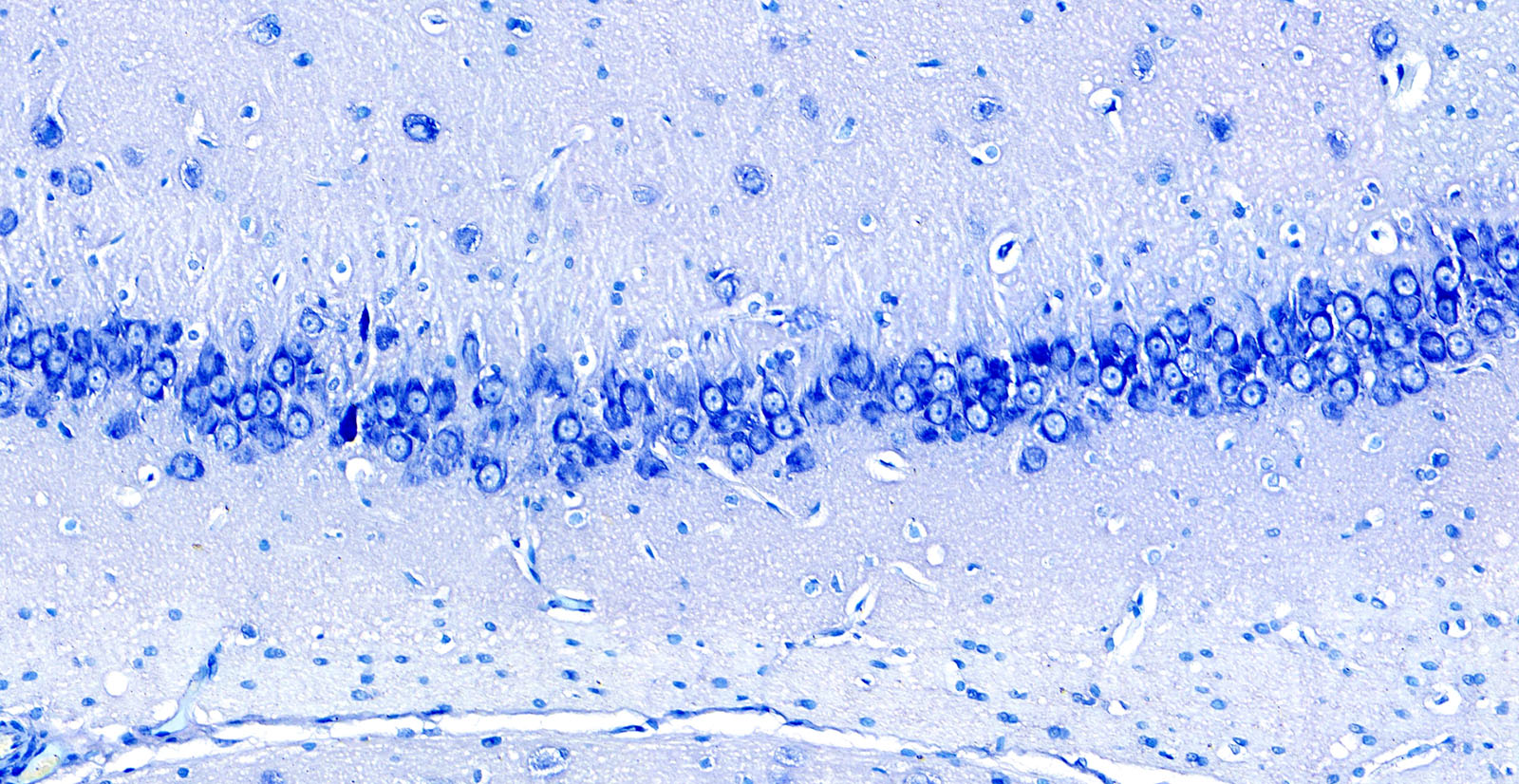

Supplement: Supplementary file 1 [file Data_Sheet_1.zip › Raw data-1/Figure 4-raw data/original images-Nissl/LDR-Nissl CA3b.jpg]

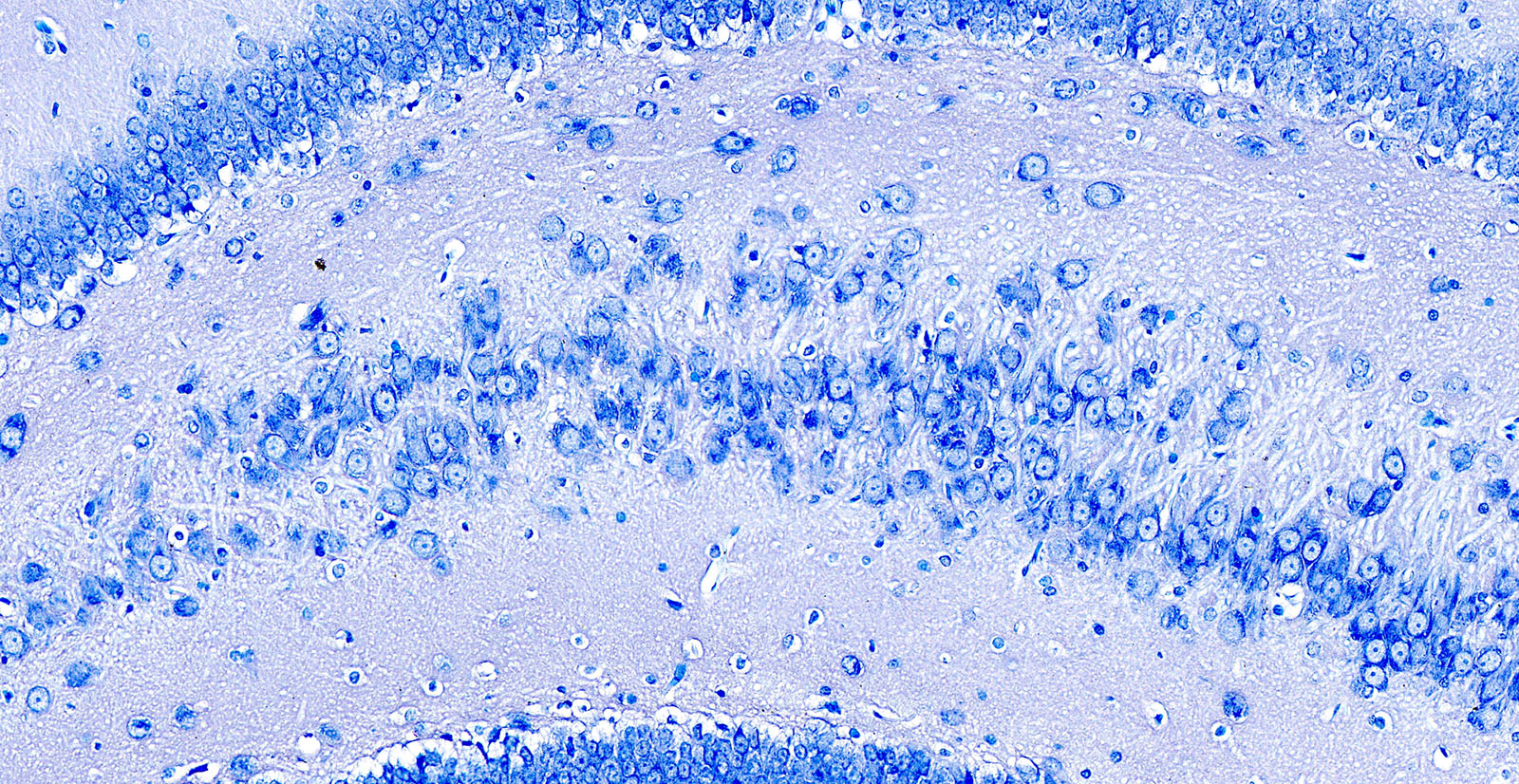

Supplement: Supplementary file 1 [file Data_Sheet_1.zip › Raw data-1/Figure 4-raw data/original images-Nissl/LDR-Nissl CA3c.jpg]

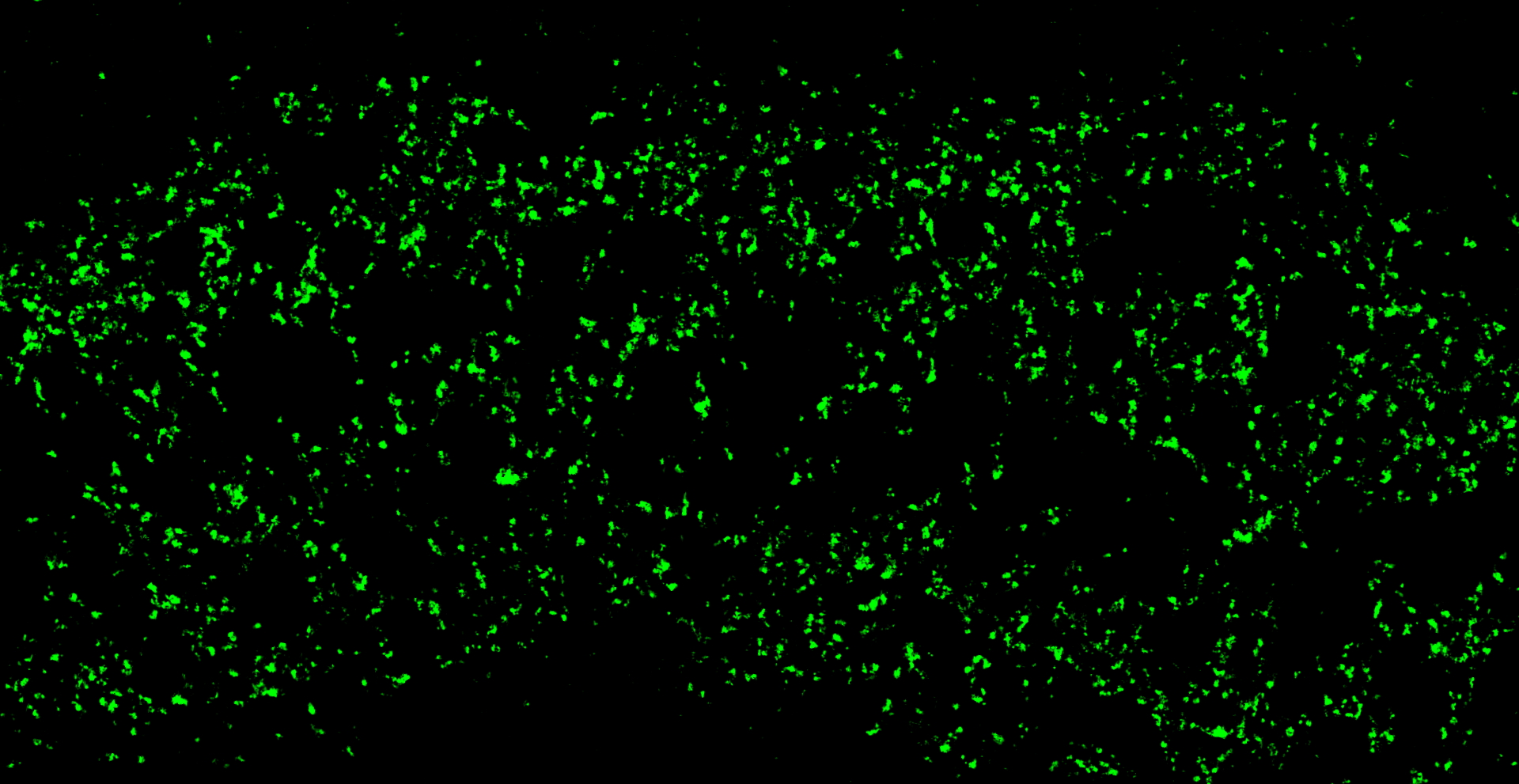

Supplement: Supplementary file 2 [file Data_Sheet_2.zip › Raw data-2/Figure 5-raw data/original images-SYP/control-SYP green.jpg]

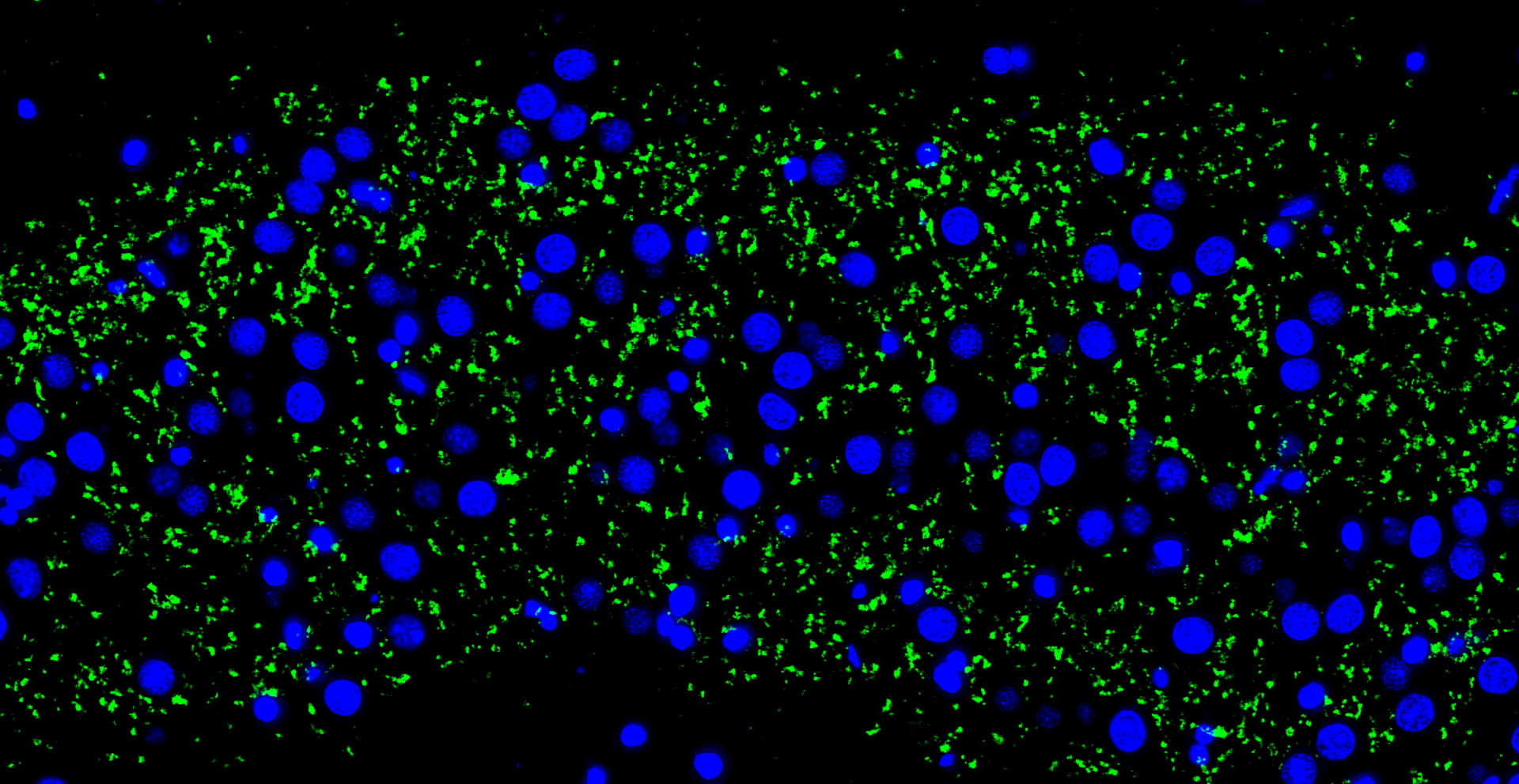

Supplement: Supplementary file 2 [file Data_Sheet_2.zip › Raw data-2/Figure 5-raw data/original images-SYP/control-SYP merge.jpg]

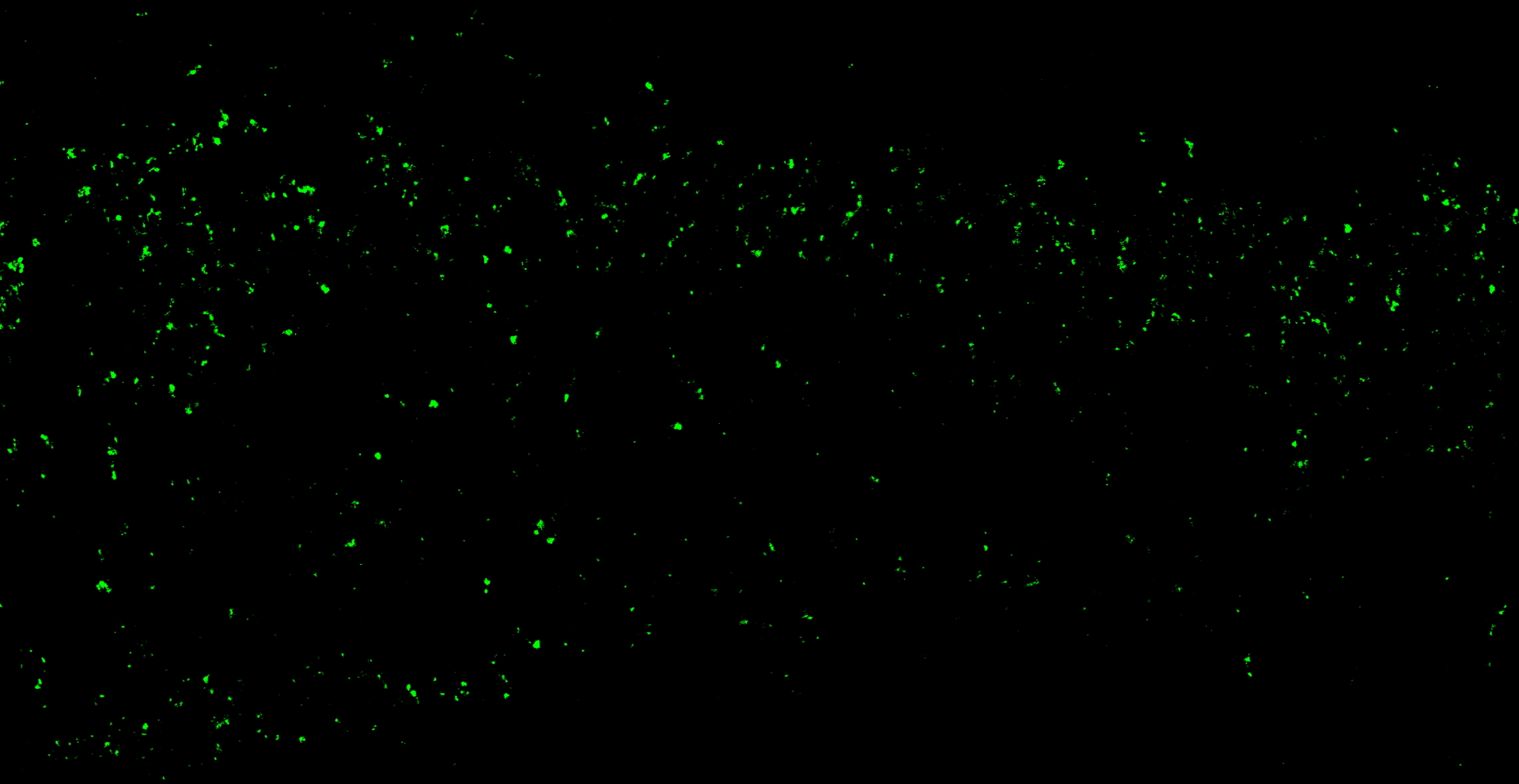

Supplement: Supplementary file 2 [file Data_Sheet_2.zip › Raw data-2/Figure 5-raw data/original images-SYP/HDR-SYP green.jpg]

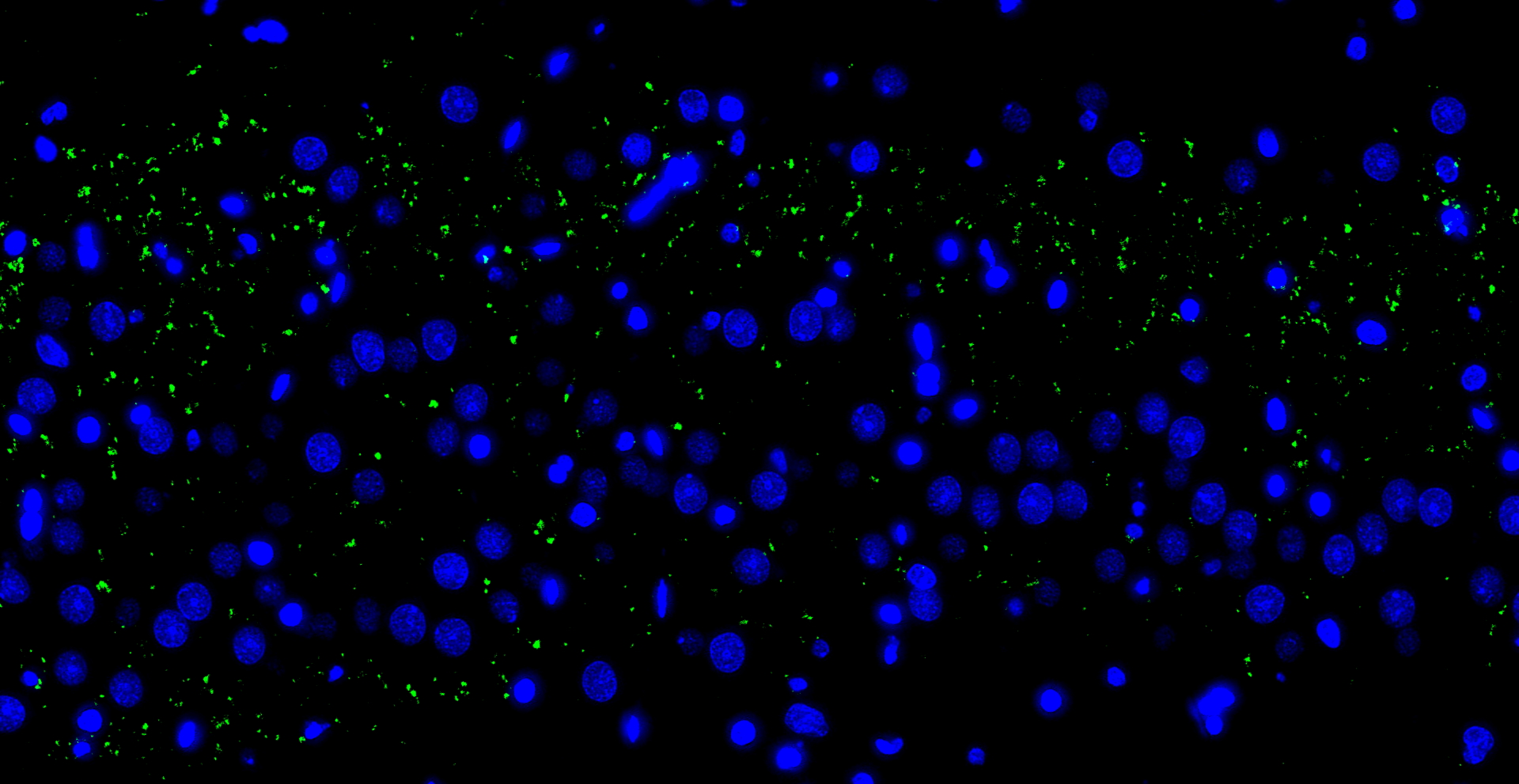

Supplement: Supplementary file 2 [file Data_Sheet_2.zip › Raw data-2/Figure 5-raw data/original images-SYP/HDR-SYP merge.jpg]

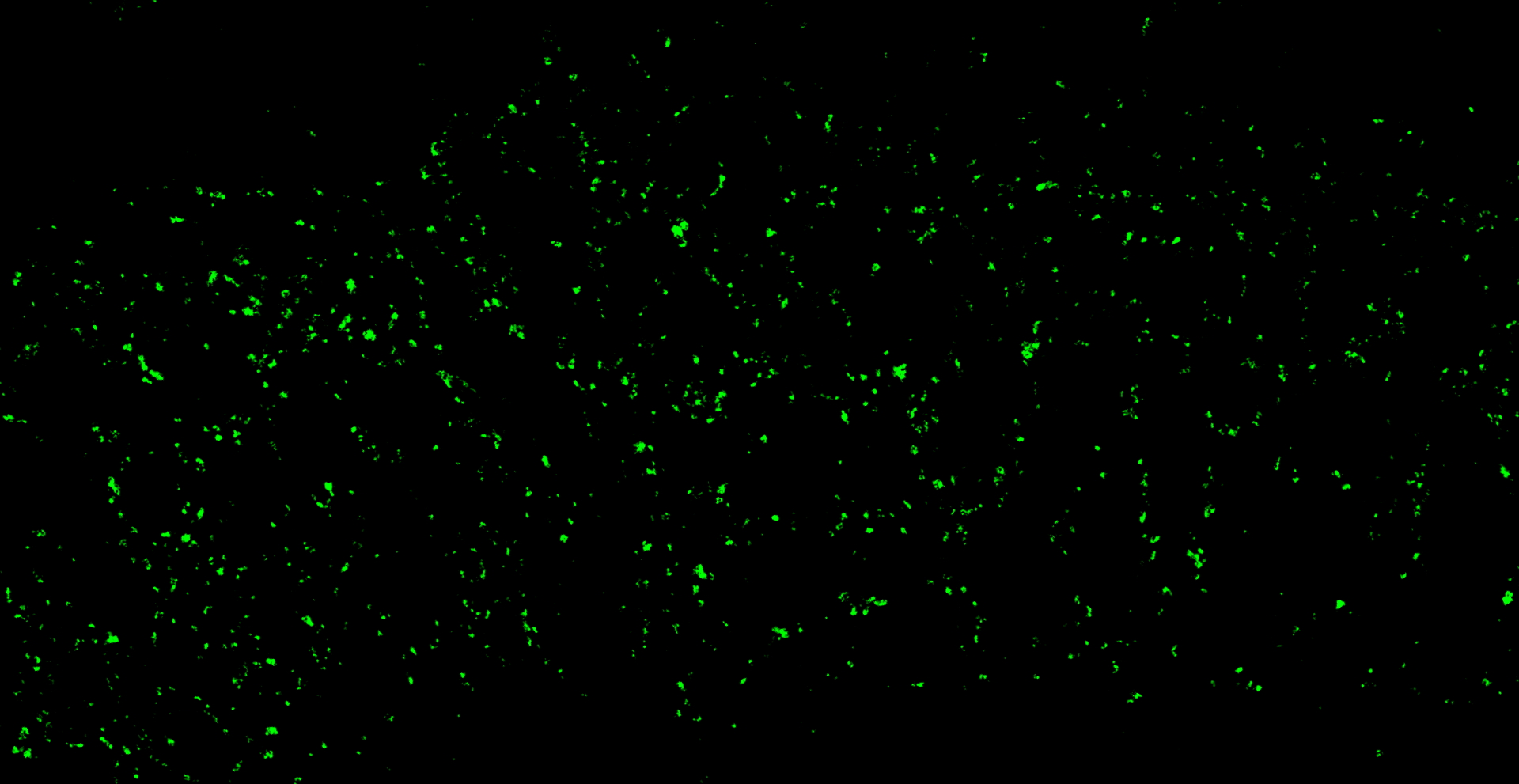

Supplement: Supplementary file 2 [file Data_Sheet_2.zip › Raw data-2/Figure 5-raw data/original images-SYP/LDR-SYP green.jpg]

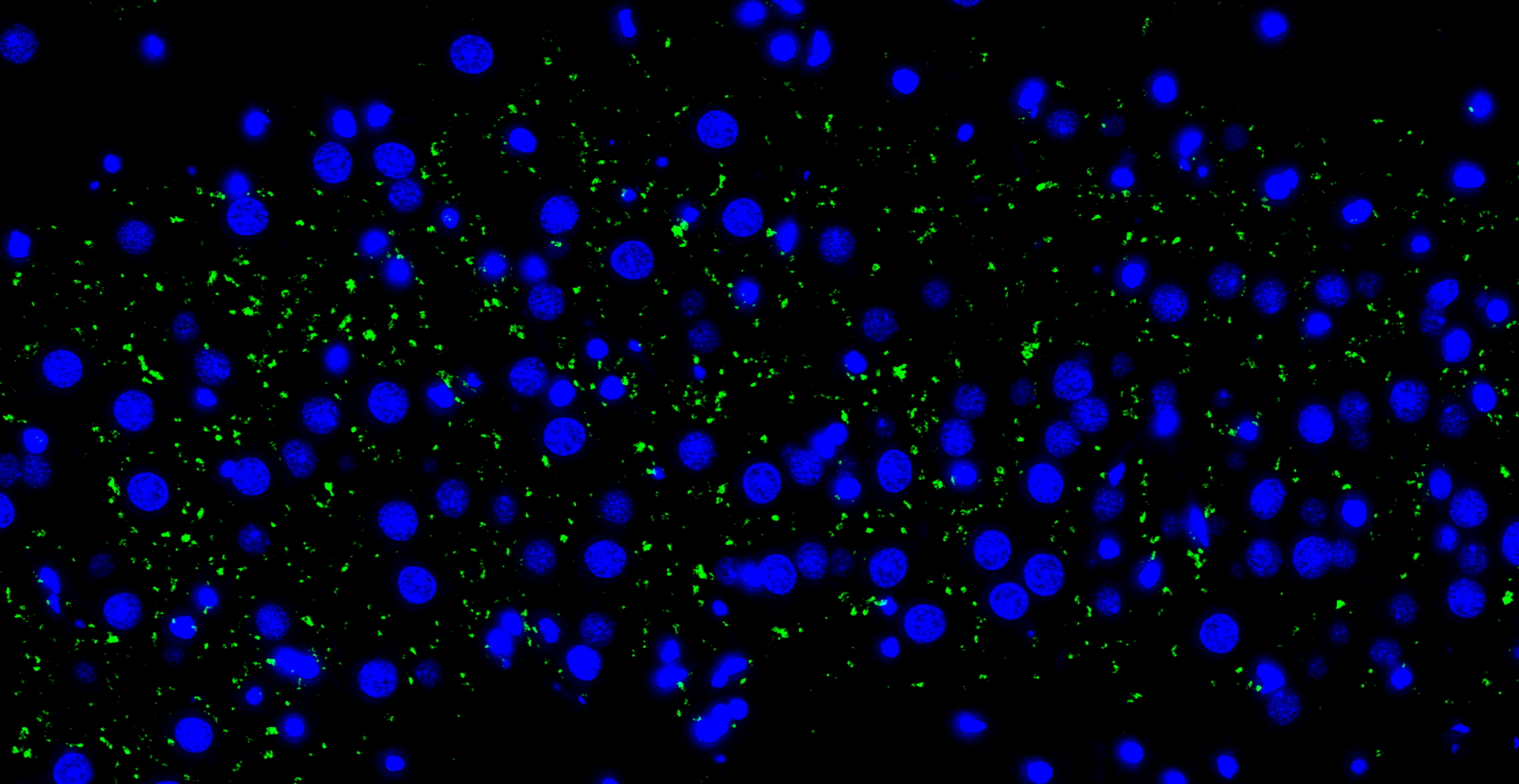

Supplement: Supplementary file 2 [file Data_Sheet_2.zip › Raw data-2/Figure 5-raw data/original images-SYP/LDR-SYP merge.jpg]

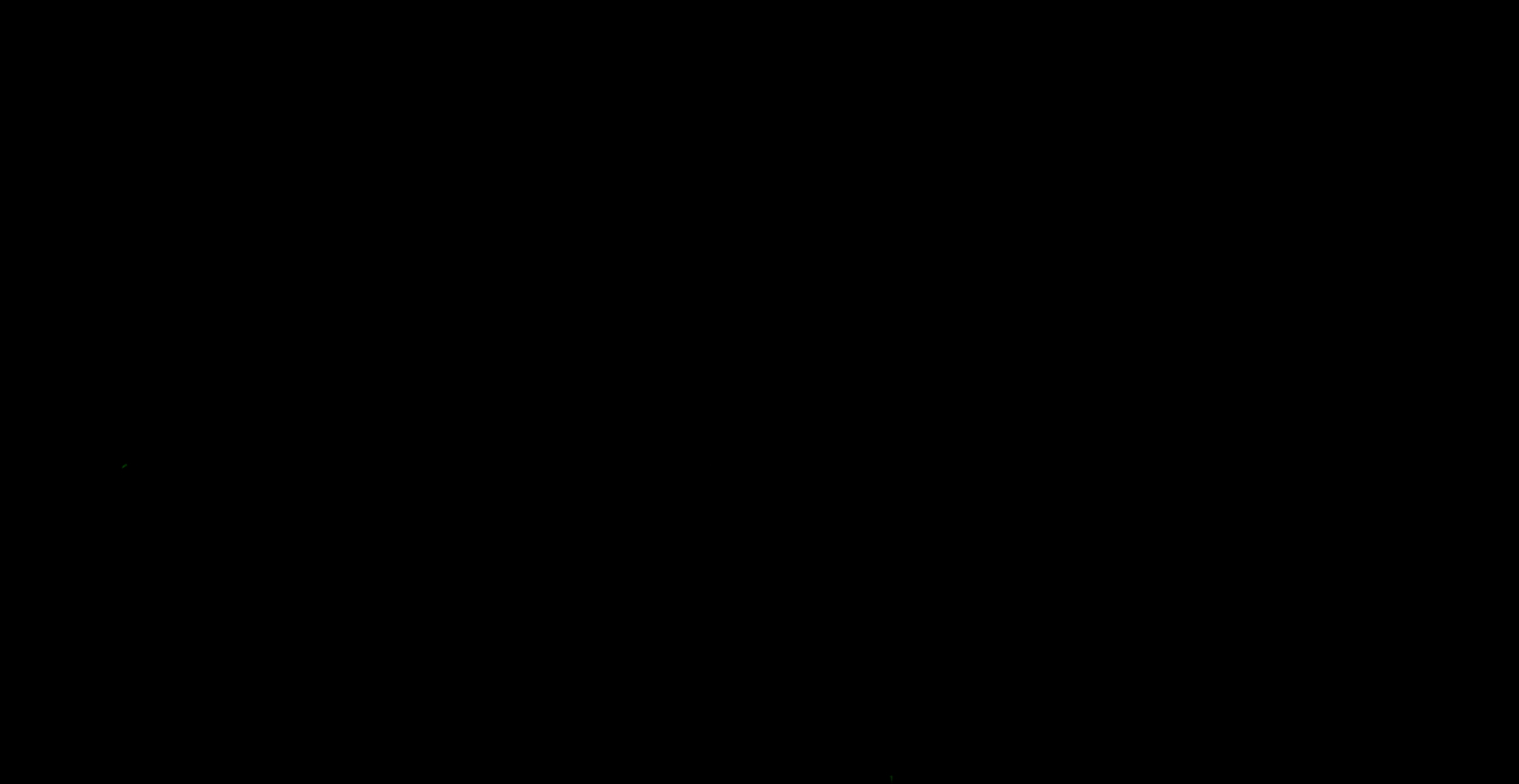

Supplement: Supplementary file 2 [file Data_Sheet_2.zip › Raw data-2/Figure 6-raw data-1/original images-FJB/control-FJB green.jpg]

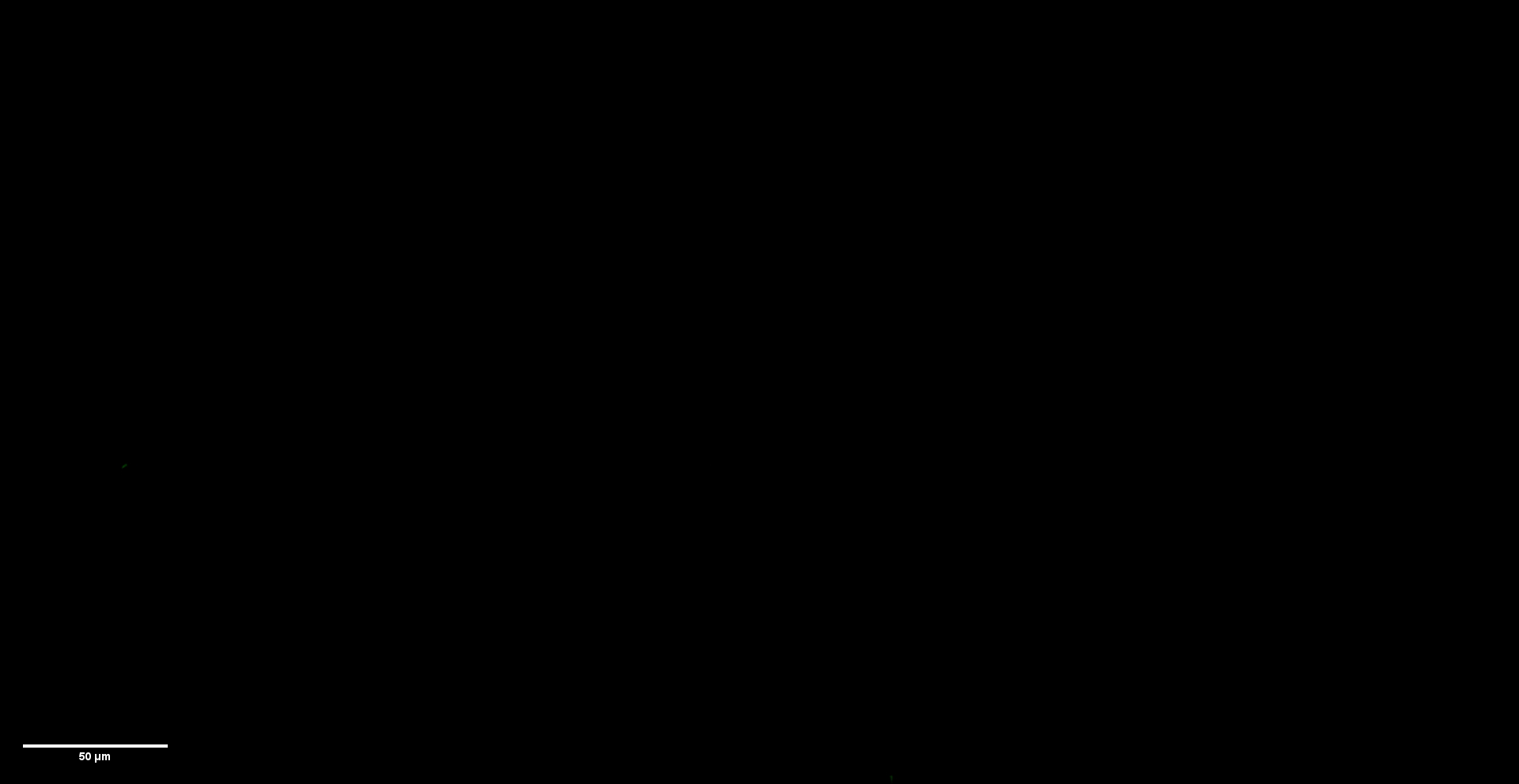

Supplement: Supplementary file 2 [file Data_Sheet_2.zip › Raw data-2/Figure 6-raw data-1/original images-FJB/control-FJB green.png]

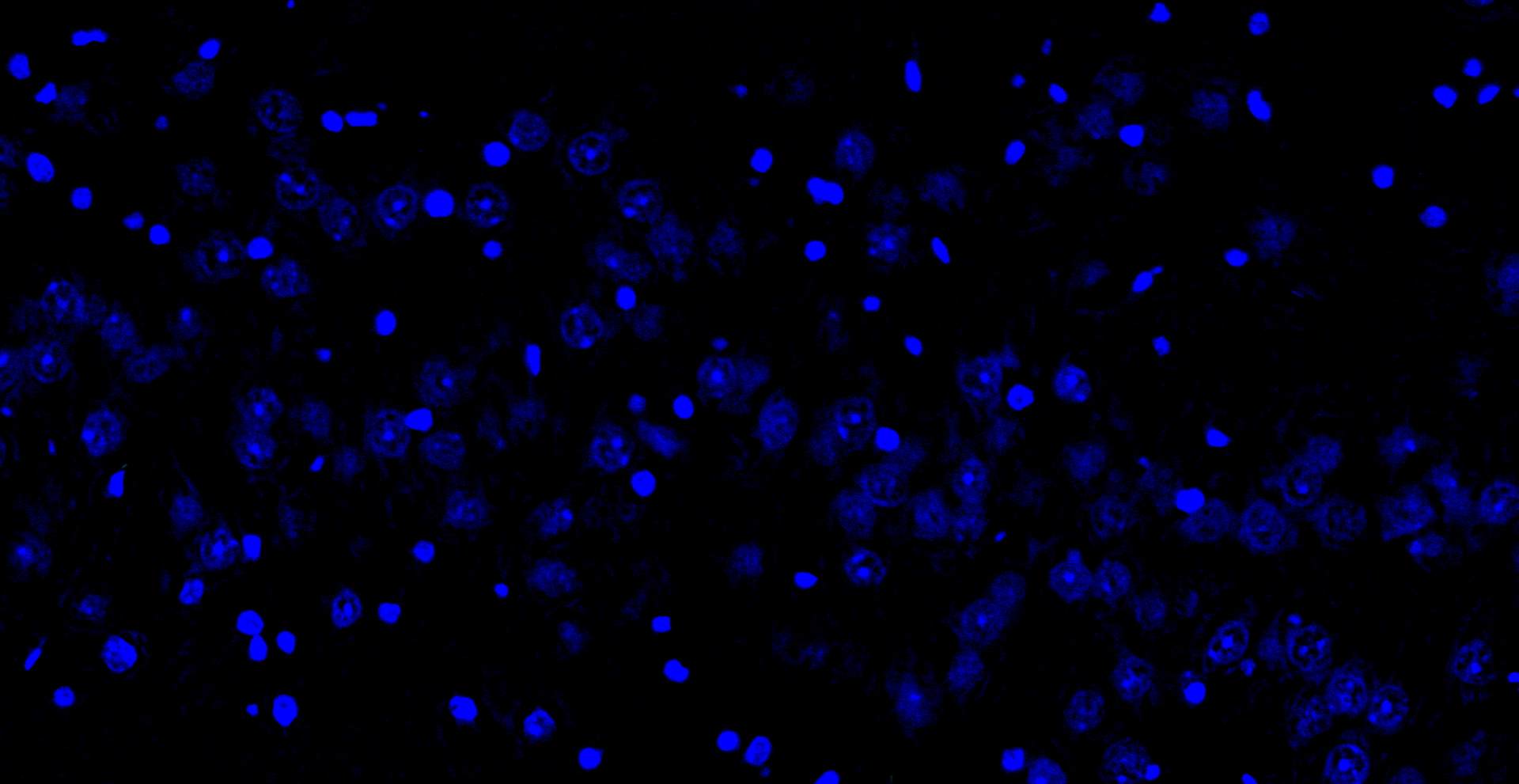

Supplement: Supplementary file 2 [file Data_Sheet_2.zip › Raw data-2/Figure 6-raw data-1/original images-FJB/control-FJB merge.jpg]

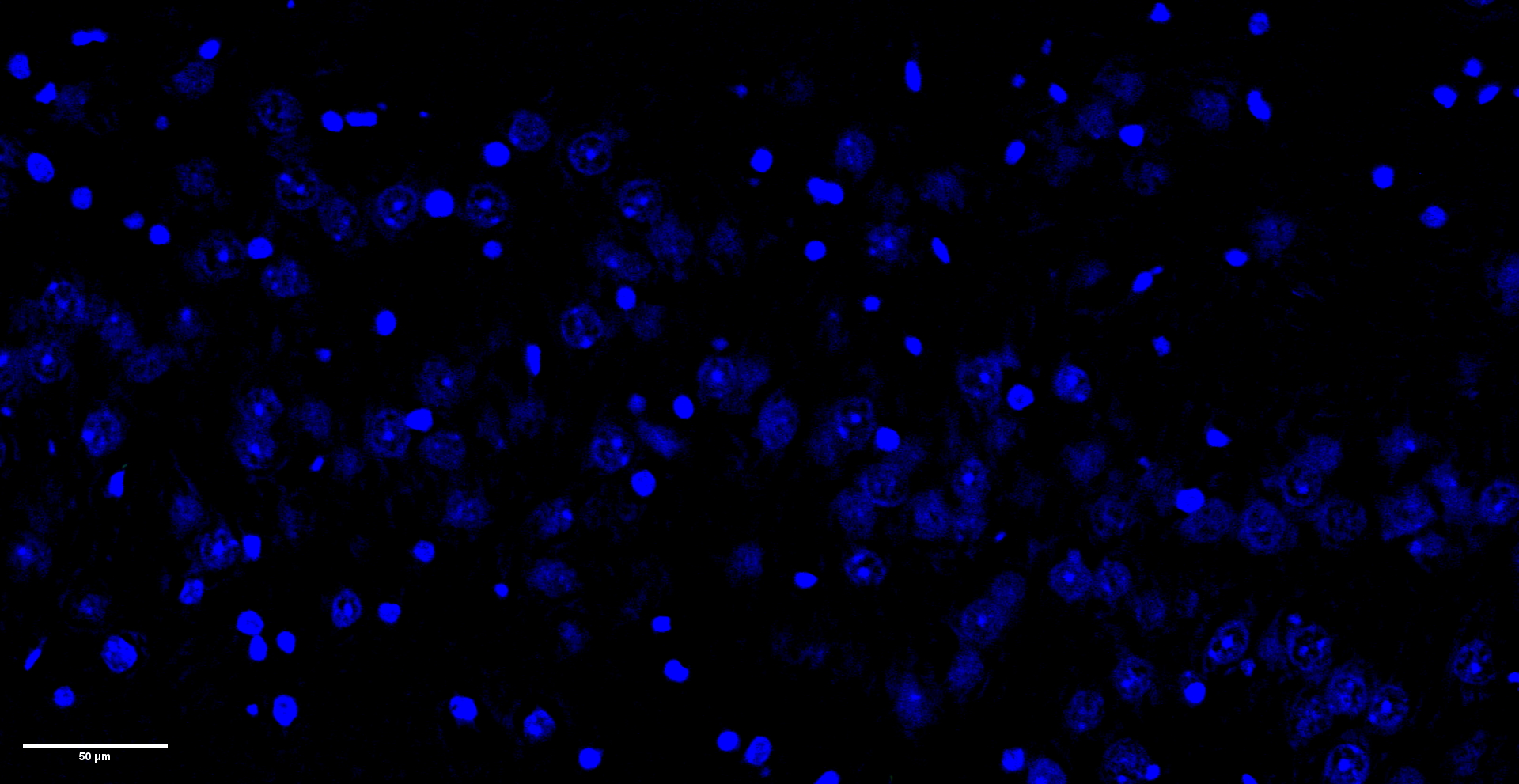

Supplement: Supplementary file 2 [file Data_Sheet_2.zip › Raw data-2/Figure 6-raw data-1/original images-FJB/control-FJB merge.png]

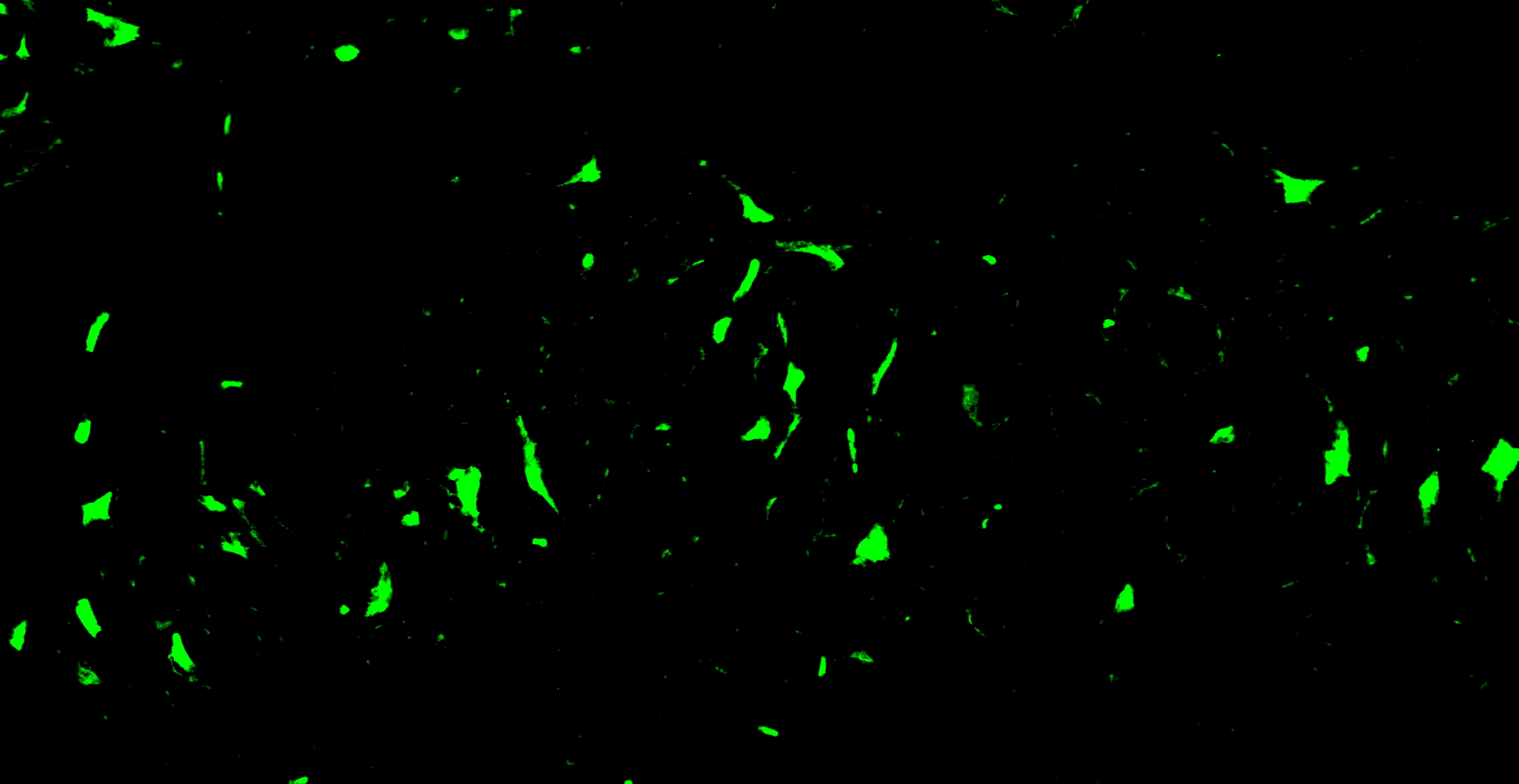

Supplement: Supplementary file 2 [file Data_Sheet_2.zip › Raw data-2/Figure 6-raw data-1/original images-FJB/HDR-FJB green.jpg]

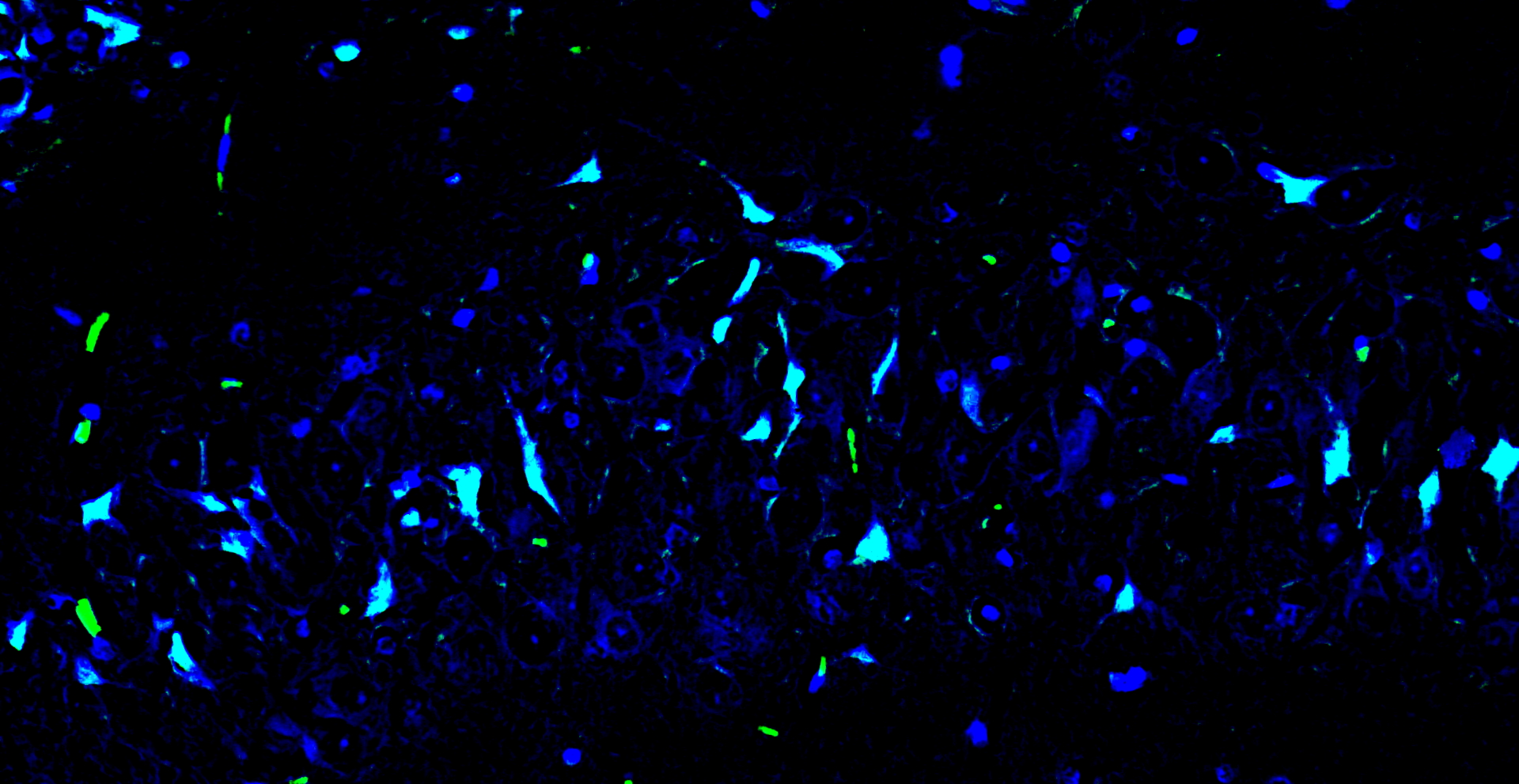

Supplement: Supplementary file 2 [file Data_Sheet_2.zip › Raw data-2/Figure 6-raw data-1/original images-FJB/HDR-FJB merge.jpg]

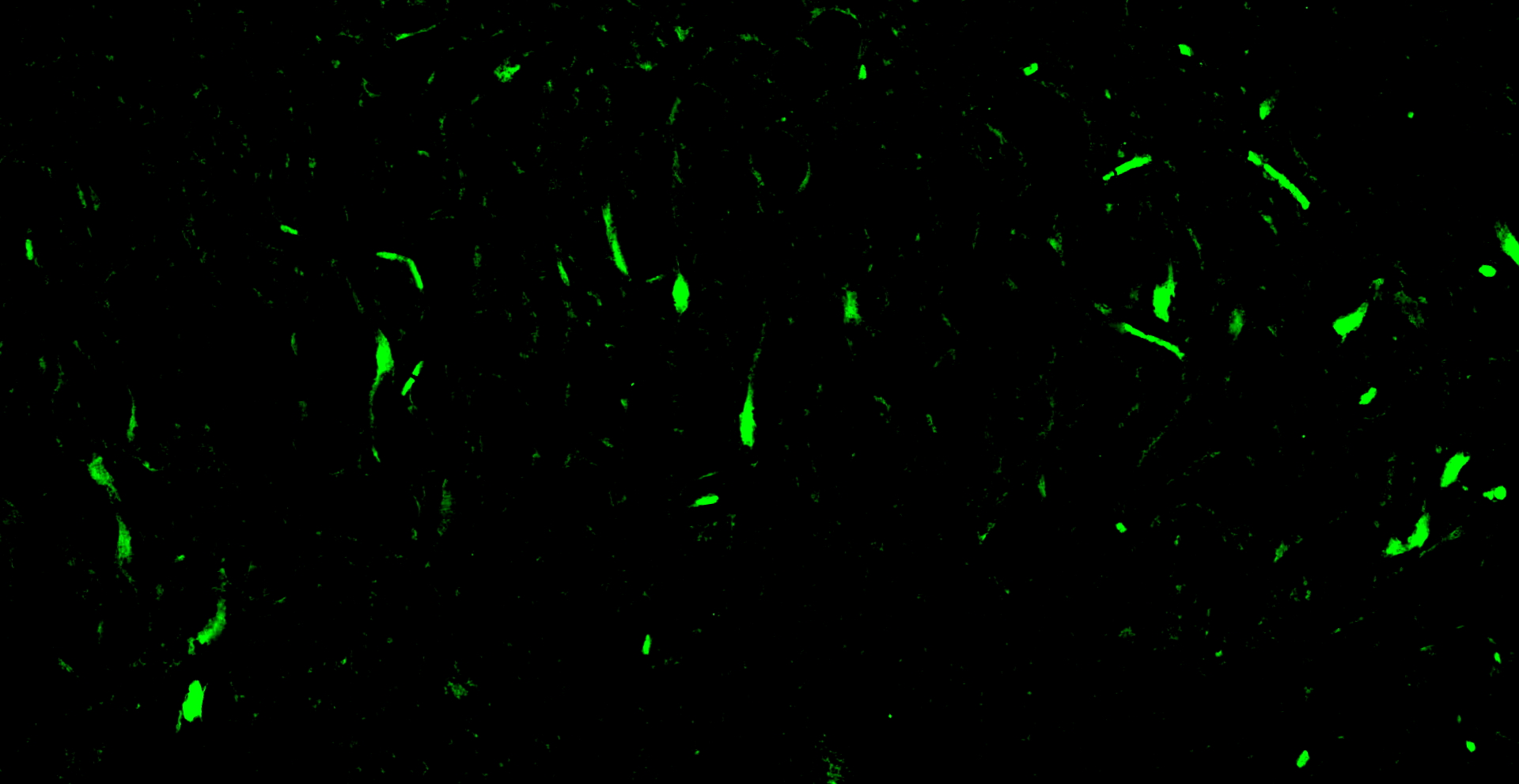

Supplement: Supplementary file 2 [file Data_Sheet_2.zip › Raw data-2/Figure 6-raw data-1/original images-FJB/LDR-FJB green.jpg]

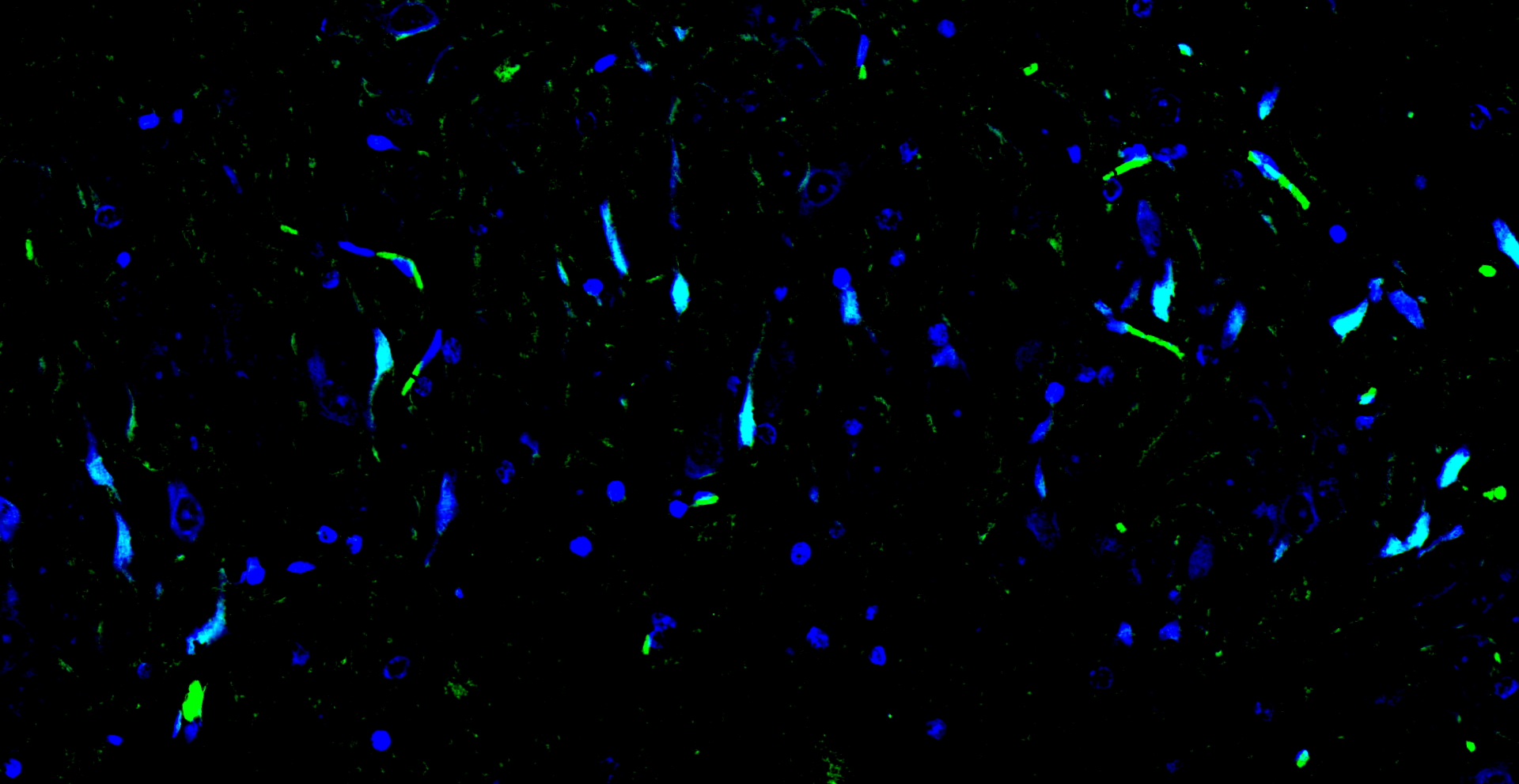

Supplement: Supplementary file 2 [file Data_Sheet_2.zip › Raw data-2/Figure 6-raw data-1/original images-FJB/LDR-FJB merge.jpg]

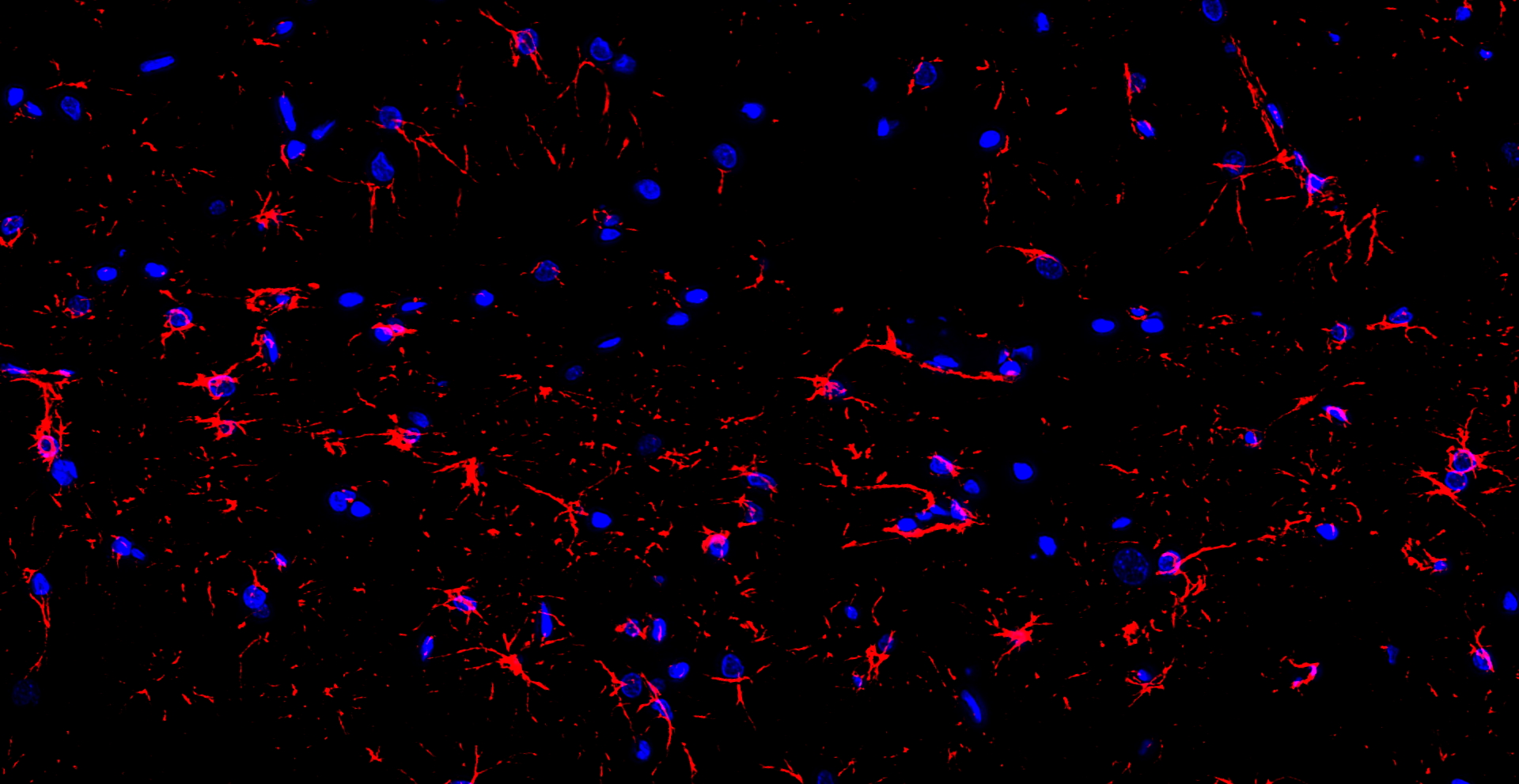

Supplement: Supplementary file 3 [file Data_Sheet_3.zip › Raw data-3/Figure 6-raw data-2/original images-GFAPC3/Control-GFAPC3 merge.jpg]

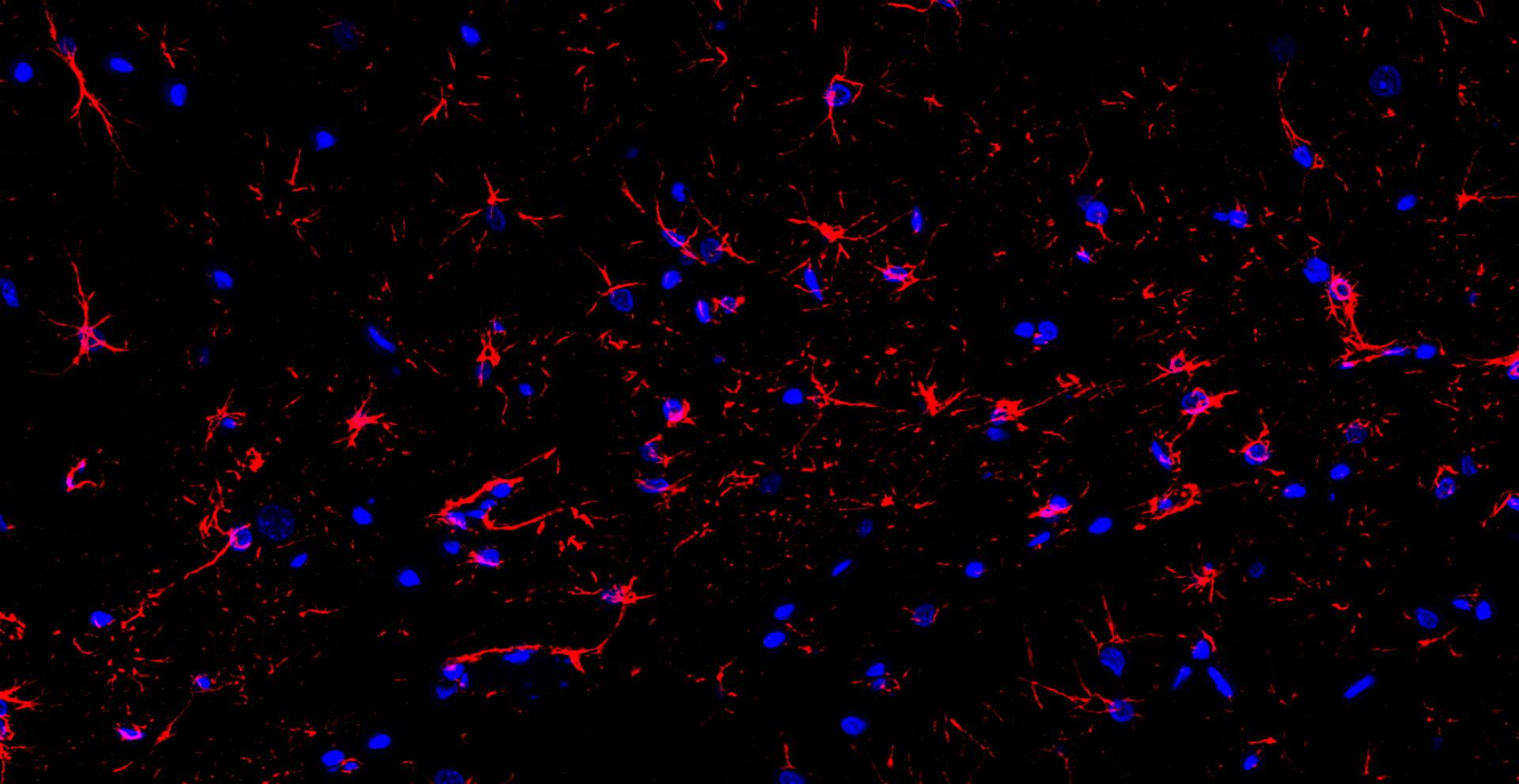

Supplement: Supplementary file 3 [file Data_Sheet_3.zip › Raw data-3/Figure 6-raw data-2/original images-GFAPC3/HDR-GFAPC3 merge.jpg]

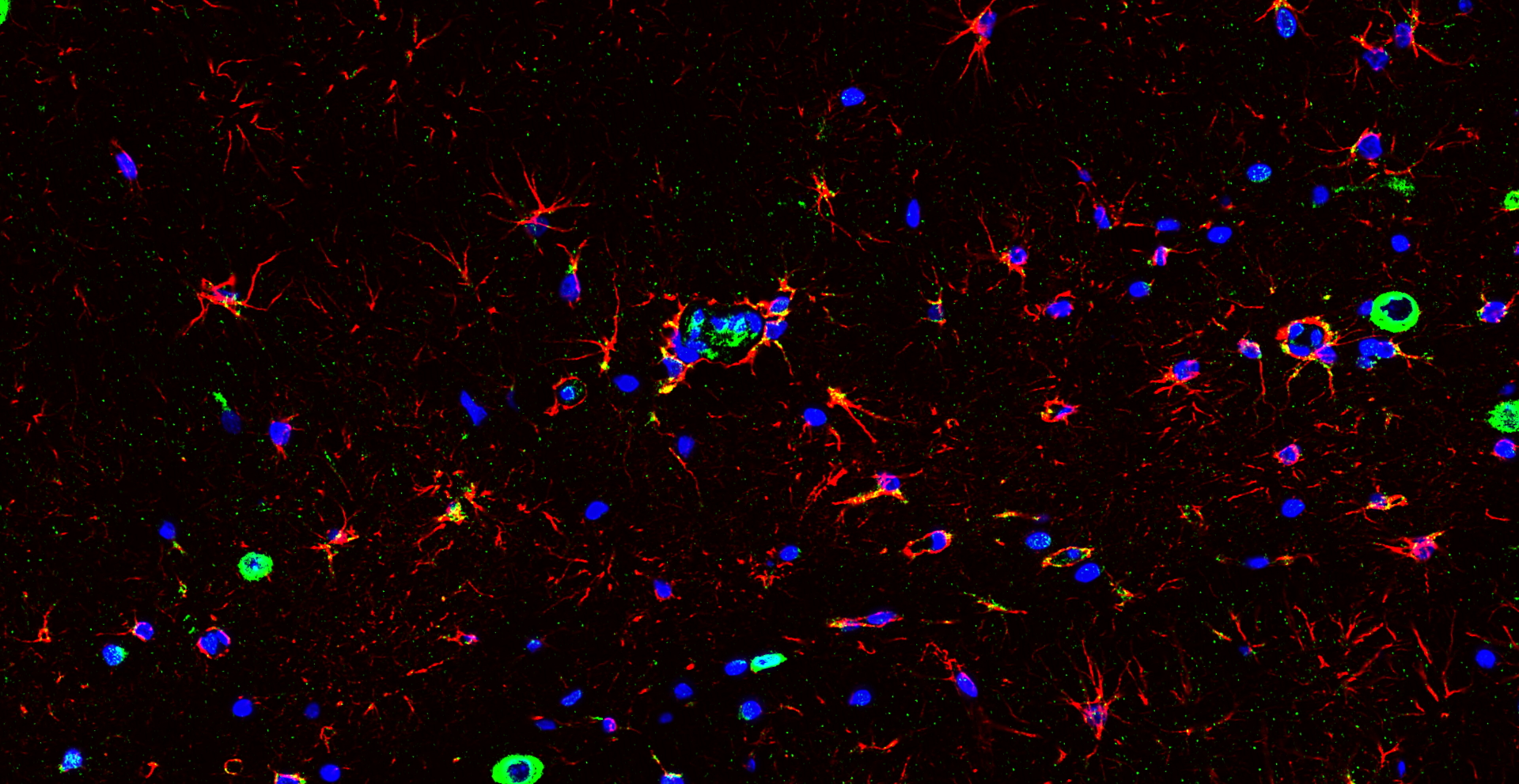

Supplement: Supplementary file 3 [file Data_Sheet_3.zip › Raw data-3/Figure 6-raw data-2/original images-GFAPC3/LDR-GFAPC3 merge.jpg]

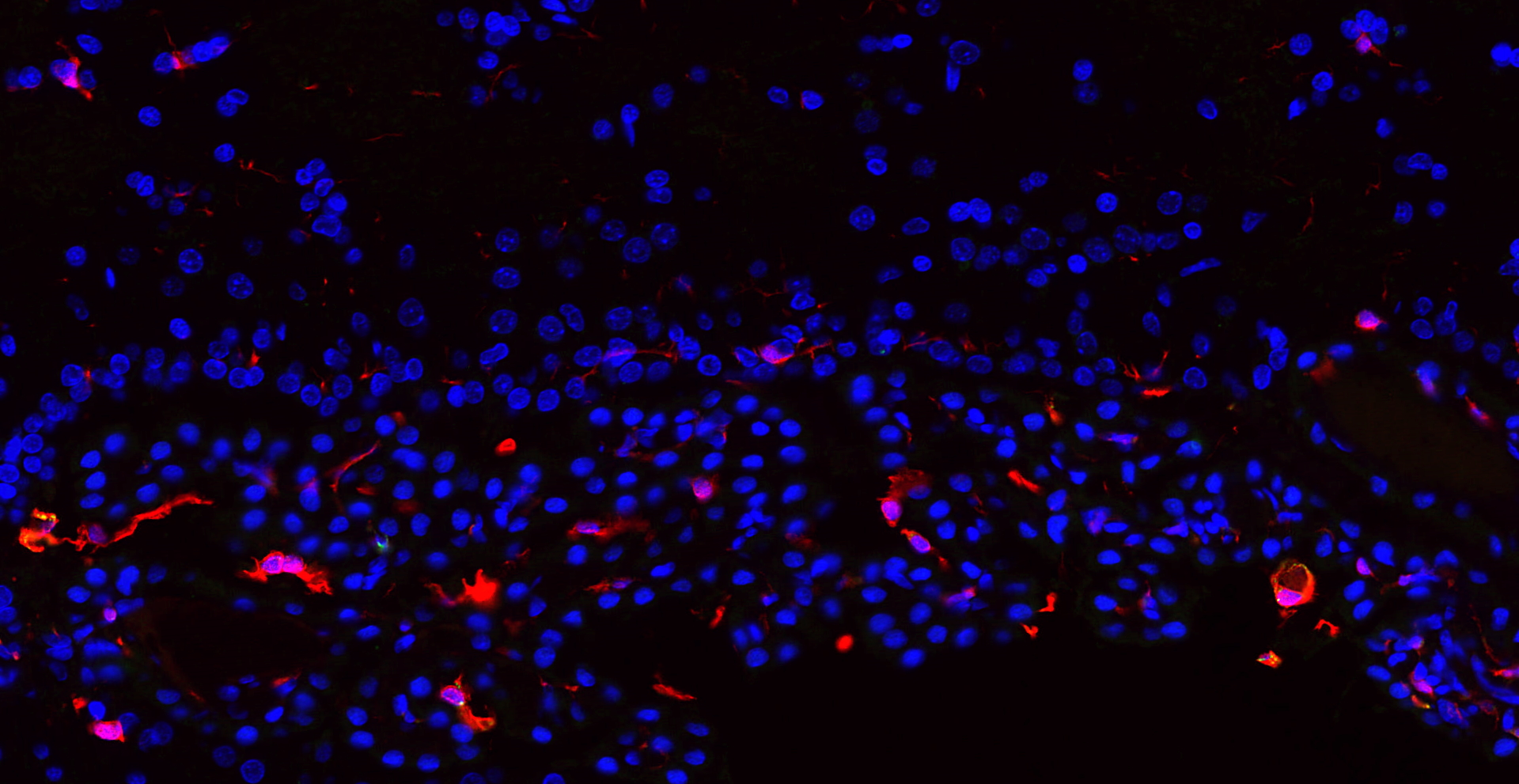

Supplement: Supplementary file 3 [file Data_Sheet_3.zip › Raw data-3/Figure 6-raw data-2/original images-Iba-1CD68/control Iba-1CD68 merge.jpg]

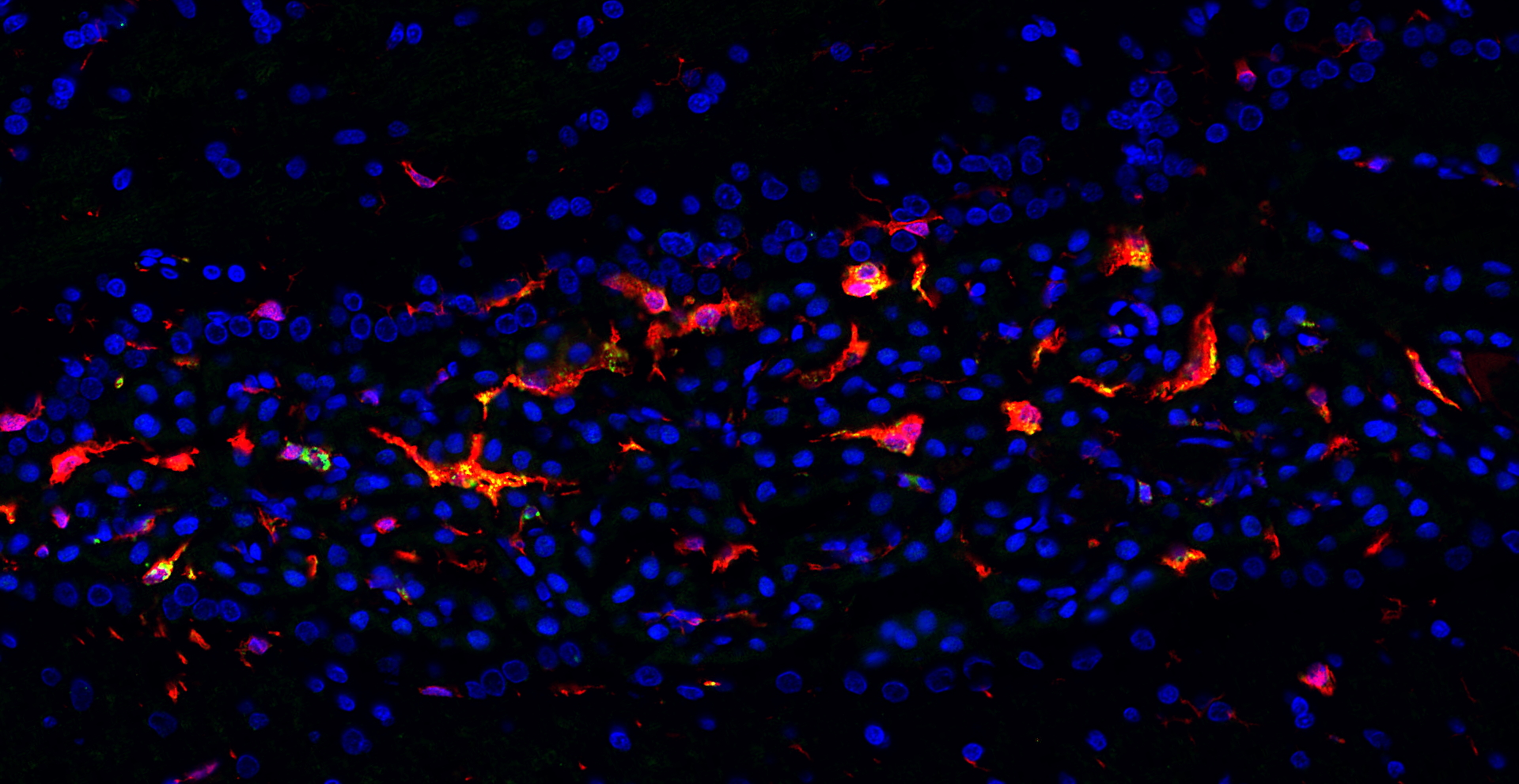

Supplement: Supplementary file 3 [file Data_Sheet_3.zip › Raw data-3/Figure 6-raw data-2/original images-Iba-1CD68/HDR Iba-1CD68 merge.jpg]

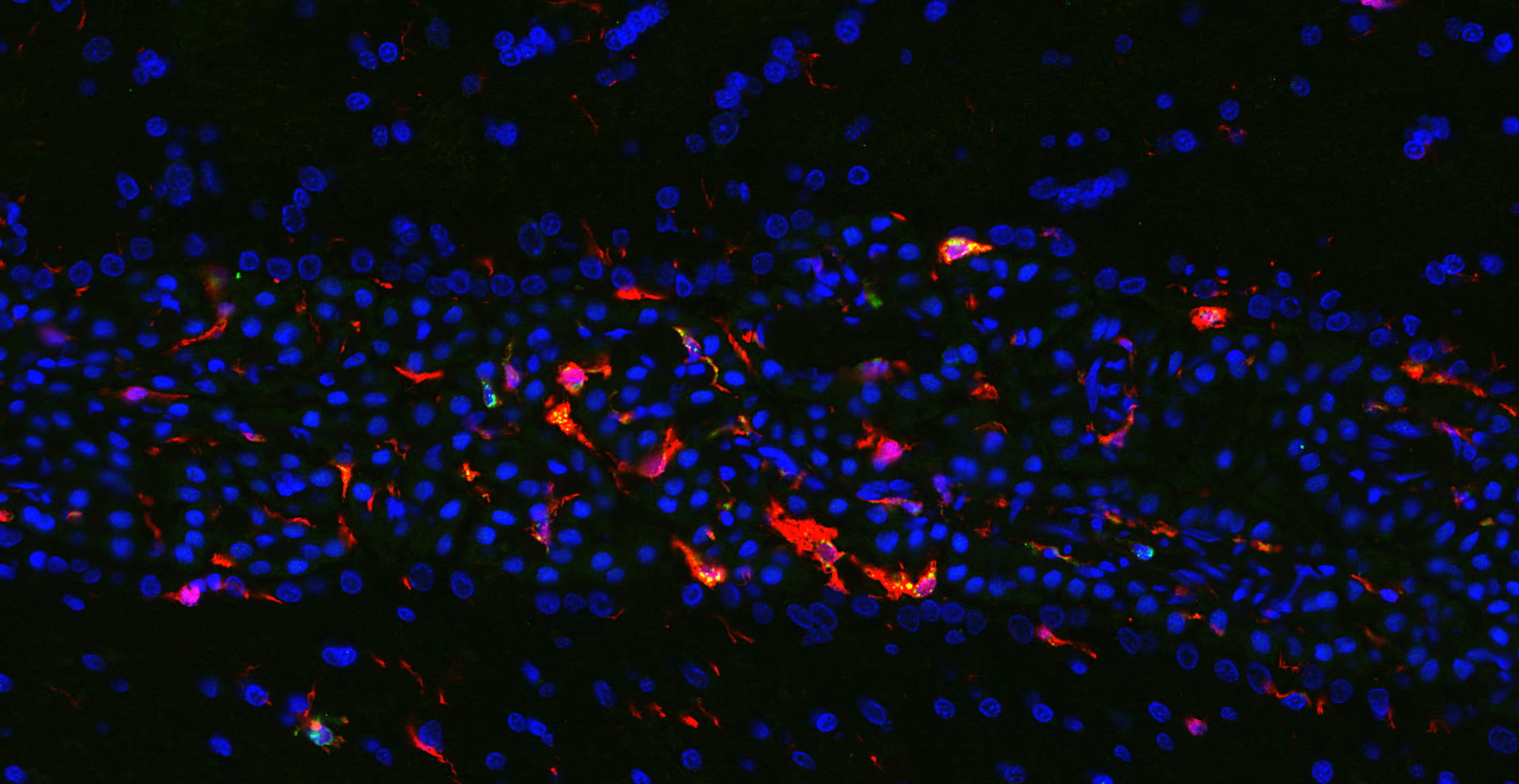

Supplement: Supplementary file 3 [file Data_Sheet_3.zip › Raw data-3/Figure 6-raw data-2/original images-Iba-1CD68/LDR Iba-1CD68 merge.jpg]

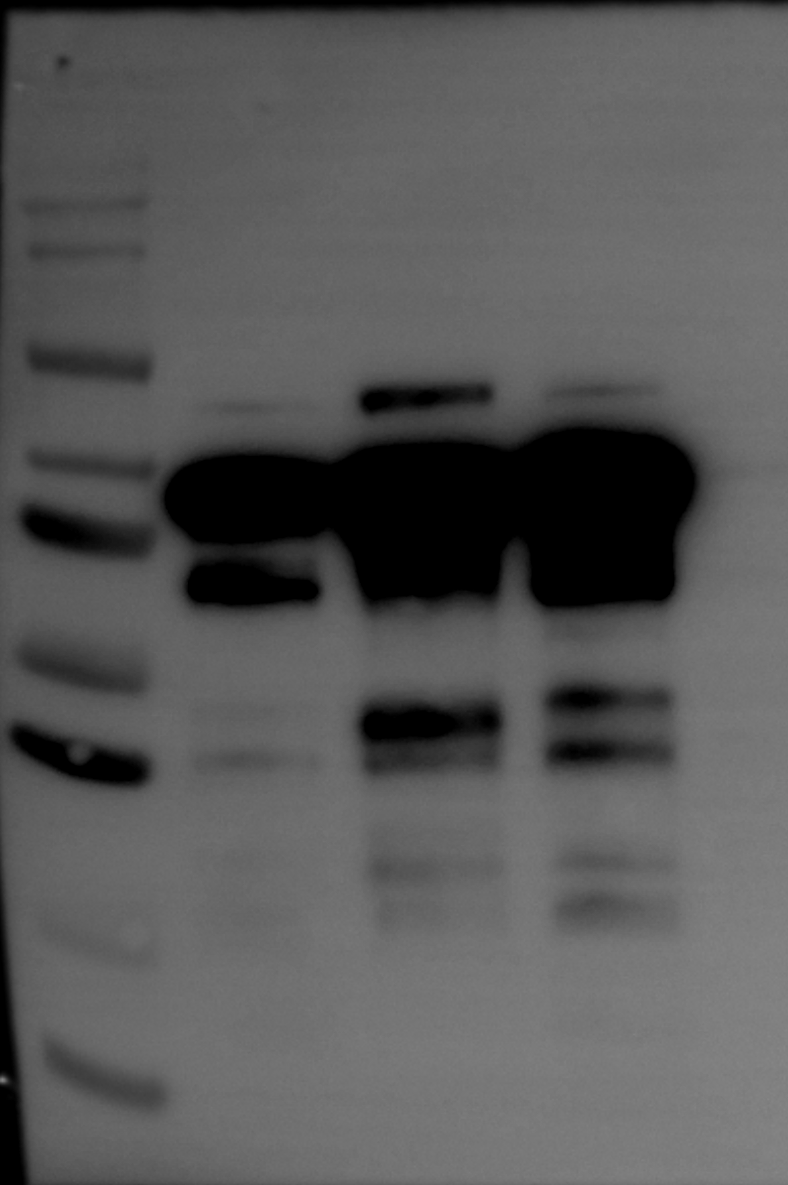

Supplement: Supplementary file 3 [file Data_Sheet_3.zip › Raw data-3/Figure 8-raw data/Akt.tif]

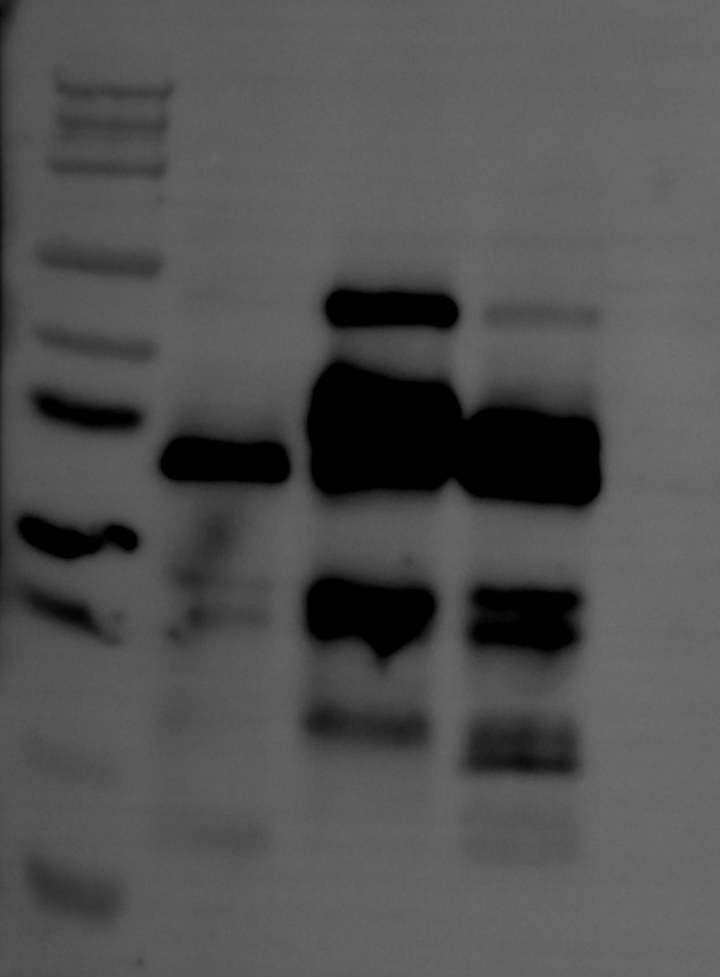

Supplement: Supplementary file 3 [file Data_Sheet_3.zip › Raw data-3/Figure 8-raw data/P-Akt.tif]

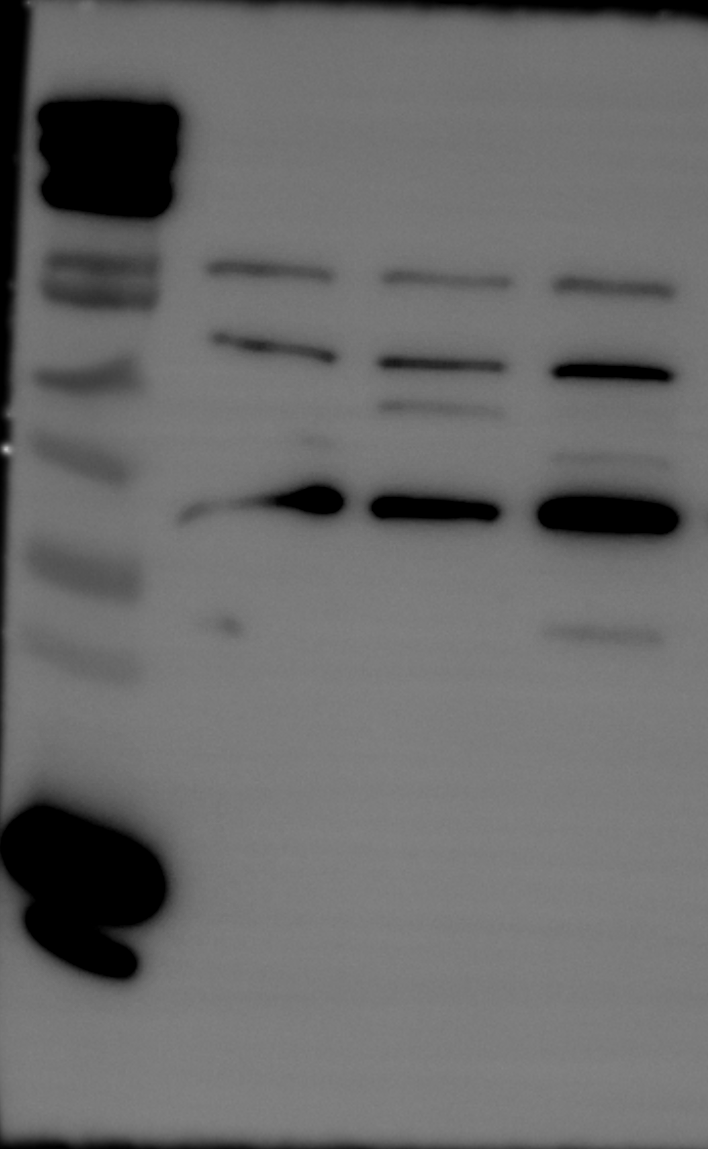

Supplement: Supplementary file 3 [file Data_Sheet_3.zip › Raw data-3/Figure 8-raw data/p-PI3K.tif]

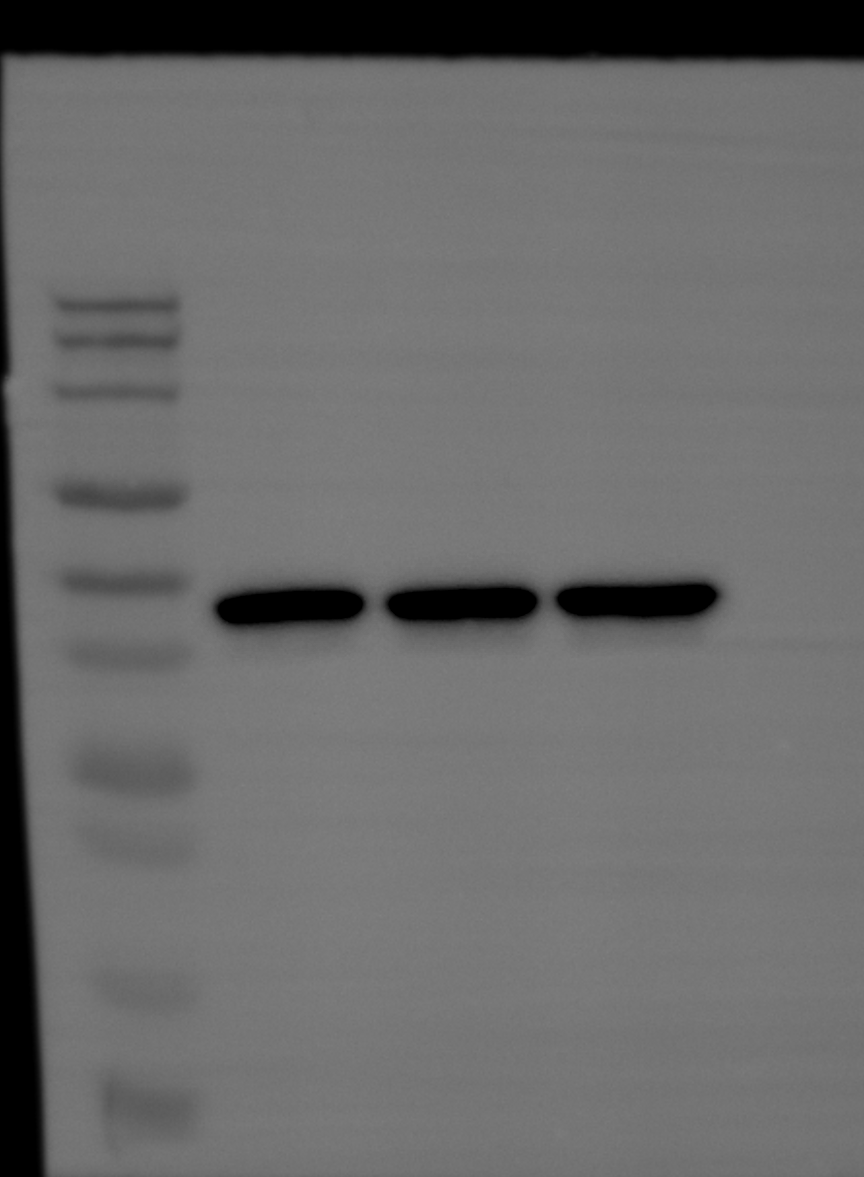

Supplement: Supplementary file 3 [file Data_Sheet_3.zip › Raw data-3/Figure 8-raw data/β-actin.tif]
